# The universal suppressor mutation restores membrane budding defects in the HSV-1 nuclear egress complex by stabilizing the oligomeric lattice

**DOI:** 10.1371/journal.ppat.1011936

**Published:** 2024-01-16

**Authors:** Elizabeth B. Draganova, Hui Wang, Melanie Wu, Shiqing Liao, Amber Vu, Gonzalo L. Gonzalez-Del Pino, Z. Hong Zhou, Richard J. Roller, Ekaterina E. Heldwein

**Affiliations:** 1 Department of Molecular Biology and Microbiology, Tufts University School of Medicine, Boston, Massachusetts, United States of America; 2 Department of Microbiology, Immunology & Molecular Genetics, University of California, Los Angeles (UCLA), Los Angeles, California, United States of America; 3 Department of Bioengineering, UCLA, Los Angeles, California, United States of America; 4 California NanoSystems Institute, UCLA, Los Angeles, California, United States of America; 5 School of Chemistry and Molecular Biosciences, The University of Queensland, Brisbane, Australia; 6 Department of Microbiology and Immunology, Carver College of Medicine, University of Iowa, Iowa City, Iowa, United States of America; Wistar Institute, UNITED STATES

## Abstract

Nuclear egress is an essential process in herpesvirus replication whereby nascent capsids translocate from the nucleus to the cytoplasm. This initial step of nuclear egress–budding at the inner nuclear membrane–is coordinated by the nuclear egress complex (NEC). Composed of the viral proteins UL31 and UL34, NEC deforms the membrane around the capsid as the latter buds into the perinuclear space. NEC oligomerization into a hexagonal membrane-bound lattice is essential for budding because NEC mutants designed to perturb lattice interfaces reduce its budding ability. Previously, we identified an NEC suppressor mutation capable of restoring budding to a mutant with a weakened hexagonal lattice. Using an established *in-vitro* budding assay and HSV-1 infected cell experiments, we show that the suppressor mutation can restore budding to a broad range of budding-deficient NEC mutants thereby acting as a universal suppressor. Cryogenic electron tomography of the suppressor NEC mutant lattice revealed a hexagonal lattice reminiscent of wild-type NEC lattice instead of an alternative lattice. Further investigation using x-ray crystallography showed that the suppressor mutation promoted the formation of new contacts between the NEC hexamers that, ostensibly, stabilized the hexagonal lattice. This stabilization strategy is powerful enough to override the otherwise deleterious effects of mutations that destabilize the NEC lattice by different mechanisms, resulting in a functional NEC hexagonal lattice and restoration of membrane budding.

## Author summary

Herpesviruses infect most of the human population, establishing lifelong infections that periodically reactivate. Successful infection of a host requires the formation of new viral particles within host cells. Early in viral assembly, capsids are translocated from the nucleus into the cytoplasm. This process of capsid nuclear egress is mediated by the virally encoded nuclear egress complex (NEC) that deforms the membrane around the capsid as it oligomerizes into a hexagonal lattice. Mutations that disrupt the lattice can block nuclear egress and reduce viral progeny production, making the NEC an attractive therapeutic target. However, the virus can overcome the inhibitory effects of such mutations through suppressor mutations. Here, we show that one such suppressor mutation promotes new lattice contacts that are expected to strengthen the lattice. We hypothesize that the NEC can fine-tune its lattice in response to external perturbations, be they natural such as membrane curvature, or artificial such as mutations. Any therapeutic approach that targets NEC lattice formation must consider its intrinsic flexibility.

## Introduction

Viruses are experts at reorganizing host membranes to traffic their capsids across the compartmentalized interior of eukaryotic cells. One of the more unusual mechanisms of membrane manipulation is found in *Herpesvirales*, which is an order of large, enveloped viruses that infect multiple species across the animal kingdom and cause life-long infections in the majority of the world’s population [reviewed in [1]]. Replication of herpesviral dsDNA genomes and their subsequent packaging into capsids occurs within the nucleus [reviewed in [2,3]]. Genome-containing capsids are then transported into the cytoplasm for maturation into infectious virions. Most of the nucleocytoplasmic traffic occurs through the nuclear pores, but at ~125 nm in diameter, herpesviral capsids are too large to fit through the ~40-nm nuclear pore opening [4]. Instead, the capsids use a complex, non-canonical nuclear transport route termed nuclear egress [reviewed in [2,3,5]]. First, they dock at the inner nuclear membrane (INM) and bud into the perinuclear space, producing perinuclear enveloped virions (a stage termed primary envelopment). The envelopes of these intermediates then fuse with the outer nuclear membrane (ONM) and capsids are then released into the cytoplasm (a stage termed de-envelopment).

The nuclear egress mechanism is best understood for the family *Herpesviridae*, commonly referred to as herpesviruses, which infect mammals, birds, and reptiles. Two conserved viral proteins, called UL31 and UL34 in herpes simplex virus (HSV), are essential for nuclear egress in herpesviruses. UL31 is a soluble nuclear phosphoprotein [6, 7] whereas UL34 is a type I membrane protein containing a single C-terminal transmembrane helix [6, 8]. Together, UL31 and UL34 form the heterodimeric nuclear egress complex (NEC) that is anchored in the INM and faces the nuclear interior. Both proteins are essential for nuclear egress, and in the absence of either, capsids become trapped within the nucleus, which results in greatly reduced viral titers [6,7,9–13]. Moreover, overexpression of both UL31 and UL34 in uninfected cells causes the accumulation of empty budded vesicles in the perinuclear space, which implies that the NEC is not only necessary but also sufficient for the INM budding [14–18]. Collectively, these findings highlight the central role of the NEC during nuclear egress.

Recent studies with purified recombinant NEC and synthetic lipid vesicles have shown that several NEC homologs can deform and bud membranes *in vitro* in the absence of added energy or other proteins. These include the NECs from herpes simplex virus 1 (HSV-1) [19], a prototypical herpesvirus that infects much of the world’s population; the closely related pseudorabies virus (PRV) that infects animals [20]; and the more distantly related Epstein-Barr Virus (EBV) [21], a nearly ubiquitous human herpesvirus.

The NEC oligomerizes into membrane-bound coats on the inner surface of the budded vesicles. Hexagonal coats resembling a honeycomb have been observed by cryogenic electron microscopy/tomography (cryoEM/ET) on vesicles formed by recombinant HSV-1 NEC *in vitro* [19], vesicles formed in uninfected cells overexpressing PRV NEC [22], and in perinuclear vesicles formed in HSV-1-infected cells [23]. Interestingly, crystallized NEC homologs from HSV-1 [24] and human cytomegalovirus (HCMV) [25] also formed hexagonal crystal lattices of geometry and dimensions similar to those observed in the membrane-bound NEC coats. Finally, EBV NEC also forms membrane-bound coats *in vitro* but their geometry is yet unclear [21]. Both the intrinsic membrane budding ability and the formation of oligomeric coats thus appear to be conserved among the NEC homologs.

In HSV-1 NEC, oligomerization into the hexagonal lattice is essential for budding. Mutations targeting lattice interfaces within the NEC hexamers (hexameric) or between hexamers (interhexameric) cause budding defects *in vitro* [19,24] and reduce nuclear egress in infected cells [26,27]. The first such mutation, which changed D35 and E37 in UL34 to alanine (A), was identified in a mutational screen targeting charge clusters in the HSV-1 UL34 sequence [28]. Recognizing that the mutations are in the gene rather than the protein, for clarity, we will refer to the proteins translated from the mutant genes as mutant proteins designated by the altered amino acids (in this case, as the D35A_UL34_/E37A_UL34_ mutant). This double mutation reduced viral titers by ~3 orders of magnitude to levels of UL34-null mutant HSV-1 and blocked capsid egress from the nucleus in a dominant-negative manner [27]. Therefore, we refer to it as DN_UL34_. The mutation did not affect the NEC formation, its localization to the INM, or capsid docking at the INM but, instead, precluded capsid budding [27]. Furthermore, purified recombinant NEC-DN_UL34_ bound synthetic membranes *in vitro* but had minimal membrane-budding activity and did not form hexagonal coats on membranes [19].

Interestingly, the nuclear budding defect due to the DN_UL34_ mutation could be suppressed by an extragenic mutation in HSV-1 UL31, R229L_UL31_, which arose during serial passaging of the DN_UL34_ mutant HSV-1 virus on a UL34-complementing cell line [27] through directed viral evolution [reviewed in [29,30]]. We refer to this mutation as SUP_UL31_. Residue R229_UL31_ is located near the interhexameric interface, far away from the DN_UL34_ mutations at the hexameric interface [24], making it unclear how the SUP_UL31_ mutation restores DN_UL34_ nuclear budding defects.

Here, we show that the SUP_UL31_ mutation can restore efficient budding to a broad range of mutants that disrupt important functional interfaces, acting as a “universal” suppressor of budding defects. Using cryogenic electron tomography (cryoET) and x-ray crystallography, we show that the SUP_UL31_ mutation does not change the structure of the NEC heterodimer or its oligomerization into hexamers. Instead, it promotes the formation of new contacts at the interhexameric interface. The increased interhexameric interface would reinforce the hexagonal NEC lattice, helping it to counteract the lattice-destabilizing effects of mutations. We hypothesize that its dynamic nature allows the NEC lattice to adapt to perturbations that it encounters during nuclear egress, for example, changes in membrane curvature or capsid interactions, and maintain its function.

## Results

### The SUP_UL31_ mutation restores membrane budding *in vitro* to various oligomeric interface mutants

**Fig 1:**
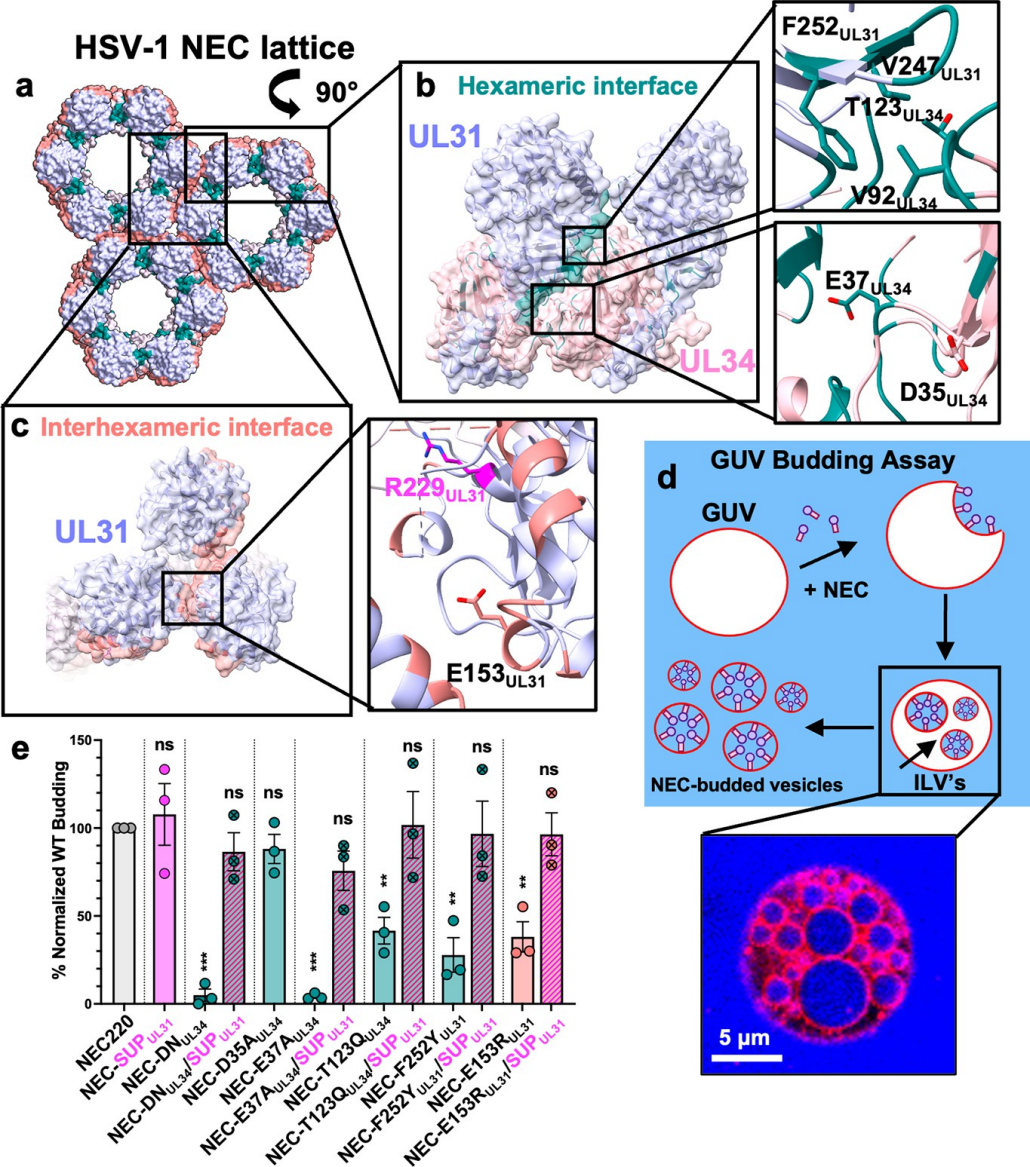
The SUP_UL31_ mutation restores budding activity to budding-deficient oligomeric interface NEC mutants. **a-c)** HSV-1 NEC hexameric and interhexameric interfaces highlighting the locations of residues mutated for this study. **d)** A cartoon representation of the GUV budding assay showing the NEC (purple circles and pink rectangles) binding to red fluorescent GUVs and undergoing negative curvature to form an NEC-coated intraluminal vesicle (ILV). Free NEC continues to bud the GUVs until only fully budded vesicles containing NEC on the interior remain. Cascade blue, a membrane impermeant dye, is used to monitor budding. **e)** SUP-R229L_UL31_ rescues budding in both hexameric and interhexameric budding-deficient NEC mutants *in vitro*. The percentage of *in vitro* budding was determined by counting the number of ILVs within the GUVs after the addition of NEC220 or the corresponding NEC mutant. A background count, the number of ILVs in the absence of NEC, was subtracted from each condition. Each construct was tested in three biological replicates, each consisting of three technical replicates. Symbols show the average budding efficiency of three technical replicates compared to NEC220 (100%; grey). Significance was calculated using an unpaired Student’s t-test with Welch’s correction (P < 0.01 = **; P < 0.001 = ***; ns = not significant) in GraphPad Prism 9.0.

HSV-1 NEC oligomerizes into a hexagonal lattice (**Fig 1A**) stabilized by interactions between NEC heterodimers within hexamers (hexameric interface; **Fig 1B**) and between hexamers (interhexameric interface; **Fig 1C**). NEC hexameric lattice formation is essential for membrane budding because mutations engineered to disrupt lattice interfaces reduce budding *in vitro* [19,24] and nuclear egress in infected cells [26,27]. These budding-deficient mutations are D35A_UL34_/E37A_UL34_ (DN_UL34_), V92F_UL34_, T123Q_UL34_, V247F_UL31_, and F252Y_UL31_ at the hexameric interface (**Fig 1B**) and E153R_UL31_ at the interhexameric interface **(Fig 1C)**. The SUP_UL31_ mutation restores budding *in vitro* to DN_UL34_ and V92F_UL34_ mutants [24]. Here, we asked if it could restore budding to other interface mutants, T123Q_UL34_ and F252Y_UL31_ (hexameric) **(Fig 1B)** and E153R_UL31_ (interhexameric) **(Fig 1C)**.

We also wanted to assess the individual contributions of D35A_UL34_ and E37A_UL34_ mutations to the budding-deficient phenotype of the DN_UL34_ mutant. Residue E37_UL34_ is located at the hexameric interface where its side chain forms a hydrogen bond with T89_UL31_ of the neighboring NEC heterodimer. The E37A_UL34_ mutation eliminates this hydrogen bond, which would disrupt the hexameric interface. Indeed, the E37A_UL34_ mutant was deficient in budding *in vitro* [24]. However, the side chain of residue D35_UL34_ points away from the hexameric interface (**Fig 1B**). Therefore, we tested if the D35A_UL34_ mutation would have any effect on budding.

The *in-vitro* budding activity of all NEC mutants was measured by an established assay [19,24,31,32] utilizing fluorescently labeled giant unilamellar vesicles (GUV), soluble fluorescent dye Cascade Blue, and the soluble version of HSV-1 NEC, NEC220, which contained full-length UL31 and UL34 residues 1–220 **(Fig 1D)**. We first confirmed the *in-vitro* phenotypes of the budding-deficient mutants. Both DN_UL34_ and E37A_UL34_ mutations reduced budding to ~10% of the WT NEC220 **(Fig 1E),** consistent with our previous findings [24] whereas the D35A_UL34_ mutation alone had no effect **(Fig 1E).** Thus, the E37A_UL34_ mutation is solely responsible for the nonbudding phenotype of DN_UL34_. The interface mutations T123Q_UL34_, F252Y_UL31_, and E153R_UL31_ (**Fig 1BC**) reduced budding to ~30–40% of the WT NEC220 **(Fig 1E)**, as previously observed [24]. The SUP_UL31_ mutation did not affect the budding efficiency of the WT NEC220 but restored budding not only to the DN_UL34_ as shown previously [24] but to all other lattice interface mutants regardless of their location **(Fig 1E)**. Thus, the SUP_UL31_ mutation can restore efficient budding to a broad range of lattice interface mutants.

### The SUP_UL31_ mutation complements the growth defects of HSV-1 containing oligomeric interface mutations

**Fig 2:**
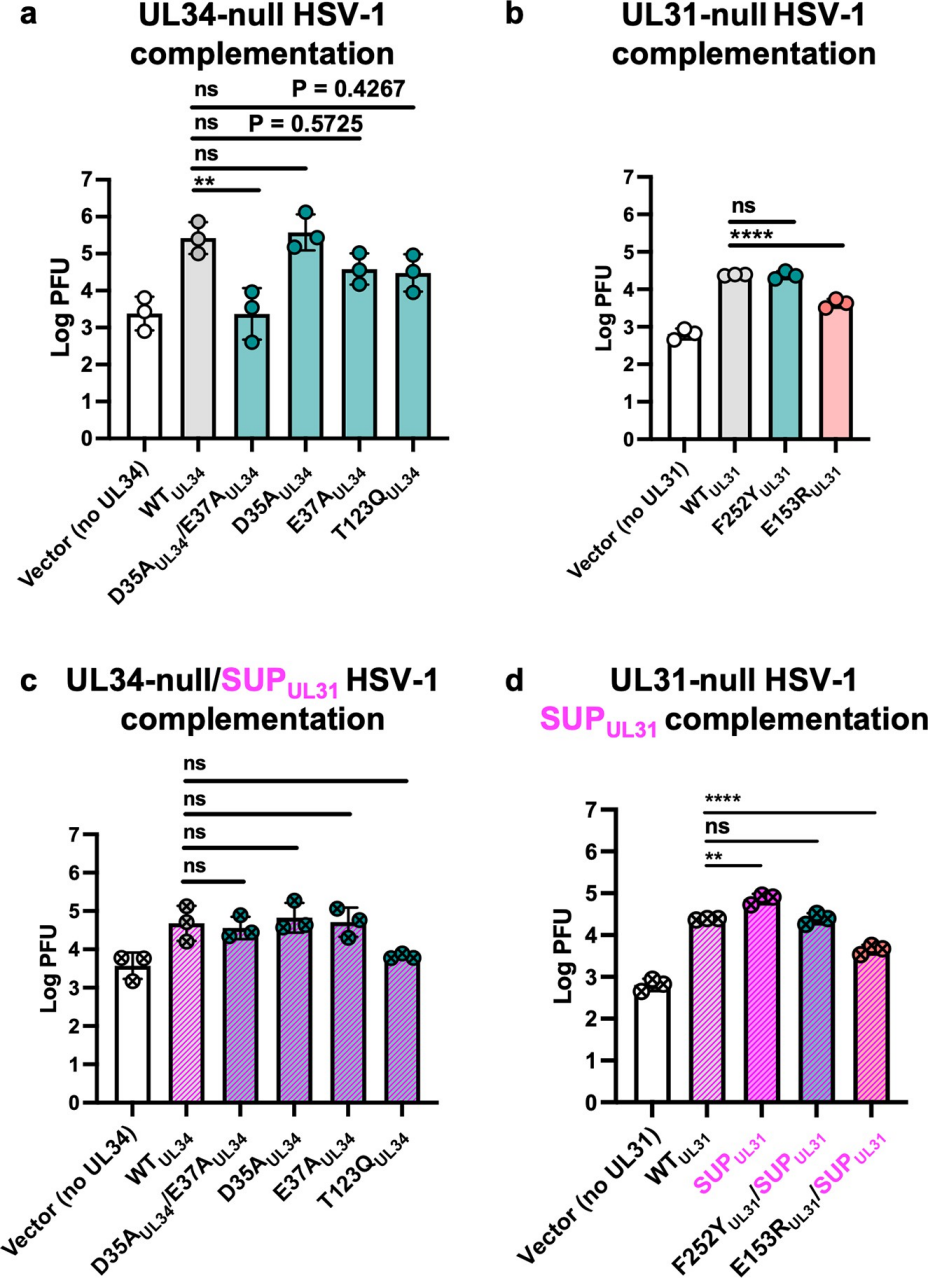
The SUP_UL31_ mutation complements the growth defects of HSV-1 containing oligomeric interface mutations. **(a-b)** WT_UL31_ can only complement the growth of the D35A_UL34_ and F252Y_UL31_ oligomeric interface mutants whereas SUP_UL31_ can complement more **(c-d).** For all experiments, Hep-2 cells were transfected with the corresponding UL34 or UL31 mutant plasmid (x-axis labels) and infected with either a UL34-null **(a)**, a UL31-null virus **(b)**, a UL34-null/UL31_R229L_ virus **(c)**, or a UL31-null virus with the SUP_UL31_ mutation added in *trans* **(d**). Each bar represents the mean of three independent experiments. Statistical significance was determined by performing a one-way ANOVA on log-converted values using the Method of Tukey for multiple comparisons of each mutant against the WT in GraphPad Prism. P < 0.01 = **; P < 0.0001 = ****; ns, not significant.

To correlate the *in-vitro* budding phenotypes with the infected cell phenotypes, we used an established viral growth complementation assay [11]. This assay measures the ability of a mutant protein expressed *in trans* to complement the poor growth of a virus lacking the corresponding gene (the so-called null virus). Hep-2 cells were transfected with plasmids encoding either WT (UL31 or UL34), mutant UL34 (D35A_UL34_/E37A_UL34_, D35A_UL34_, E37A_UL34_, T123Q_UL34_), or mutant UL31 (F252Y_UL31_ and E153R_UL31_) and then infected with either a UL34-null HSV-1 or UL31-null HSV-1. The amount of infectious viral progeny produced was measured by plaque assay on either UL34-expressing **(Fig 2A)** or UL31-expressing Vero cells **(Fig 2B).** We found that cells expressing the D35A_UL34_/E37A_UL34_, E37A_UL34_, T123Q_UL34_, or E153R_UL31_ mutants did not trans-complement replication of either the UL34-null **(Fig 2A)** or UL31-null HSV-1 **(Fig 2B)**, as efficiently as the WT UL34 or WT UL31, respectively. This is in agreement with their impaired budding phenotypes *in vitro*. Although not statistically significant, the reduced trans-complementation ability of E37A_UL34_ and T123Q_UL34_ was evident. By contrast, the D35A_UL34_ mutant trans-complemented UL34-null HSV-1 with WT UL34 efficiency **(Fig 2A)**. Surprisingly, the F252Y_UL31_ mutant trans-complemented UL31-null HSV-1 with the WT UL31 efficiency despite reduced budding in our *in-vitro* budding assay **(Fig 1E)**.

To rule out increased protein expression levels as the trans-complementation mechanism, we measured expression levels of transfected WT UL34, WT UL31, and the corresponding mutant proteins during infection with the corresponding null virus. All mutant UL34 proteins expressed at WT UL34 levels **(S1A Fig)**. Reduced complementation efficiencies of D35A_UL34_/E37A_UL34_, E37A_UL34_, and T123Q_UL34_ (**Fig 2A**) are thus due to the specific mutation(s). In contrast, both F252Y_UL31_ and E153R_UL31_ are overexpressed relative to the WT UL31 and R229L_UL31_ **(S1B Fig)**. The surprisingly efficient complementation by the F252Y_UL31_ mutant may therefore be due to its higher expression levels **(Fig 2B)**. We have not, however, generated a recombinant virus expressing F252Y_UL31_ to assess its function at physiological expression levels to confirm this.

Next, we tested the ability of the SUP_UL31_ mutation to restore efficient complementation to the lattice interface mutants, i.e., to suppress their complementation defects. To test the UL34 mutants, we generated the UL34-null/SUP_UL31_ HSV-1 virus. Hep-2 cells were transfected with plasmids encoding either WT UL34 or mutant UL34 (D35A_UL34_/E37A_UL34_, D35A_UL34_, E37A_UL34_, or T123Q_UL34_) and then infected with a UL34-null/SUP_UL31_ HSV-1. UL34-null/SUP_UL31_ HSV-1 yields ~1-log-fold lower viral titer when complemented with WT UL34 **(Fig 2C)** than the UL34-null HSV-1 complemented with WT UL34 **(Fig 2A)**, as reported previously [27]. Therefore, complementation of the UL34-null/SUP_UL31_ virus by the WT UL34 was used as a reference point for assessing the ability of the SUP_UL31_ mutation to restore efficient complementation to UL34 mutants. Indeed, the SUP_UL31_ mutation rescued complementation defects of both the D35A_UL34_/E37A_UL34_ and E37A_UL34_ mutants **(Fig 2C)**, both of which poorly complemented the growth of the UL34-null HSV-1 **(Fig 2A)**. The SUP_UL31_ mutation had no obvious effect on the already efficient complementation by the D35A_UL34_ **(Fig 2C).** Unexpectedly, it did not fully restore the poor complementation by the T123Q_UL34_ **(Fig 2C)** despite restoring its budding defect *in vitro* **(Fig 1E).** We note, however, that the difference in complementation levels between the T123Q_UL34_ mutant and the WT UL34 are not statistically significant **(Fig 2C)**.

To test the UL31 mutants, we generated double UL31 mutant plasmids harboring the UL31 mutation of interest and the SUP_UL31_ mutation. Hep-2 cells were transfected with plasmids encoding either WT UL31 or mutant UL31 (R229L_UL31_, F252Y_UL31_/R229L_UL31_, or E153R_UL31_/R229L_UL31_, and then infected with the UL31-null HSV-1. Complementation of UL31-null viruses by the WT UL31 was used as a reference point for assessing the ability of the SUP_UL31_ mutation to restore efficient complementation to UL31 mutants. The SUP_UL31_ mutation complemented the growth defect of the UL31-null HSV-1 somewhat more efficiently than the WT UL31. We also occasionally observe more efficient budding by the NEC-SUP_UL31_ in our *in vitro* budding assays [24] but the reason for this is unclear. In HSV-1 UL31-null infected cells, the SUP_UL31_ mutation had no obvious effect on the already efficient complementation by the F252Y_UL31_ mutant **(Fig 2D).** However, it was unable to fully restore the poor complementation by the E153R_UL31_ mutant **(Fig 2D)** despite restoring its budding defect *in vitro* **(Fig 1E).**

The reason for poor complementation of the T123Q_UL34_ and E153R_UL31_ mutations by the SUP_UL31_ mutation is yet unclear. We hypothesize that these mutations may impair some other important viral replication function of UL34 or UL31, respectively, that cannot be suppressed by the SUP_UL31_ mutation, e.g., nuclear lamina dissolution, capsid docking at the INM, or capsid recruitment.

### The SUP_UL31_ mutation restores efficient budding *in vitro* to heterodimeric interface mutants and complements their viral growth defects

**Fig 3:**
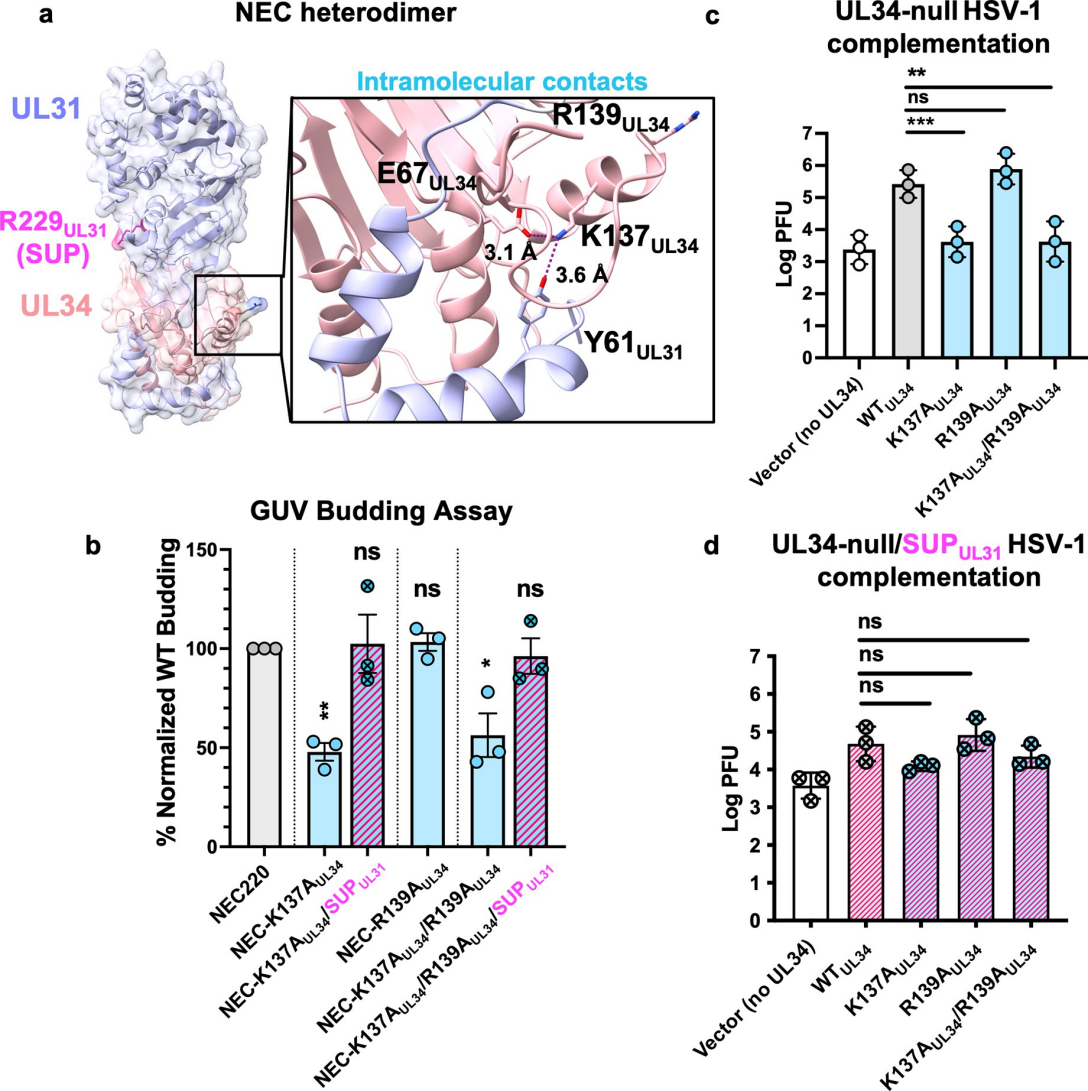
The SUP_UL31_ mutation restores budding to heterodimeric interface mutants and complements viral growth defects. **a)** Locations of intramolecular NEC residues mutated for this study. Inset shows interactions between various residues at the heterodimeric interface thought to be important for NEC heterodimer stabilization. **b)** NEC-SUP_UL31_ rescues budding of NEC heterodimeric interface mutants *in vitro*. The percentage of *in-vitro* budding was determined by counting the number of ILVs within the GUVs after the addition of NEC220 or the corresponding NEC mutant. A background count, the number of ILVs in the absence of NEC, was subtracted from each condition. Each construct was tested in three biological replicates, each consisting of three technical replicates. Symbols show the average budding efficiency of three technical replicates compared to NEC220 (100%; grey). Significance was calculated using an unpaired Student’s t-test with Welch’s correction (P < 0.05 = *; P < 0.01 = **; ns = not significant) in GraphPad Prism 9.0. **c)** WT_UL34_ can only complement the growth of the R139A_UL34_ heterodimeric interface mutant whereas SUP_UL31_ **(d)** can partially complement more. For both experiments, Hep-2 cells were transfected with the corresponding UL34 mutant plasmid and infected with a UL34-null virus **(c)** or a UL34-null/UL31_R229L_ virus **(d)**. Each bar represents the mean of three independent experiments. Statistical significance was determined by performing a one-way ANOVA on log-converted values using the Method of Tukey for multiple comparisons implemented on GraphPad Prism. **, P < 0.01 = **; P < 0.001 = ***; ns, not significant.

The aforementioned mutational screen targeting charge clusters in the HSV-1 UL34 sequence [28], identified another double mutant, K137A_UL34_/R139A_UL34_, that could not trans-complement the growth of the HSV-1 UL34-null virus. This suggested that residues K137_UL34_ and R139_UL34_ are important for HSV-1 replication. The double mutation did not affect the NEC localization to the INM, suggesting a defect in the NEC function [28]. In the HSV-1 NEC crystal structure, K137_UL34_ forms salt bridges with E67_UL34_ and Y61_UL31_ at the heterodimeric interface between the globular domains of UL31 and UL34 **(Fig 3A, inset)**. Thus, K137_UL34_ could contribute to the stabilization of the NEC heterodimer. By contrast, R139_UL34_ does not form any obvious interactions **(Fig 3A, inset)**.

To test the effect of the K137A_UL34_, R139A_UL34_, and K137A_UL34_/R139A_UL34_ mutations on the heterodimer stability and budding activity *in vitro*, we introduced them into the recombinant NEC220. Typically, size-exclusion chromatography on samples of purified, WT NEC220 yields only fractions containing equimolar amounts of UL31 and UL34, indicating the intact UL31:UL34 = 1:1 complex [19]. Indeed, this pattern was observed for the NEC220-R139A_UL34_ mutant **(S2A Fig).** However, both NEC220-K137A_UL34_ and NEC220-K137A_UL34_/R139A_UL34_ mutants also yielded fractions containing free UL34 or fractions containing more UL34 than UL31 **(S2B and S2C Fig)** despite equimolar amounts of UL31 and UL34 being loaded onto the size-exclusion column. Thus, the K137A_UL34_ mutation appeared to destabilize the NEC heterodimer. No free UL31 was detected in any of the fractions, suggesting that it may have been retained on a filter within the chromatography line. Only fractions containing equimolar amounts of UL31 and UL34 were used for further characterization.

Both K137A_UL34_ and K137A_UL34_/R139A_UL34_ mutations reduced budding to ~50% of the WT NEC220 whereas the R139A_UL34_ mutation had no effect **(Fig 3B)**. The K137A_UL34_ mutation is thus solely responsible for the defective budding phenotype of the double K137A_UL34_/R139A_UL34_ mutant. Surprisingly, the SUP_UL31_ mutation fully restored efficient budding to both K137A_UL34_ and K137A_UL34_/R139A_UL34_ mutants **(Fig 3B).** But the mutant NEC heterodimers still eluted as fractions containing either free UL34 or mutant NEC complex, suggesting the mutant NEC complexes remained unstable despite the presence of the SUP_UL31_ mutation **(S3 Fig)**. Therefore, the SUP_UL31_ mutation does not restore budding by restoring heterodimer stability.

To assess the effects of these mutations on viral replication, we performed the viral growth complementation assay described above for UL34 mutants. Both K137A_UL34_ and R139A_UL34_ proteins were expressed at WT UL34 levels (**S1A Fig**). R139A_UL34_ complemented the growth of both the UL34-null **(Fig 3C)** and UL34-null/SUP_UL31_ viruses on par with the WT UL34 **(Fig 3D).** As expected, K137A_UL34_ and K137A_UL34_/R139A_UL34_ complemented growth of the UL34-null virus poorly **(Fig 3C),** which is consistent with their *in-vitro* budding defects. However, both mutants complemented the growth of the UL34-null/SUP_UL31_ virus almost as efficiently as the WT UL34 **(Fig 3D)**. Therefore, SUP_UL31_ mutation can restore both budding and replication defects caused by the K137A_UL34_ mutation.

### The SUP_UL31_ mutation partially restores budding *in vitro* to a membrane interface mutant

**Fig 4:**
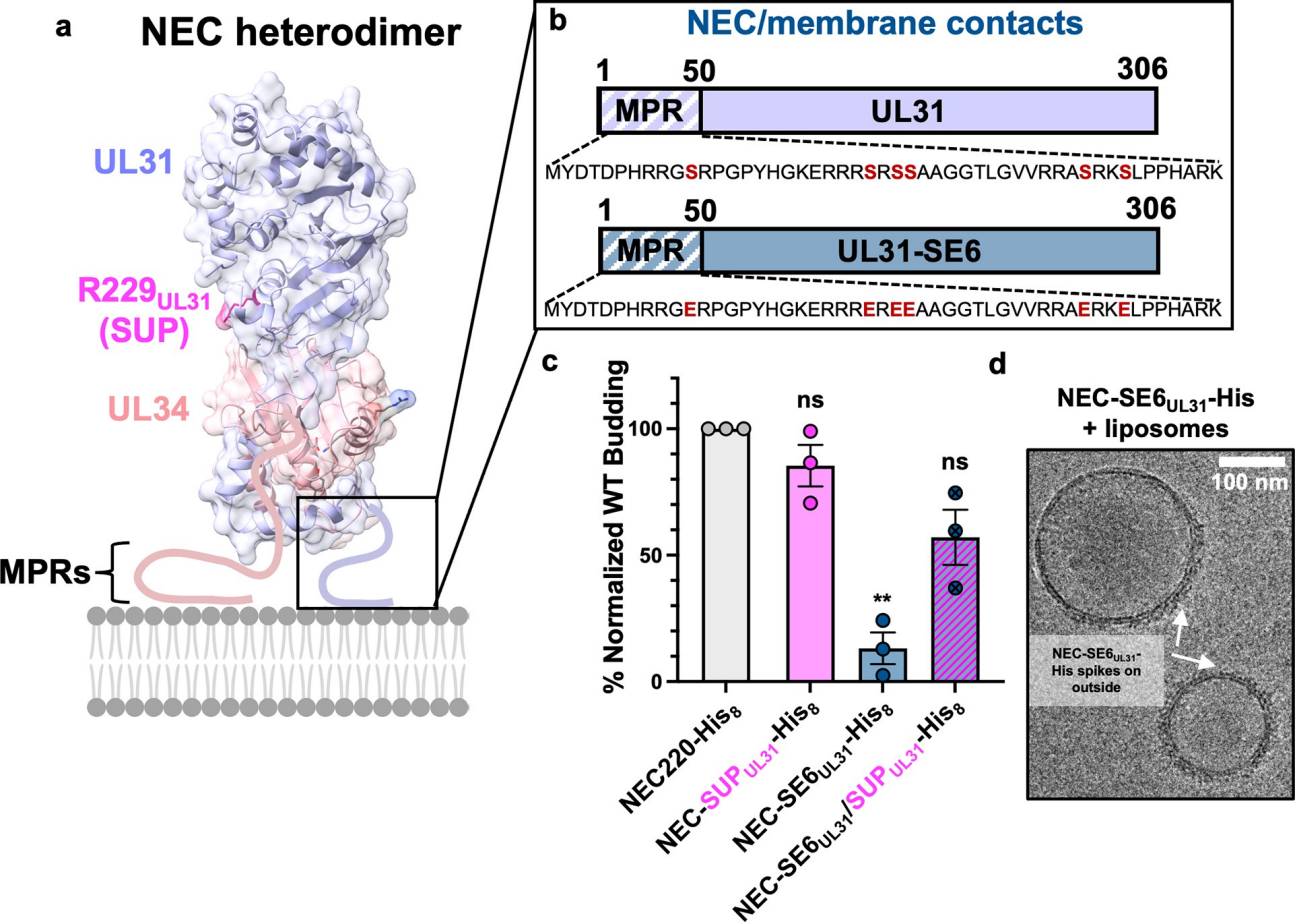
The SUP_UL31_ mutation partially restores budding to a membrane interface mutant. **a-b)** Location of membrane interface residues mutated for this study. **c)** NEC-SUP_UL31_ partially rescues budding in the membrane interface mutant *in vitro*. The percentage of *in-vitro* budding was determined by counting the number of ILVs within the GUVs after the addition of NEC220-His or the corresponding NEC mutant. A background count, the number of ILVs in the absence of NEC, was subtracted from each condition. Each construct was tested in three biological replicates, each consisting of three technical replicates. Symbols show the average budding efficiency of three technical replicates compared to NEC220-His_8_ (100%; grey). The NEC-SE6_UL31_-His_8_ data were previously reported in [31]. Significance was calculated using an unpaired Student’s t-test with Welch’s correction (P < 0.01 = **; ns = not significant) in GraphPad Prism 9.0. **d)** CryoEM of NEC-SE6_UL31_-His_8_ and large unilamellar vesicles (LUVs) shows that the SE6_UL31_ mutations perturb NEC oligomerization when bound to membranes.

In addition to UL31/UL34 and NEC/NEC interfaces, the NEC/membrane interface is also functionally important in HSV-1 NEC. Both UL31 and UL34 contain membrane-proximal regions (MPRs) **(Fig 4A and 4B)** that mediate membrane association [19,31] and are essential for budding *in vitro* [31]. The UL31 MPR contains clusters of positively charged residues that interact with model membranes and facilitate membrane deformation and budding by peripherally inserting into the membrane and increasing lipid order [31]. The UL31 MPR also contains six serines **(Fig 4B)** that are phosphorylated during infection [7] by the HSV-1 kinase US3 [33]. Phosphomimicking serine-to-glutamate mutations of these six serines (SE6_UL31_) **(Fig 4B)** reduce nuclear egress and viral titers during HSV-1 infection [34] and impair NEC/membrane interactions and budding activity *in vitro* [31]. Previously, we proposed that negative charges introduced by phosphorylation or phosphomimicking mutations reduce electrostatic interactions between the MPR and the lipid headgroups that are necessary for membrane deformation and budding [31]. Here, we asked whether the SUP_UL31_ mutation could restore budding *in vitro* to the budding deficient SE6_UL31_ mutant.

The NEC220 construct typically used in the *in-vitro* budding assays is soluble and depends on functional MPRs for membrane recruitment. Since the SE6_UL31_ mutation reduces NEC/membrane interactions, to bypass the defect in membrane recruitment, we used the NEC220 variant construct that contains a His_8_-tag at the C terminus of UL34 [19,31]. When used in conjunction with membranes containing Ni-chelating lipids, the His_8_-tag ensures that the NEC220-His_8_ is recruited to the membranes even when the MPR mutations preclude membrane association. The *in-vitro* budding efficiency of NEC220-His_8_ was similar to that of untagged NEC220, suggesting that the C-terminal His_8_-tag has no deleterious effect on the membrane budding activity [19,31]. Previously, we showed that the NEC constructs that lack the MPRs do not bud membranes even when tethered to Ni-containing liposomes by His_8_-tag because such a tag cannot restore the ability to order lipids [31].

By itself, the SUP_UL31_ mutation did not change the budding efficiency of NEC220-His_8_ **(Fig 4C)**, similar to the untagged NEC220 **(Fig 1E).** As previously reported by our group, SE6_UL31_ mutation reduced *in-vitro* budding to ~10% of the WT NEC220-His_8_ despite the ability to interact with membranes due to the His_8_-tag [31] **(Fig 4C)**. Therefore, we performed a cryoEM analysis to examine NEC220-SE6_UL31_-His_8_ membrane interactions. NEC220-SE6_UL31_-His_8_ was incubated with large unilamellar vesicles (LUVs) of similar composition to the GUVs used for the budding assay and imaged with cryoEM **(Fig 4D)**. NEC220-SE6_UL31_-His_8_ formed membrane-bound spikes on the outside of the LUVs (**Fig 4D)**, but the internal protein coats indicative of budding [19,21,32] were rarely observed. This is reminiscent of the behavior of the oligomerization-deficient NEC-DN_UL34_ mutant previously reported by our group [19]. We conclude that the SE6_UL31_ mutations perturb NEC oligomerization, likely as the consequence of weakened MPR/membrane interactions [31]. Surprisingly, the SUP_UL31_ mutation restored budding of the SE6_UL31_ mutant to ~50% of the WT NEC220-His_8_ **(Fig 4C).** Therefore, the SUP_UL31_ mutation can rescue budding *in vitro*, even if partially, to a mutant that indirectly disrupts oligomerization by weakening MPR/membrane interactions.

### WT NEC, NEC-SUP_UL31_, and NEC-DN_UL34_/SUP_UL31_ form similar hexagonal lattices on vesicles budded *in vitro*

To identify the mechanism by which the SUP_UL31_ mutation can restore budding to a broad range of mutants, we examined its effect on the NEC lattice. As the SUP_UL31_ mutation rescues budding defects caused by mutations that disrupt hexagonal lattice interfaces, it would be expected to reinforce the weakened hexagonal lattice. Yet, residue R229_UL31_ is far away from the hexameric interface and while near the interhexameric interface, it does not make any contacts there **(Fig 1C)**. Therefore, we initially hypothesized that the SUP_UL31_ mutation promoted the formation of an NEC lattice with an alternative geometry.

**Fig 5:**
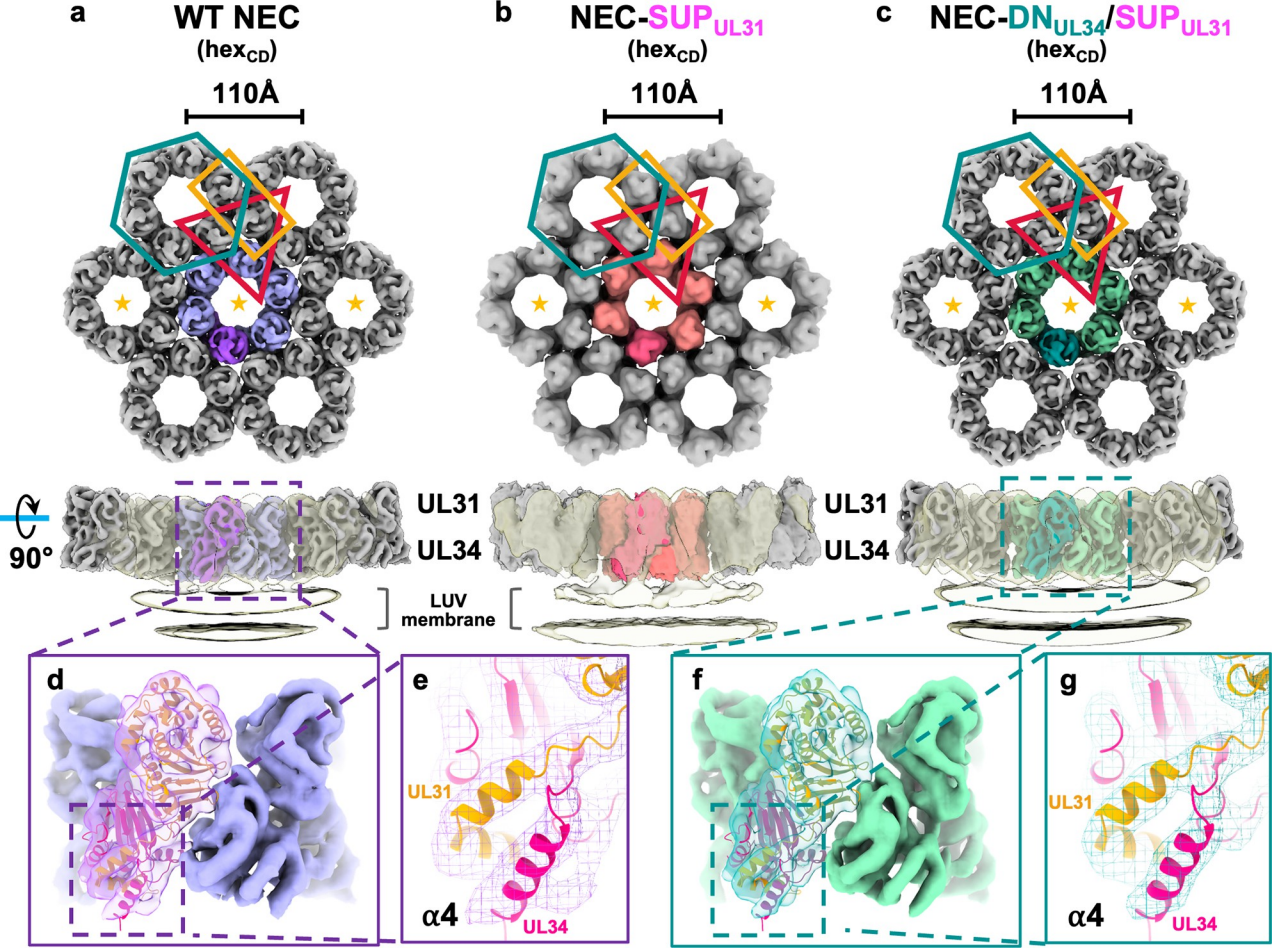
WT NEC, NEC-SUP_UL31_, and NEC-DN_UL34_/SUP_UL31_ form similar membrane-bound hexagonal coats. CryoET reconstruction of **a)** the WT NEC coat at 5.9 Å, **b)** the NEC-SUP_UL31_ coat at 13.1 Å, and **c)** the NEC-DN_UL34_/SUP_UL31_ coat at 5.4 Å. Only the three hexameric units marked with orange stars are shown in lower 90°-rotated panels, where the low-pass filtered transparent densities show the connection between NEC lattices and LUV membrane. The HSV-1 NEC crystal structure (PDB ID: 4ZXS) docks similarly into both the WT NEC **(d)** and NEC-DN_UL34_/SUP_UL31_ **(f)** cryoET densities. **e, g)** Docking of the ⍺4 helix from the PRV NEC crystal structure (PDB ID: 4Z3U) accounts for the additional density observed in both cryoET reconstructions which was originally unresolved in the HSV-1 NEC crystal structure [24]. All coats have the hex_CD_ arrangement.

To examine the effect of the SUP_UL31_ mutation on the geometry of the membrane-bound NEC coats, we performed cryoEM/T analyses on WT NEC220 and the mutants NEC220-SUP_UL31_ and NEC220-DN_UL34_/SUP_UL31_. Each protein complex was incubated with LUVs similar in composition to those used in our prior cryoET reconstructions of the WT NEC220 coats [19,21,32]. Using sub-tomographic averaging, we obtained the 3D reconstructions of the WT NEC220 (5.9 Å), NEC220-SUP_UL31_ (13.1 Å), and NEC220-DN_UL34_/SUP_UL31_ (5.4 Å) **(Fig 5)**. The lower final resolution of the NEC220-SUP_UL31_ reconstruction was due to the aggregation of budded vesicles formed by NEC220-SUP_UL31_, which reduced the number of NEC220-SUP_UL31_ particles available for data processing. The higher resolution of the WT NEC220 and NEC220-DN_UL34_/SUP_UL31_ averaged cryoET density map allowed us to dock the crystal structure of the WT NEC185Δ50 heterodimer **(Fig 5D and 5F),** confirming that the SUP_UL31_ mutation does not perturb NEC conformation in a major way, even when bound to membranes. We also observed additional helical density at the C terminus of UL34, which corresponded to helix ⍺4 that was unresolved in the WT NEC185Δ50 crystal structure [24] yet present in the crystal structures of NEC homologs from PRV [24,35], HCMV [25,36], and EBV [21]. The PRV UL34 ⍺4 helix fit well into the HSV-1 UL34 cryoET averages **(Fig 5E and 5G)**.

All three constructs formed very similar hexagonal lattices on budded membranes **(Fig 5A–5C and S1 Table)**, showing that the SUP_UL31_ mutation did not promote the formation of an NEC lattice with an alternative geometry. Based on the cryoET data, we hypothesized that the SUP_UL31_ mutation reinforced the hexagonal lattice.

### WT NEC, NEC-SUP_UL31_, and NEC-DN_UL34_/SUP_UL31_ form similar hexagonal lattices in the crystals

To obtain a high-resolution view of the hexagonal lattice formed by the NEC-SUP_UL31_ mutant, we crystallized the NEC185Δ50-SUP_UL31_ and the NEC185Δ50-DN_UL34_/SUP_UL31_ constructs, the mutant equivalents of the previously crystallized WT NEC185Δ50 construct (UL31: 51–306 and UL34: 15–185) [24]. Both mutants took the space group C2_1_ with six crystallographically independent NEC heterodimers in the asymmetric unit, SUP_AB_, SUP_CD_, SUP_EF_, SUP_GH_, SUP_IJ_, and SUP_KL_ (UL34 chains: A, C, E, G, I, and K; UL31 chains: B, D, F, H, J, and L) **(S2 Table).** Phases were determined using molecular replacement with the WT NEC185Δ50 structure as a search model. The NEC185Δ50-SUP_UL31_ structure was refined to 3.9-Å resolution **(S2 Table)**. The atomic coordinates and structure factors of the NEC185Δ50-SUP_UL31_ structure were deposited to the RCSB Protein Data Bank under the accession number 8G6D. However, the NEC185Δ50-DN_UL34_/SUP_UL31_ crystals diffracted x-rays only to ~6 Å resolution, which precluded atomic refinement **(S2 Table)**. Therefore, the NEC185Δ50-SUP_UL31_ structure was used for the in-depth analysis of the interfaces and conformational changes whereas the structural analysis of the NEC185Δ50-DN_UL34_/SUP_UL31_ structure was limited to the analysis of crystal packing.

**Fig 6:**
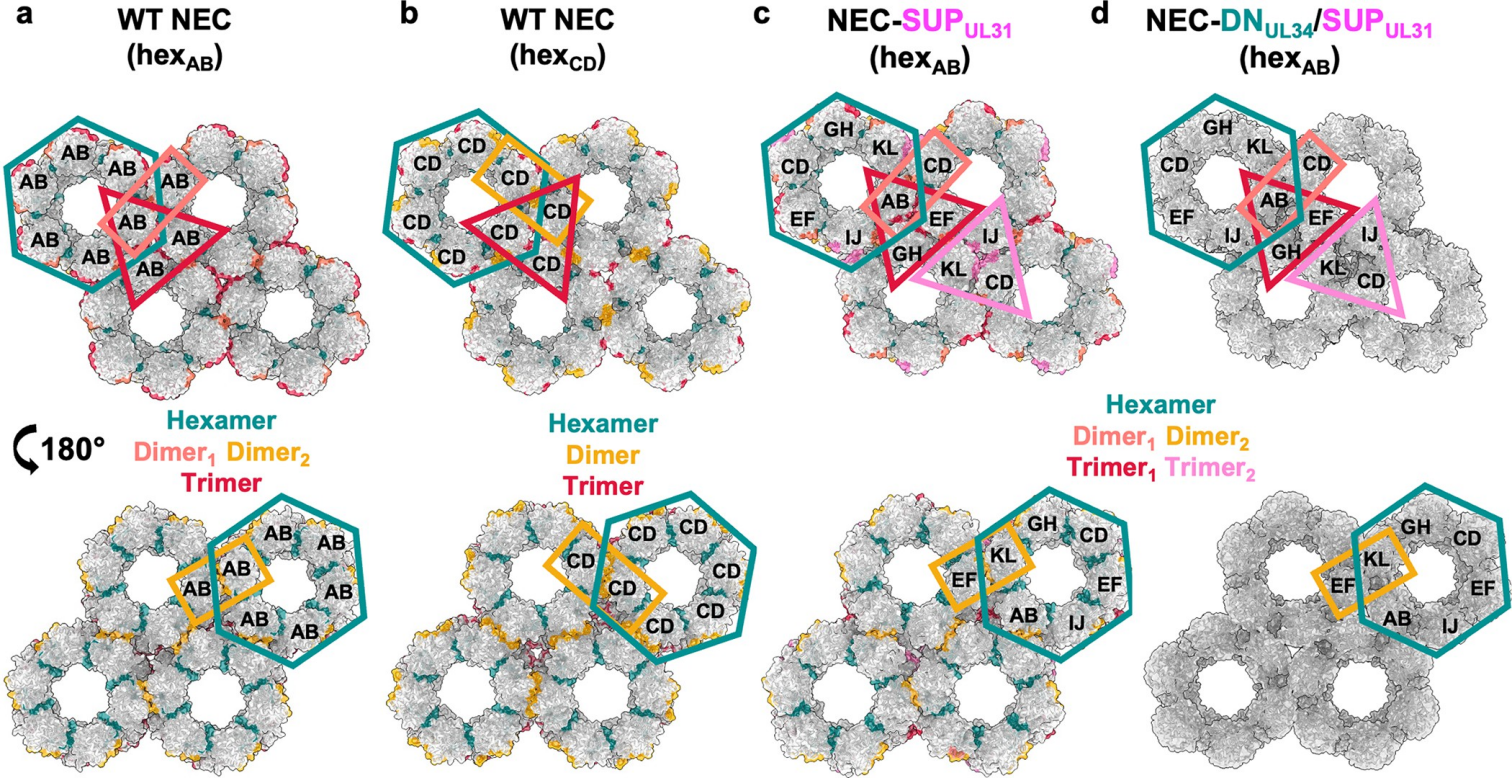
NEC-SUP_UL31_ and NEC-DN_UL34_/SUP_UL31_ form hex_AB_ lattices in the crystals. **a)** The HSV-1 WT NEC hex_AB_ (PDB ID: 4ZXS)_,_ **b)** the WT NEC hex_CD_ (PDB ID: 4ZXS), **c)** the NEC-SUP_UL31_ and **d)** the NEC-DN_UL34_/SUP_UL31_ crystal lattices [24]. Hexameric (teal) and interhexameric (dimer_1_: coral, dimer_2_: gold, trimer_1_: red, and trimer_2_: light pink) interfaces are colored accordingly. Two distinct trimers formed in the NEC-SUP_UL31_ lattice (red and light pink). Due to resolution, an interface analysis was not performed on the NEC-DN_UL34_/SUP_UL31_ crystal lattice. The corresponding heterodimers within the lattice are labeled.

The previously crystallized HSV-1 WT NEC185Δ50 construct took the space group P6 with two crystallographically independent NEC heterodimers in the asymmetric unit, NEC_AB_ and NEC_CD_ (UL34 chains: A and C; UL31 chains: B and D) [24]. While the two heterodimers formed very similar, perfectly symmetrical hexamers, they arranged into two distinct hexagonal lattices, hex_AB_ and hex_CD_ **(Fig 6A and 6B and S3 Table)** [24]. In both lattices, hexamer interactions result in trimers formed by UL31/UL31 interactions **(Fig 6A and 6B, red).** However, the hex_AB_ lattice also has two types of dimers formed by either UL31/UL31 or UL31/UL34 interactions **(Fig 6A, coral and gold,** respectively) whereas the hex_CD_ lattice has only one dimer type formed by UL31/UL34 interactions **(Fig 6B, gold).**

Just as the WT NEC185Δ50, the NEC185Δ50-SUP_UL31_ formed hexamers in the crystals. Only in this case, the hexamers were not perfectly symmetrical, being formed by six independent, non-crystallographic heterodimers in the asymmetric unit **(Fig 6C).** Nonetheless, the NEC185Δ50-SUP_UL31_ hexamers looked very similar to the WT NEC185Δ50 hexamers, with similar hexameric interfaces **(S3 and S4 Tables)**. The crystal lattice formed by the NEC185Δ50-SUP_UL31_ hexamers **(Fig 6C)** resembled the WT NEC hex_AB_ lattice **(Fig 6A and S5–S7 Tables).** The NEC185Δ50-DN_34_/SUP_31_ crystal lattice **(Fig 6D)** also resembled the WT NEC hex_AB_ lattice **(Fig 6A).** The SUP_UL31_ mutation thus had no major effect on the hexamer structure or the hexagonal lattice.

Interestingly, while the WT NEC formed both hex_AB_ and hex_CD_ lattices in the crystals and the mutants formed only the hex_AB_ lattice **(Fig 6)**, in the membrane-bound coats, the WT NEC220 and both mutants formed the hex_CD_ lattice **(Fig 5).** The reasons for these differences are yet unclear. Regardless, just as the WT NEC, the mutants could form both types of the hexagonal lattice.

### The SUP_UL31_ mutation eliminates a salt bridge and changes loop conformations

**Fig 7:**
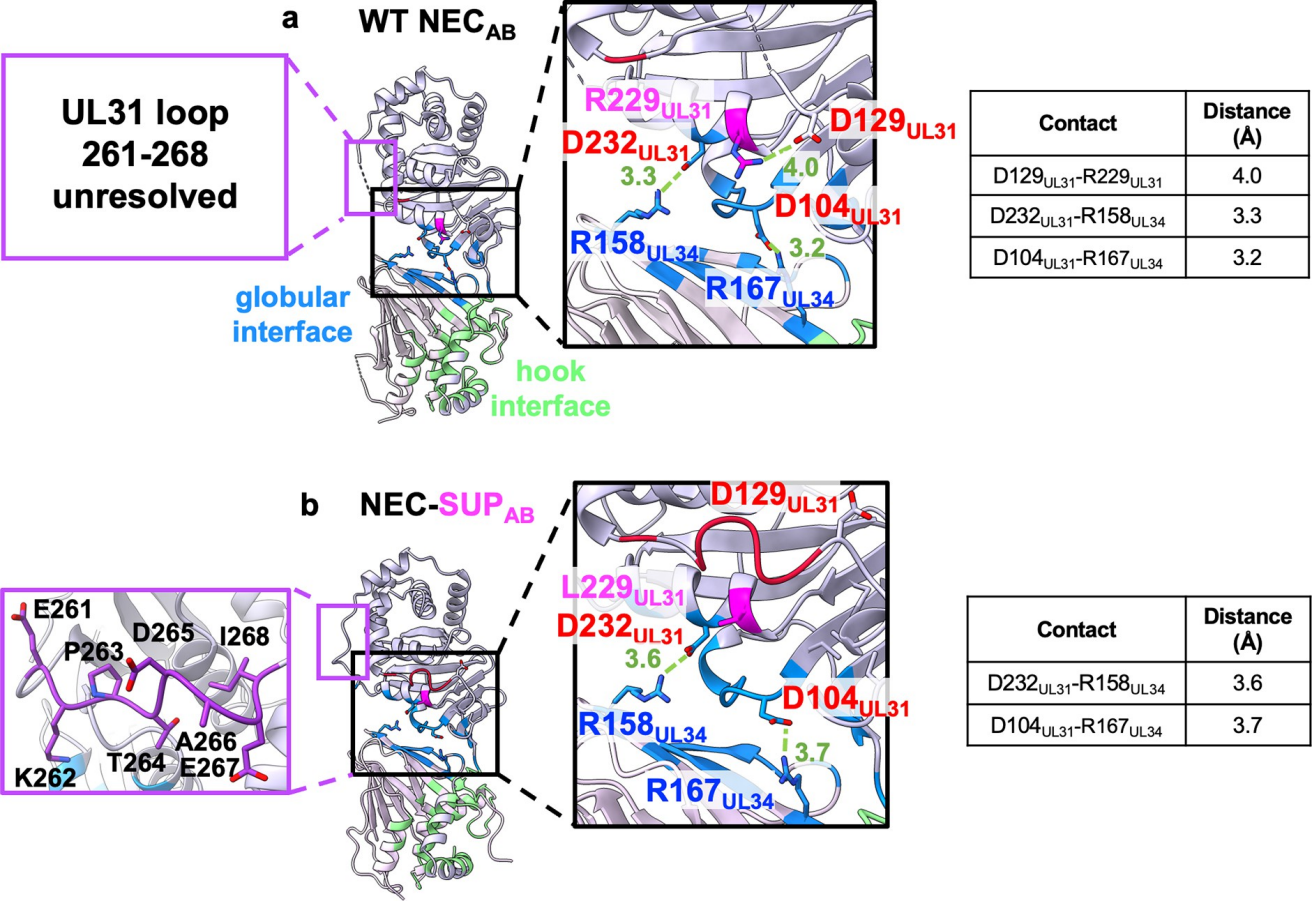
The SUP_UL31_ mutation eliminates a salt bridge near the heterodimeric interface and changes loop conformations. The crystal structures of **(a)** WT NEC_AB_ and **(b)** NEC-SUP_AB_ heterodimers. The heterodimeric interface residues are colored blue (globular interface) or green (hook interface). Residue 229_UL31_ is colored magenta. Insets show close-up views of the globular heterodimeric interface. Residues forming salt bridges at the interface are shown as sticks and colored in blue (Rs) or red (Es and Ds). Residue 229_UL31_ is shown as sticks and colored in magenta. Salt bridges are shown as dashed green lines, with distances in Angstrom. Distances are also listed in the corresponding tables. The resolved portions of the dynamic loops 129_UL31_-134_UL31_ and 261_UL31_-268_UL31_ are shown in red and purple, respectively. The HSV-1 NEC crystal structure (PDB: 4ZXS) was used to generate the figure in (**a)**.

To identify any local conformational changes in the mutant NEC lattices, we compared NEC185Δ50-SUP_UL31_ heterodimers to each other and to the WT NEC185Δ50 heterodimers **(Fig 7)**. The six crystallographically independent heterodimers in the NEC185Δ50-SUP_UL31_ structure were structurally similar to each other **(S4 Fig and S8 and S9 Tables)** and to the two WT NEC heterodimers [24] **(Figs 7 and S4 and S10 Table)** and could be superimposed with RMSDs ranging from 0.67 to 1.02 Å **(S11 Table).** Thus, the SUP_UL31_ mutation did not alter the overall NEC structure.

Nonetheless, we detected several local conformational changes. First, all six copies of UL34 in the NEC-SUP_UL31_ mutant contained an additional density at the C terminus **(S4A Fig)**, corresponding to a portion of the helix ⍺4 that was unresolved in the WT NEC185Δ50 crystal structure [24] but present in the crystal structures of NEC homologs [21,24,25,35–37].

Second, two loops that were partially unresolved in the WT structures due to disorder were resolved in the NEC-SUP_UL31_ mutant: residues 129_UL31_-134_UL31_ (resolved in SUP_AB_, SUP_CD_, SUP_EF_, and SUP_IJ_; partially resolved in SUP_GH_) and 261_UL31_-268_UL31_ (resolved in all six heterodimers) **(Figs 7 and S4B and S6 Table**. The 129_UL31_-134_UL31_ loop was located directly above residue 229_UL31_ that, in turn, was located directly above the heterodimeric globular interface **(Figs 7 and S4B).** In the WT NEC_AB_ structure, the R229_UL31_ side chain made a salt bridge with the nearby D129_UL31_ located at one end of the mostly disordered 129_UL31_-134_UL31_ loop **(Fig 7A)**. The R229L_UL31_ mutation eliminated this salt bridge, and in all SUP_UL31_ heterodimers (except for SUP_KL_ where it was unresolved), the D129_UL31_ side chain pointed away from L229_UL31_ **(Figs 7B and S4B).** The lack of the R229_UL31_-D129_UL31_ salt bridge in the NEC-SUP_UL31_ structure correlated with an ordered 129_UL31_-134_UL31_ loop **(Fig 7B).** We hypothesize that by eliminating the salt bridge, the SUP_UL31_ mutation released the loop, which allowed it to adopt an ordered conformation. Due to the lack of obvious contacts between residue 229_UL31_ and the 261_UL31_-268_UL31_ loop, we postulate that the SUP_UL31_ mutation promoted ordering of the 261_UL31_-268_UL31_ loop through a long-range, allosteric mechanism.

### The SUP_UL31_ mutation generates new contacts at the interhexameric interface

To determine whether the SUP_UL31_ mutation changed any contacts at the lattice interfaces, we analyzed the buried surface areas and sidechain contacts (hydrogen bonds and salt bridges) in the WT NEC and NEC-SUP_UL31_ crystal structures using PDBePISA interface analysis [38]. We found that 5 out of 6 hexameric interfaces in the NEC-SUP_UL31_ crystal lattice had buried surface areas reduced by ~15% compared to the WT NEC hex_AB_ and hex_CD_ lattices **(S12 Table).** These hexameric interfaces also had fewer hydrogen bonds and salt bridges **(S13 Table)** whereas the remaining hexameric interface, A/K, had more contacts than the WT **(S13 Table).** However, the interhexameric interfaces in the NEC-SUP_UL31_ crystal lattice had increased buried surface areas **(S14 Table)** and new contacts **(S15 Table).** Importantly, some of the new contacts at the interhexameric interface were mediated by the 129_UL31_-134_UL31_ and 261_UL31_-268_UL31_ loops that were ordered in the NEC-SUP_UL31_ but not the WT NEC structures **(S6 Table).**

**Fig 8:**
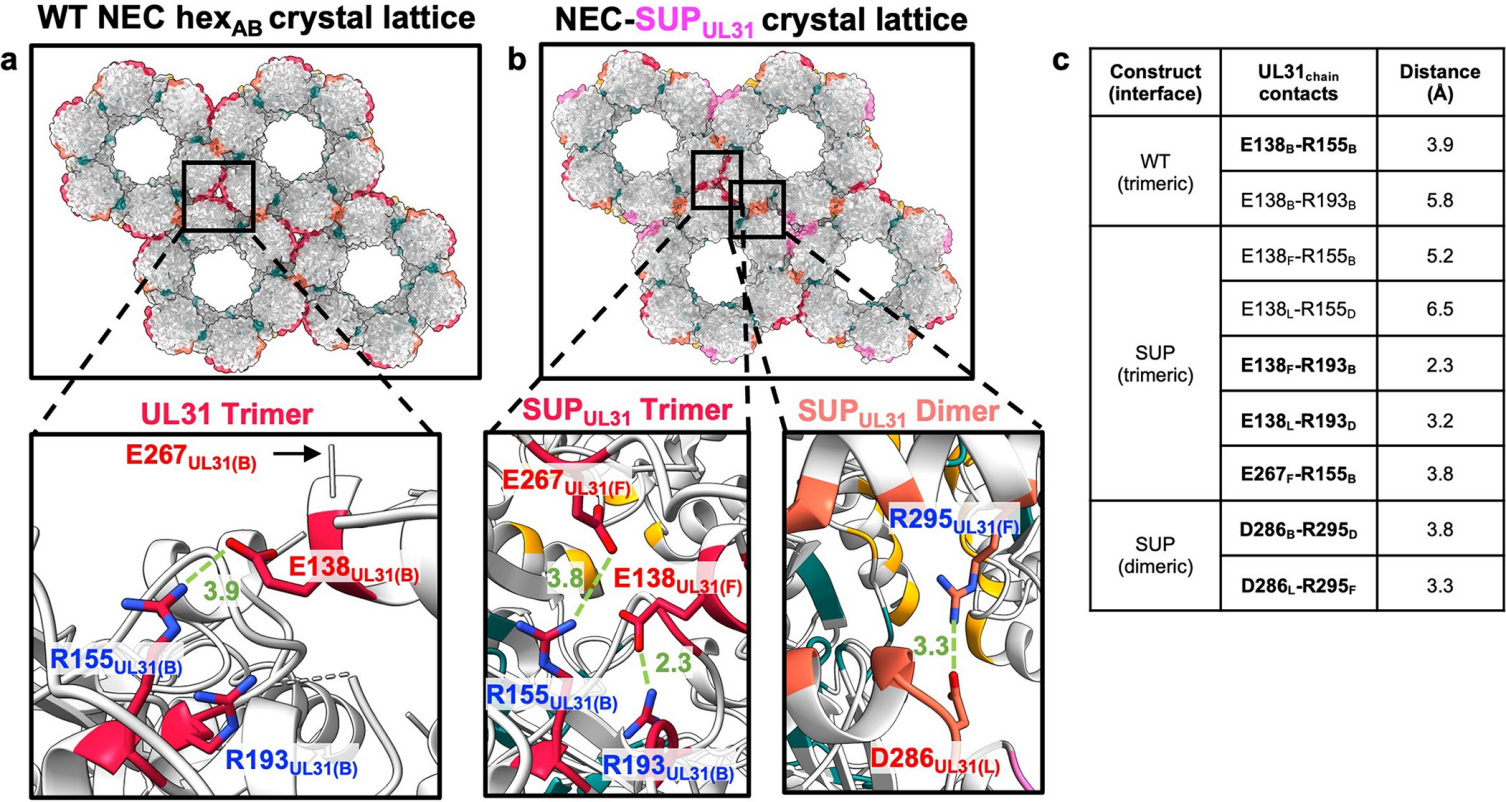
The interhexameric interfaces in the NEC-SUP_UL31_ mutant have new salt bridges. Close-up views of the interhexameric interfaces in the **(a)** WT NEC crystal lattice (UL31 trimer; crimson) and **(b)** NEC-SUP_UL31_ crystal lattice [UL31 trimers (crimson and pink) and dimers (orange and yellow)]. The HSV-1 NEC crystal structure (PDB: 4ZXS) was used to generate the figure in panel **a**. Salt bridges are shown as green dashed lines, with distances in Angstrom. Residues forming salt bridges are shown as sticks and colored in blue (Rs) or red (Es and Ds). **c)** Distances of contacts at the highlighted interfaces. Contacts in bold are salt bridges.

A prominent example of new contacts at the interhexameric interface were several new salt bridges **(Fig 8).** The trimeric interface in the WT NEC hex_AB_ lattice had one salt bridge, E138_UL31_-R155_UL31_ **(Fig 8A).** But in the NEC-SUP_UL31_ lattice, E138_UL31_ and R155_UL31_ formed new salt bridges with other residues. E138_UL31_ formed a salt bridge with R193_UL31_ at both distinct trimeric interfaces, B/F/H and D/J/L **(Fig 8B).** R155_UL31_ formed a salt bridge with E267_UL31_, but only in the B/F/H trimer **(Fig 8B)** (in the D/J/L trimer, R155_UL31_ and E267_UL31_ were ~7 Å apart). In the WT NEC_AB_ structure, the unresolved E267_UL31_ was too far away from the interhexameric interface to participate in any contacts **(Fig 8A)**. Moreover, there was another new salt bridge, D286_UL31_-R295_UL31_, at the dimeric interfaces, B/D and F/L **(Fig 8B),** which was absent from the WT NEC lattice **(Fig 8A)**. Therefore, in the NEC-SUP_UL31_ mutant, there were new contacts at the interhexameric interface that could, in principle, stabilize the NEC lattice in the presence of lattice-destabilizing mutations. We hypothesize that new interhexameric contacts also form in the hex_CD_ lattice, but in the absence of higher resolution data, residues that participate in these contacts remain unknown.

## Discussion

Herpesviruses translocate their capsids from the nucleus to the cytoplasm by an unusual mechanism that requires the formation of membrane-bound coats by the virally encoded heterodimeric complex, the NEC [19,22,23]. The coats are composed of a hexagonal NEC lattice, and mutations that disrupt the lattice interfaces reduce budding *in vitro* [19,24,31] and viral replication [26–28,34]. The hexagonal lattice thus plays a central role in the NEC membrane-budding function. Here, we demonstrated that a suppressor mutation within the UL31 protein, SUP_UL31_, acts as a universal suppressor of membrane budding defects in NEC because it restored efficient membrane budding *in vitro* and viral replication to a broad range of budding-deficient NEC mutants. We hypothesize that the SUP_UL31_ mutation exerts its powerful suppressor effect by reinforcing the hexagonal lattice destabilized by mutations. Indeed, we found that the hexagonal lattice formed by the NEC-SUP_UL31_ maintains a similar geometry yet has new interface contacts. The increased interface would be expected to reinforce the hexagonal NEC lattice, helping it to counteract the lattice-destabilizing effects of mutations. We hypothesize that its dynamic, flexible nature allows the NEC lattice to adapt to perturbations that it likely encounters during nuclear egress.

### The SUP_UL31_ mutation promotes the formation of new interhexameric contacts

Since the SUP_UL31_ mutation rescued budding defects caused by disruptions of the hexagonal lattice, we initially hypothesized that it may do so promoting the formation of a different, potentially, non-hexagonal lattice with distinct interfaces. Instead, we found that NEC-SUP_UL31_ and NEC-DN_UL34_/SUP_UL31_ mutants formed hexagonal lattices both in the crystals and in membrane-bound coats that were very similar to those formed by the WT NEC. However, the interhexameric interfaces in the NEC-SUP_UL31_ crystal lattice are larger due to several new interactions, particularly, salt bridges. How could the SUP_UL31_ mutation promote new contacts at the interhexameric interface? Residue L229_UL31_ itself does not participate in any interface contacts. Instead, we hypothesize that it promotes new contacts through a combination of direct and allosteric mechanisms. By eliminating the R229_UL31_-D129_UL31_ salt bridge **(Fig 7)**, the SUP_UL31_ mutation would release the 129_UL31_-134_UL31_ loop, allowing the latter to adopt an ordered conformation and form new contacts at the interhexameric interface **(Fig 8B).** In the absence of obvious contacts between residue 229_UL31_ and the 261_UL31_-268_UL31_ loop, ordering of the latter probably occurred through a long-range, allosteric mechanism. Larger interhexameric lattice interfaces would be expected to reinforce the lattice. By stabilizing the lattice disrupted by mutations, the SUP_UL31_ mutation could restore efficient budding.

**Fig 9:**
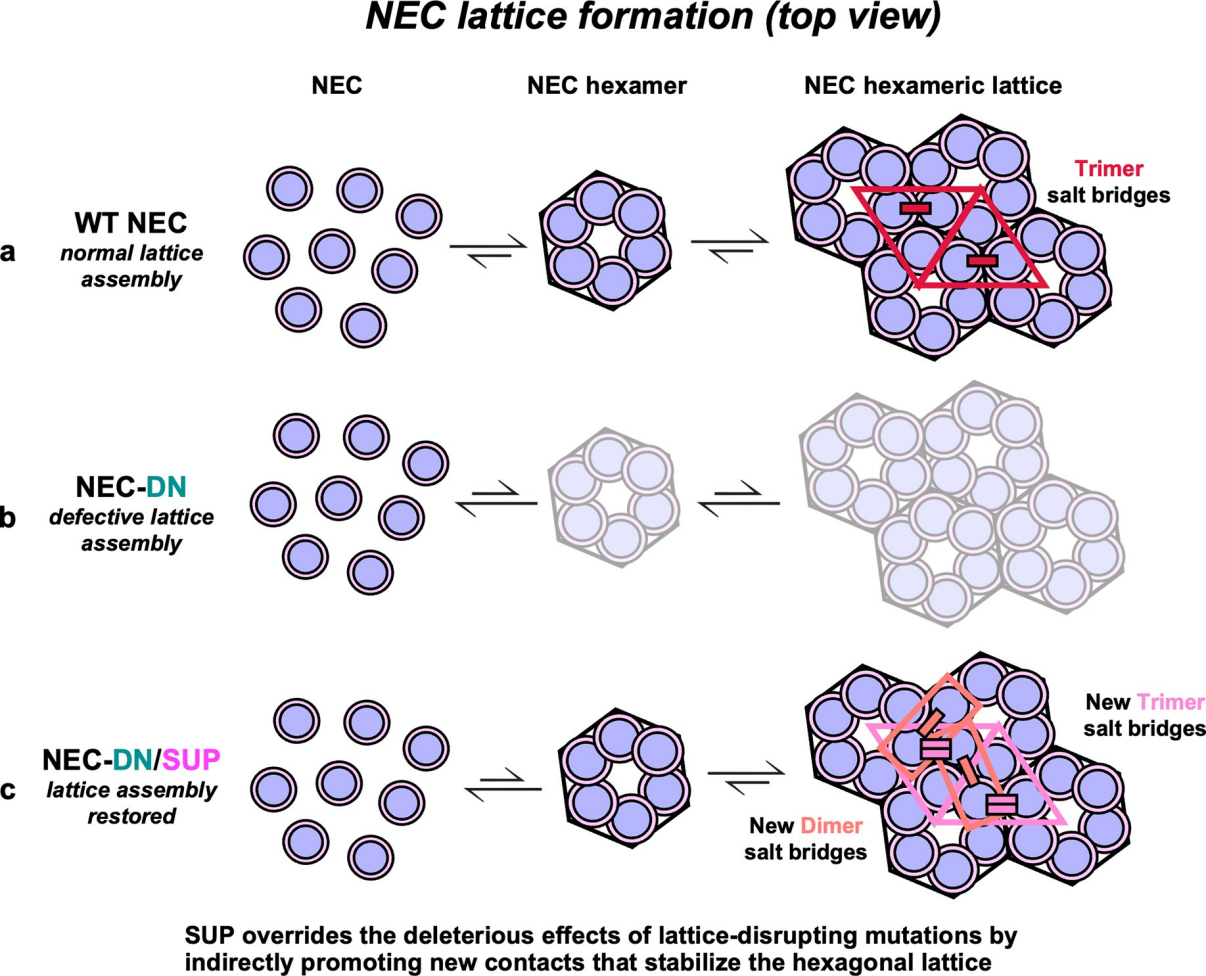
A model of SUP_UL31_ budding restoration in the context of a lattice destabilizing mutation. **a)** WT NEC heterodimers are arranged into hexamers that assemble into a stable hexagonal lattice by forming contacts at the hexameric and interhexameric interfaces. The salt bridges located at the trimeric interhexameric interfaces (crimson) favor lattice association, rather than disassociation. **(b)** In the presence of a lattice destabilizing mutation such as NEC-DN_UL34_, hexamer formation and lattice assembly are perturbed. **(c)** The SUP_UL31_ mutation restores hexagonal lattice formation by promoting the formation of new contacts at both the dimeric and trimeric interhexameric interfaces (pink and salmon), resulting in a stable and functional NEC lattice despite the presence of a destabilizing mutation.

It is easy to envision how the SUP_UL31_ mutation might suppress budding defects caused by mutations that destabilize interhexameric interactions by compensating for the loss of those interactions locally with new interhexameric interactions. However, the SUP_UL31_ mutation also suppresses budding defects of mutants that destabilize the hexamers themselves. Therefore, we propose a more general mechanism for this type of suppression. We hypothesize that the NEC hexamers weakened by interface mutations could be stabilized not only by the interactions between adjacent NEC heterodimers within the hexamer itself but also by their incorporation into a larger lattice where interhexameric contacts would limit the dissociation of NEC heterodimers from the hexamer (**Fig 9**). By strengthening these latter contacts, the SUP_UL31_ mutation could, in principle, compensate for different kinds of lattice defects.

### The SUP_UL31_ mutation could make the NEC more conformationally dynamic

The WT NEC185Δ50 crystallized in the P6 space group, in which the hexamers are perfect, being related by crystallographic symmetry. However, both the NEC185Δ50-SUP_UL31_ and NEC185Δ50-DN_UL34_/SUP_UL31_ mutants crystallized in the C2_1_ space group, in which the hexamers are non-crystallographic and, thus, imperfect. This could imply that the NEC heterodimer became more conformationally dynamic in the presence of the SUP_UL31_ mutation. Indeed, the heterodimeric UL31/UL34 interface between the globular cores of UL31 and UL34 buried a ~6–20% larger surface area in all six NEC-SUP_UL31_ heterodimers compared to the WT NEC heterodimers **(S16 Table),** suggesting flexibility at this interface. Experimental or computational analysis of the NEC dynamics await further studies outside the scope of the current work.

Residue 229_UL31_ is located between two intermolecular salt bridges at the heterodimeric interface formed by the UL31 and UL34 globular cores, R158_UL34_-D232_UL31_ and R167_UL34_-D104_UL31_ **(Figs 7 and S4).** Previous molecular dynamics (MD) study proposed that these salt bridges contribute to the overall stability of the HSV-1 NEC heterodimer [39]. The HCMV NEC, which has only one salt bridge, was found to be more dynamic than HSV-1 in MD simulations [39]. The EBV NEC, which also has only one salt bridge, is a conformationally dynamic heterodimer as revealed by its crystal structure [21]. The number of intermolecular salt bridges at the globular UL31/UL34 interface thus correlates with the stability of the NEC heterodimer.

We propose that the intramolecular salt bridge R229_UL31_-D129_UL31_ is another important contributor to the stability of the NEC heterodimer. Although the NEC-SUP_UL31_ still has two intermolecular salt bridges at the heterodimeric interface, it lacks the intramolecular salt bridge **(Figs 7 and S4)**, which increases its flexibility. This increased flexibility could explain why both NEC-SUP_UL31_ and NEC-DN_UL34_/SUP_UL31_ required extra additives to form crystals relative to the WT NEC [24] and why both NEC-SUP_UL31_ and NEC-DN_UL34_/SUP_UL31_ took the lower symmetry space group C2_1_, rather than P6.

### The NEC forms two distinct hexagonal lattices with unclear biological significance

Thus far, two types of the hexagonal lattice have been observed, hex_AB_ and hex_CD_. HSV-1 NEC formed hex_AB_ (WT-NEC, NEC-SUP_UL31_, and NEC-DN_UL34_/SUP_UL31_) or hex_CD_ (WT-NEC) lattice configurations in crystals and only hex_CD_ lattice in membrane-bound coats. In the case of other crystallized NEC homologs, the HCMV NEC formed a hexagonal lattice resembling the hex_CD_ lattice configuration in crystals [25] whereas the PRV NEC [24,35], the VZV NEC [37], and the EBV NEC [21] did not. Of note, the EBV NEC formed a yet undefined oligomeric assembly on membranes, possibly, due to its observed flexibility [21].

We do not yet know what dictates the choice of a particular lattice for a given NEC homolog or variant under given conditions (e.g., crystals vs. membrane) nor the relative biological significance of the two lattices. One potential reason for the apparent preference for the hex_CD_ lattice on membranes by HSV-1 NEC *in vitro* could be that the cryoET NEC lattice averages, generated from NEC budded vesicles, represent the “end-product” of budding. In this scenario, the hex_AB_ lattice could represent a lattice formed at an intermediate stage of budding, which would not be captured in our cryoET experiments. However, PRV NEC formed a hex_AB_ lattice in budded vesicles formed in uninfected cells overexpressing the NEC [22] and in infected cells [40]. Therefore, it cannot be concluded that the hex_CD_ lattice is preferred on membranes by the NEC or that it is more relevant biologically. We hypothesize that each NEC homolog can form both lattices, perhaps, under different experimental conditions, and that the ability to form two distinct lattices could be due to the inherent plasticity of the NEC. Regardless, how the NEC assembles into a hexagonal lattice of either type is still unknown.

### The SUP_UL31_ mutation acts as a universal suppressor against mutations disrupting the NEC budding activity

The SUP_UL31_ mutation was initially identified as an extragenic suppressor of a nuclear budding defect caused by a double mutation within UL34, D35A_UL34_/E37A_UL34_ (DN_UL34_), in HSV-1 infected cells [27]. Subsequently, we showed that the DN_UL34_ mutation blocks the formation of the hexagonal NEC lattice [19] by eliminating important polar contacts at the hexameric interface [24] and that the SUP_UL31_ mutation restored effective membrane budding *in vitro* to the DN_UL34_ and another hexameric interface mutant, V92F_UL34_ [24]. Here, we found that the SUP_UL31_ mutation could restore budding to many lattice interface mutants (T123Q_UL34_, F252Y_UL31,_ and E153R_UL31_; **Fig 1**); the heterodimeric interface mutants (K137A_UL34_ and K137A/R139A_UL34_; **Fig 3**); and even to a membrane interface mutant (SE6_UL31_; **Fig 4**). Thus, the SUP_UL31_ mutation acts as a universal suppressor mutation that restores efficient budding *in vitro* and, in several cases, viral replication to a diverse range of budding-deficient NEC mutants.

Although mutations that cause budding defects target diverse interfaces, all are expected to destabilize the hexagonal NEC lattice, which is essential for the membrane budding process. The destabilizing effect of the lattice interface mutations is the most apparent. But mutations destabilizing the NEC heterodimer would also be expected to weaken the lattice by destabilizing its core building block. Finally, NEC/membrane interactions likely destabilize the lattice within the membrane-bound NEC coat indirectly. By reinforcing the lattice, the SUP_UL31_ mutation could overcome these lattice destabilizing defects regardless of their nature.

In some cases, the SUP_UL31_ mutation could fully restore the *in-vitro* budding activity but not viral replication, e.g., in the presence of the T123Q_UL34_ and E153R_UL31_ **(Fig 2)**. We hypothesize that these mutations may perturb the NEC functions that do not involve membrane deformation, e.g., nuclear lamina dissolution, capsid docking at the INM, or capsid recruitment. Thus, the SUP_UL31_ mutation specifically restores budding defects.

If the SUP_UL31_ mutation forms a stronger hexagonal lattice, why isn’t this mutation positively selected? We hypothesize that this mutation may impair other functions of the NEC. This idea is supported by the observation that in the *in-trans* complementation experiment, viral replication in the presence of the SUP_UL31_ mutation does not reach the WT levels **(Fig 2).** While the SUP_UL31_ mutation is not positively selected, it provides the virus with a strategy to maintain replication in the presence of external stressors such as inhibitors targeting the NEC. Further work to identify and characterize other herpesviral suppression mechanisms could aid in the advancement of novel antiherpesviral therapeutics.

### A dynamic, flexible lattice may be necessary for NEC function

During nuclear egress, the NEC generates sufficient curvature to wrap around an egressing capsid. The formation of the hexagonal NEC lattice is important for its function in capsid budding. Yet, a perfectly hexagonal lattice is incompatible with spherical curvature. To generate a spherical particle, a hexagonal lattice must contain lattice “defects”, either regular defects, namely, insertions of a different geometry, or irregular defects. Both types of defects have been observed in other viral assembly systems. For example, icosahedral capsids, which are built of hexons, achieve curvature by incorporating pentons at the vertices. A similar strategy could be employed by HSV-1 through the formation of NEC pentamers, which we previously observed *in vitro* [32]. The studies herein add to the growing number of examples of varied NEC oligomeric states and lattice configurations, each of which may be used at varying points during viral replication, as observed for other viruses.

HIV-1 structural protein Gag, which forms the viral capsid and mediates viral budding, presents an interesting case. It assembles into a hexagonal lattice that has irregular defects in the immature HSV-1 capsid [41] but regular, pentameric insertions in the mature capsid [42]. In this way, Gag can “tune” its hexagonal lattice to perform a specific function. Moreover, Gag can form different types of hexagonal lattice. The capsid and matrix domains of Gag each adopted two distinct hexagonal lattices in immature and mature virions, each with a distinct functional role [43,44]. Thus, HSV-1 Gag exemplifies both the conformational dynamics and functional versatility of viral assembly lattices.

Likewise, the hexagonal NEC lattice is conformationally flexible. A recent study that visualized the NEC lattice in cells infected with a mutant pseudorabies virus (PRV), an alphaherpesvirus, by using the focused ion beam/scanning electron cryomicroscopy (cryoFIB/SEM), identified regions of disorder within the hexagonal NEC coats surrounding the capsid [40]. This observation suggests that the NEC lattice can incorporate irregular defects. In addition to irregular defects, non-hexameric NEC assemblies, such as pentamers [32] and heptamers [23], have been observed, albeit not in the context of a wild-type HSV-1 infection. Finally, the NEC can adopt different arrangements of the hexagonal lattice, either by the WT NEC (WT-hex_AB_ and WT-hex_CD_) or by the NEC-SUP_UL31_ mutant presented in this study. We hypothesize that the ability to adopt different configurations allows the NEC to “tune” its lattice to assemble the coats and maintain their integrity in response to perturbations that occur during nuclear egress, such as changes in membrane curvature and interactions with binding partners.

## Materials and methods

### Cells and viruses

Vero and Hep-2 cells were maintained as previously described [11]. The properties of HSV-1(F) and vRR1072(TK+) (a UL34-null virus derived by homologous recombination with HSV-1(F) have also been previously described [11,45]. The UL34-null virus and the UL34-null/SUP_UL31_ recombinant viruses used for complementation assays were derived from the pYEbac102 clone of the HSV-1 strain (F) genome in the bacterial strain GS1783 (a gift from G. Smith, Northwestern U.) [46–48] as previously described. All UL34-null viruses were propagated on Vero tUL34 CX cells that express HSV-1 pUL34 under the control of its native promoter regulatory sequences [49]. Vero tUL34 CX cells were propagated in DMEM high glucose supplemented with 5% fetal bovine serum and the antibiotic penicillin and streptomycin.

### Cloning

All primers used for cloning are listed in **S17 Table**. Cloning of UL31 (1–306), UL34 (1–220), UL34 (15–185), UL34-His_8_ (1–220 with a C-terminal His_8_-tag) and the corresponding UL31 and UL34 mutants [R229L_UL31_ (SUP_UL31_), D35A_UL34_/E37A_UL34_ (DN_UL34_), E37A_UL34_, T123Q_UL34_, F252Y_UL31_, and E153R_UL31_, and S11E_UL31_/S24E_UL31_/S26E_UL31_/S27E _UL31_/S40E_UL31_/S43E_UL31_ (SE6_UL31_)] was previously described [19,24,31].

### Oligomeric interface mutants

Site-directed mutagenesis of pJB14 (UL31 1–306 R229L_UL31_) was performed using splicing-by-overlap extension protocol followed by restriction digest into the pKH90 plasmid (containing an N-terminal His-SUMO-PreScission tag in-frame with a BamHI restriction site) to create either the F252Y_UL31_/R229L_UL31_ (pED20) or E153R_UL31_/R229L_UL31_ (pED21) double mutants.

### Heterodimeric interface mutants

Site-directed mutagenesis of pJB02 (UL34 1–220) was performed using splicing-by-overlap-extension protocol followed by restriction digest into the pJB02 plasmid (containing an N-terminal GST-PreScission tag in-frame with a SalI restriction site) to create either the K137A_UL34_ (pED25), R139A_UL34_ (pED26), or the K137A_UL34_/R139A_UL34_ (pED27) mutants.

### Membrane interface mutants

Site-directed mutagenesis of pJB60 (UL31-SE6_UL31_ 1–306) was performed using an inverse PCR protocol followed by blunt-end ligation to create the SE6_UL31_/R229L_UL31_ mutant (pED45).

### Crystallization constructs

Digested PCR fragments containing R229L_UL31_Δ50–306 were amplified from pJB114 (UL31 1–306 R229L_UL31_) and subcloned by restriction digest into a pET24b(+) plasmid harboring an N-terminal His_6_-SUMO-PreScission tag in-frame with a BamHI restriction site plasmid to create the R229L_UL31_Δ50–306 plasmid (pXG20). Digested PCR fragments containing D35A_UL34_/E37A_UL34_ were amplified from pJB06 (UL34 1–246 D35A_UL34_/E37A_UL34_) and subcloned by restriction digest into a pGEX-6P1 vector containing an N-terminal GST-PreScission tag in-frame with a SalI restriction site to create the UL34 15–185 D35A_UL34_/E37A_UL34_ plasmid (pJB66).

### Cell complementation constructs

Plasmid pRR1072Rep, which was the parent vector for UL34 mutant plasmids used for cell culture complementation assays has been previously described [28]. Mutant derivatives of pRR1072Rep that carry the D35A_UL34_/E37A_UL34_ and K137A_UL34_/R139A_UL34_ double mutations have also been previously described and were referred to as CL04 and CL10 in that publication [28]. Derivatives of pRR1072Rep containing the single D35A_UL34_, E37A_UL34_, T123Q_UL34_, K137A_UL34,_ and R139A_UL34_ were constructed by Gibson assembly. Plasmids were assembled from two PCR products, each generated using pRR1072Rep as a template and using either a mutagenic forward primer paired with a reverse primer from the ampicillin resistance gene, or a mutagenic reverse primer paired with a forward primer from the ampicillin resistance genes. PCR products were digested with DpnI to remove template sequences and then assembled using the New England BioLabs 2X Gibson assembly master mix according to the manufacturer’s instructions.

### Expression and purification of WT NEC, oligomeric interface, and heterodimeric interface mutants

Plasmids encoding HSV-1 UL31 1–306 (pKH90) and UL34 1–220 were co-transformed into *Escherichia coli* BL21(DE3) LoBSTr cells (Kerafast) to generate wild-type NEC220 [19,32]. All mutant constructs contained UL31 1–306 and UL34 1–220 amino acid boundaries. Plasmids encoding the appropriate mutations of either UL31 or UL34 were also co-transformed into *E*. *coli* BL21(DE3) LOBSTR cells (Kerafast) to generate the various NEC oligomeric and heterodimeric interface mutants (listed in **S18 Table**). The expression and purification of NEC220 and some oligomeric interface mutants (NEC-DN_UL34_, NEC-DN_UL34_/SUP_UL31_, NEC-SUP_UL31_, NEC-E37A_UL34_, NEC-T123Q_UL34_, NEC-F252Y_UL31_, and NEC-E153R_UL31_) were described previously [19,24]. The expression and purification of oligomeric interface mutants (NEC-SUP_UL31_/F252Y_UL31_, NEC-SUP_UL31_/E153R_UL31_, and NEC-SUP_UL31_/T123Q_UL34_) and heterodimeric interface mutants (NEC-K137A_UL34_, NEC-R139A_UL34_, NEC-K137A_UL34_/R139A_UL34,_ NEC-K137A_UL34_/SUP_UL31_ and NEC-K137A_UL34_/R139A_UL34_/SUP_UL31_) are described below. Cells expressing the corresponding NEC construct were expressed using auto-induction at 37°C in TB supplemented with 100 μg/mL ampicillin, 100 μg/mL kanamycin, and 34 μg/mL chloramphenicol, 0.2% lactose, and 2 mM MgSO_4_ for 4 h. The temperature was reduced to 25°C for 16 h. Cells were harvested at 5,000 x g for 30 min.

All purification steps were performed at 4°C, as previously described [19,32]. Cell pellets were resuspended in lysis buffer (50 mM Na HEPES pH 7.5, 500 mM NaCl, 1 mM TCEP, and 10% glycerol) supplemented with Complete protease inhibitor (Roche) and lysed using a microfluidizer (Microfluidics). The cell lysate was clarified by centrifugation at 18,000 x g for 40 min and passed over a Ni Sepharose 6 column (Cytiva) equilibrated with lysis buffer. The protein-bound column was washed with 20 mM and 40 mM imidazole lysis buffer and bound proteins were eluted with 250 mM imidazole lysis buffer. Eluted proteins were passed over a Glutathione Sepharose 4B column and washed with lysis buffer. The His_6_-SUMO and GST tags were cleaved for 16 h by PreScission Protease, produced in-house from a GST-PreScission fusion expression plasmid (a gift of Peter Cherepanov, Francis Crick Institute). The protein was passed over 2 x 1 mL HiTrap Talon columns (Cytiva) to remove His_6_-SUMO, followed by injection onto a size-exclusion column (Superdex 75 10/300; Cytiva) equilibrated into gel-filtration buffer (20 mM Na HEPES, pH 7.0, 100 mM NaCl, and 1 mM TCEP), as previously described. Fractions containing pure protein, as assessed by 12% SDS-PAGE and Coomassie staining were pooled and concentrated as described below.

For both NEC-SUP_UL31_ and NEC-DN_UL34_/SUP_UL31_, the cleaved proteins were passed over a HiTrap SP XL (5 mL; Cytiva) ion-exchange column, to remove free His_6_-SUMO. Bound proteins were eluted using a linear salt gradient (60 mL) made from no salt gel filtration buffer (20 mM Na HEPES, pH 7.0, and 1 mM TCEP) and salt gel filtration buffer (20 mM Na HEPES, pH 7.0, 1 M NaCl, and 1 mM TCEP). Proteins typically eluted ~ 360 mM NaCl, at which point the gradient was held constant until the UV signal returned to baseline. Fractions containing pure protein, as assessed by 12% SDS-PAGE and Coomassie staining, were pooled and diluted using no salt gel filtration buffer to reach a 100 mM NaCl concentration, which is required for downstream liposome budding experiments described below. For all purifications described herein, the protein was concentrated to ~ 1 mg/mL and stored at -80°C to prevent degradation observed at 4°C. Protein concentrations were determined by the absorbance at 280 nm. A typical yield was ~0.5 mg/L.

### Expression and purification of membrane interface constructs

Plasmids containing UL31 1–306 (pKH90) and UL34 1-220-His_8_ (pJB57) were co-transformed into *E*. *coli* BL21(DE3) LoBSTr cells (Kerafast) to generate NEC220-His_8_. All the following constructs contained UL31 1–306 and UL34 1–220 amino acid boundaries. A list of plasmids co-transformed to create the NEC-SE6_UL31_-His_8_, NEC-SUP_UL31_-His_8_, and NEC-SE6_UL31_/SUP_UL31_-His_8_ constructs are listed in **S18 Table**. Cells expressing the corresponding NEC mutant were grown using auto-induction at 37°C in TB supplemented with 100 μg/mL ampicillin, 100 μg/mL kanamycin, and 34 μg/mL chloramphenicol, 0.2% lactose, and 2 mM MgSO_4_ for 4 h. The temperature was reduced to 25°C for 16 h. Cells were harvested at 5,000 x g for 30 min.

Cells were resuspended in lysis buffer supplemented with Complete protease inhibitor (Roche) and lysed using a microfluidizer (Microfluidizer). The cell lysate was clarified by centrifugation at 18,000 x g for 40 min and passed over a Ni Sepharose 6 column (Cytiva). The column was washed with 20 mM and 40 mM imidazole lysis buffer and bound proteins were eluted with 250 mM imidazole lysis buffer. Eluted proteins were passed over a Glutathione Sepharose 4B column and washed with lysis buffer. The His_6_-SUMO and GST tags were cleaved for 16 h by PreScission Protease, produced in-house from a GST-PreScission fusion expression plasmid. The protein was loaded on a size-exclusion column (Superdex 75 10/300; Cytiva) equilibrated with a gel-filtration buffer. Fractions containing pure protein, as assessed by 12% SDS-PAGE and Coomassie staining were pooled and concentrated as described above.

### Expression and purification of crystallization constructs

SUP_UL31_Δ50–306 (pXG20) and UL34 15–185 (pJB04) or SUP_UL31_Δ50–306 (pXG20) and UL34 15–185 D35A_UL34_/E37A_UL34_ (pJB66) plasmids were co-transformed into *E*. *coli* BL21(DE3) LoBSTr cells (Kerafast) to produce the NEC-SUP_UL31_ and NEC-DN_UL34_/SUP_UL31_ crystallization constructs, respectively. Cells expressing the corresponding NEC mutant were grown using auto-induction at 37°C in TB supplemented with 100 μg/mL ampicillin, 100 μg/mL kanamycin, and 34 μg/mL chloramphenicol, 0.2% lactose, and 2 mM MgSO_4_ for 4 h. The temperature was reduced to 25°C for 16 h. Cells were harvested at 5,000 x g for 30 min.

Cells were resuspended in lysis buffer supplemented with Complete protease inhibitor (Roche) and lysed using a microfluidizer (Microfluidizer). The cell lysate was clarified by centrifugation at 18,000 x g for 40 min and passed over a Ni Sepharose 6 column (Cytiva). The column was washed with 20 mM and 40 mM imidazole lysis buffer and bound proteins were eluted with 250 mM imidazole lysis buffer. Eluted proteins were passed over a Glutathione Sepharose 4B column and washed with lysis buffer. The His_6_-SUMO and GST tags were cleaved for 16 h by PreScission Protease, produced in-house from a GST-PreScission fusion expression plasmid. The protein was passed over 2 x 1 mL HiTrap Talon columns (Cytiva) to remove His_6_-SUMO, followed by injection onto a size-exclusion column (Superdex 75 10/300; Cytiva) equilibrated into gel-filtration buffer, as previously described. Fractions containing pure protein, as assessed by 12% SDS-PAGE and Coomassie staining were pooled and concentrated as described above.

### *In-vitro* budding assays

Giant unilamellar vesicles (GUVs) were prepared as previously described [19]. For budding quantification, 10 μL of POPC:POPA:POPS = 3:1:1 (Avanti Polar Lipids) GUVs containing 0.2% ATTO-594 DOPE (ATTO-TEC GmbH) fluorescent dye were added to gel filtration buffer containing 0.2 mg/mL (final concentration) Cascade Blue Hydrazide (ThermoFisher Scientific) and either 1 μM WT NEC or NEC mutant (final concentration), for a total sample volume of 100 μL. Reactions incubated for 5 min at 20°C before imaging in 96-well chambered coverglass (Brooks Life Science Systems). Samples were imaged using a Nikon A1R Confocal Microscope with a 60x oil immersion lens at the Imaging and Cell Analysis Core Facility at Tufts University School of Medicine. Budding events were quantified by manually counting ~300 vesicles total in 15 different frames of the sample. Before analysis, the background (GUVs in the absence of NEC) was subtracted from the raw values. All data values are reported in the **S1 Data**. Each sample was tested in at least three biological replicates, each containing three technical replicates. Reported values represent the average budding activity relative to NEC220 or NEC220-His_8_ (100%). The standard error of the mean is reported for each measurement. Significance was calculated using an unpaired one-tailed *t*-test against NEC220. Statistical analyses and data presentation were performed using GraphPad Prism 9.1.0.

### Complementation assays

24-well cultures of Hep-2 cells at 70% confluence were transfected with 0.05 μg of pCMVβ, expressing the β-galactosidase gene, and 0.25 μg of wild-type or mutant UL34 plasmid using Lipofectamine as described by the manufacturer (Gibco-BRL) and incubated at 37°C overnight. The cells were then infected with 10 PFU of the BAC-derived UL34-null virus or UL34-null/SUP_UL31_ virus per cell and incubated at 37°C for 90 min. Monolayers were washed once with pH 3 sodium citrate buffer (50 mM sodium citrate, 4 mM potassium chloride, adjusted to pH 3 with hydrochloric acid) and then incubated at room temperature in fresh citrate buffer for one minute. Cells were washed with V medium (Dulbecco’s modified Eagle’s medium, penicillin-streptomycin, 1% heat-inactivated calf serum) two times. One milliliter of V medium was then added to each well, and after 16 h of incubation at 37°C, cell lysates were prepared by freezing and thawing followed by sonication for 20 seconds at power level 2 with a Fisher sonic dismembrator. The amount of infectivity in each lysate was determined by plaque assay titration on UL34-complementing cells. Part of each cell lysate was assayed for β-galactosidase expression as previously described [28]. Transfection efficiencies in all samples were within 20% of each other. Each sample was tested in at least three biological replicates, each containing one technical replicate. The raw titers and log PFU values for each biological replicate are reported in the **S1 Data**.

### Evaluation of mutant protein expression in mammalian cells

12-well cultures of Hep-2 cells at 70% confluence were transfected with 0.5 μg of wild-type or mutant UL34 or UL31-FLAG plasmid using Lipofectamine as described by the manufacturer (Gibco-BRL) and incubated at 37°C overnight. The cells were then infected with 10 PFU of the corresponding null virus and incubated at 37°C for 90 min. The inoculum was then removed and replaced with 1.5 ml V medium, and cultures were incubated for a further 16 hours. Infected cells were harvested by removing the medium, washing the monolayers once with phosphate-buffered saline (PBS), scraping the cells into 1 ml PBS, and pelleting the cells at 3,000 rpm in the microcentrifuge for 3 minutes. The supernatant liquid was removed, and cell lysates were prepared by resuspending the cell pellets in 25 μl water, adding 25 μl of 2X SDS-polyacrylamide gel sample buffer, and then incubating in a boiling water bath for 10 minutes. Proteins were separated by SDS-PAGE, blotted to nitrocellulose, and then probed with chicken antibody to UL34 (diluted 1:250) [11], mouse antibody to HSV-1 VP5 (diluted 1:500 (Biodesign International) mouse antibody to FLAG epitope (diluted 1:1000) (monoclonal M2, SIGMA/Aldrich) or rabbit antibody to calnexin (diluted 1:1000) (Cell Signaling Technology).

### Crystallization and data collection

Crystals of NEC185-Δ50-SUP_UL31_ were grown by vapor diffusion at 25°C in hanging drops with 1 μL of protein (3 mg/mL), 1 μL of reservoir solution (10% PEG3350, 8 mM Li_2_SO_4_, 6 mM ATP, and 0.1 M MES, pH 6) and 1 μL of Silver Bullets (Hampton Research) reagent [G3 (0.25% 2,2’-Thiodiglycolic acid, 0.2% Azelaic acid, 0.2% Mellitic acid, 0.2% trans-aconitic acid, 0.02 M HEPES sodium pH 6.8)]. Hexagonal SUP_UL31_ crystals appeared after 2 days, only in the presence of Silver Bullets, and were completely grown after one week. Crystals were flash-frozen into liquid nitrogen in a solution identical to the reservoir solution containing 30% glycerol as the cryoprotectant.

Crystals of NEC185-Δ50-DN_UL34_/SUP_UL31_ were grown by vapor diffusion at 25°C in hanging drops with 1 μL of protein (3.5 mg/mL), 1 μL of reservoir solution (10% PEG3350, 14 mM Li2SO4, 14 mM ATP, 10 mM phenol, and 0.1 M MES, pH 6) and 1 μL of Silver Bullets (Hampton Research) reagent [G3 (0.25% 2,2’-Thiodiglycolic acid, 0.2% Azelaic acid, 0.2% Mellitic acid, 0.2% trans-aconitic acid, 0.02 M HEPES sodium pH 6.8)]. Hexagonal DN_UL34_/SUP_UL31_ crystals appeared after one week, only in the presence of Silver Bullets. Crystals were flash-frozen into liquid nitrogen in a solution identical to the reservoir solution containing 30% glycerol as the cryoprotectant. In comparison to the SUP_UL31_ crystals, DN_UL34_/SUP_UL31_ crystals required an additional additive, phenol, and took longer to appear (one week vs. two days).

### CryoEM grid preparation and image collection

A volume of 10 μL of a 1:1 mixture of 400 nm and 800 nm large unilamellar vesicles (LUVs) made of 60% POPC/10% POPS/10% POPA/10% POPE/5% cholesterol/5% Ni-DGS [prepared as previously described [19]] were mixed at room temperature with NEC220-SE6_UL31_-His_8_ to a final protein concentration of 1 mg/mL. After 15 min incubation, 3 μL of the sample was applied to glow-discharged (45 s) Quantifoil (2/2; Electron Microscopy Sciences) grids. Grids were blotted on both sides for 6 s with 0 blotting force and vitrified immediately by plunge freezing into liquid nitrogen-cooled liquid ethane (Vitrobot), before storage in liquid nitrogen. Grids were loaded into a Tecnai F20 transmission electron microscope (FEI) with an FEG and Compustage, equipped with a Gatan Oneview CMOS camera, using a cryo holder (Gatan) (Brandeis University Electron Microscope Facility). The microscope was operated in low-dose mode at 200 keV with SerialEM. Images were recorded at 19,000-fold (pixel size: 5.6 nm) magnification at a defocus of -4 μm. Images are displayed using ImageJ [50].

### CryoET grid preparation

A volume of 10 μL of a 1:1 mixture of 400 nm and 800 nm large unilamellar vesicles (LUVs) made of POPC:POPS:POPA = 3:1:1 [prepared as previously described [19]] was mixed on ice with either WT-NEC220, NEC-SUP_UL31_, or NEC-DN_UL34_/SUP_UL31_, each to a final protein concentration of 1 mg/mL. After 30 min incubation, the sample was mixed with 5 nm fiducial gold beads, and cryoET grids were prepared by applying 3 μL of sample to glow-discharged (30 s) Lacey carbon grids (Electron Microscopy Sciences). Grids were blotted on both sides for 6 s with 0 blotting force and vitrified immediately by plunge freezing into liquid nitrogen-cooled liquid ethane with an FEI Mark IV Vitrobot cryosample plunger. Vitrified cryoET grids were stored in a liquid nitrogen dewar until use.

### CryoET data collection and tomogram reconstruction

Tilt series were collected a Titan Krios electron microscope at the California NanoSystems Institute (CNSI). Data collection parameters are listed in **S11 Table**. Tilt series were collected using SerialEM [51] in a Titan Krios instrument equipped with a Gatan imaging filter (GIF) and a post-GIF K3 direct electron detector in electron-counting mode. Frames in each movie of the raw tilt series were aligned, drift-corrected, and averaged with Motioncor2 [51]. The tilt series micrographs were aligned and reconstructed into 3D tomograms using the IMOD software package [52], then missing-wedge corrected by IsoNet [53] for particle picking.

### Sub-tomographic averaging

The variation in curvature of the NEC hexagonal coat made it difficult to identify the hexagonal repeat units required for particle picking. To overcome this, particle picking was performed using Python scripts derived from the Particle Estimation for Electron Tomography (PEET) software [54]. Firstly, an initial model was generated as previously described [55] by manually picking ~100 particles and performing sub-tomogram averaging using PEET. This allowed for the hexagonal geometric parameter, including the repeating distance and orients, of the NEC lattice to be accurately measured. Secondly, for each tomogram, a small set of particles were manually picked as “seed” particles sparsely covering all areas containing NEC. The “seed” particle set was then expanded by adding unknown particles near each of the known particles based on the hexagonal geometry obtained above. PEET alignment was performed on the expanded particles to match local conformational changes. Finally, the particle set expansion and PEET alignment were performed iteratively to obtain a complete particle set. Particles with less than three neighbors were excluded from the final particle set to remove outliers. Coordinates and orientations of the final particle set were formatted and imported into Relion [56] for further processing. One round of 3D refinement under bin4 pixel size and several rounds of 3D refinement and classification under bin2 pixel size, along with duplicate removal, resulted in the final masked resolutions: WT NEC (5.9 Å), NEC-DN_UL34_/SUP_UL31_ (5.4 Å), and NEC-SUP_UL31_ (13.1 Å). The resolutions reported above for the averaged structures are based on the ‘gold standard’ refinement procedures and the 0.143 Fourier shell correlation (FSC) criterion (**S5 Fig).**

### 3D visualization

UCSF ChimeraX [57] was used to visualize the resulting sub-tomogram averages in their three dimensions, segmentation of density maps, and surface rendering for the different components of NEC.

## Supporting information

S1 Fig Expression levels of WT UL31, WT UL34 and corresponding mutant proteins in Hep-2 cells used for *trans* complementation assays presented in this study. Hep-2 cells were transfected with either wild-type or mutant UL34 **(a)** or UL31-FLAG **(b)** plasmids following by infection with the corresponding null virus. After incubation, cells were harvested, lysed, and analyzed via western blotting with the corresponding antibodies. VP5 and calnexin proteins were used as positive controls.
(PDF)

S2 Fig SDS-PAGE analysis of fractions from size-exclusion chromatography (Superdex S75). **a)** NEC-R139A_UL34_, **b)** NEC-K137A_UL34_, and **c)** NEC-K137A_UL34_/R139A_UL34_. Fractions containing equimolar amounts of UL31 and UL34 (blue), unequal amounts of UL31 and UL34 (green), and free UL34 (magenta) are boxed. Only fractions containing equimolar amounts of UL31 and UL34 (blue) were pooled for use in downstream studies. UL31 is ~34 kDa and UL34 is ~25 kDa.
(PDF)

S3 Fig SDS-PAGE analysis of fractions from size-exclusion chromatography (S75). **a)** NEC-K137A_UL34_/SUP_UL31_ and **b)** NEC-K137A_UL34_/R139A_UL34_/SUP_UL31_. Fractions containing equimolar amounts of UL31 and UL34 (blue), unequal amounts of UL31 and UL34 (green), and free UL34 (magenta) are boxed. Only fractions containing equimolar amounts of UL31 and UL34 (blue) were pooled for use in downstream studies. UL31 is ~34 kDa and UL34 is ~25 kDa.
(PDF)

S4 Fig The six mutant NEC-SUP_UL31_ heterodimers are similar to each other and to the WT NEC heterodimers. **a)** Secondary structure overlay of WT NEC_AB_ and WT NEC_CD_ to the six NEC-SUP_UL31_ heterodimers. Colored circles indicate variable regions in UL31: aa 194–198 (green), 129–134 (peach), and 261–268 (dark purple). The light purple circle indicates additional UL34 C-terminal residues (aa 175–178) resolved in the NEC-SUP_UL31_ heterodimers that were unresolved in the WT NEC structures. **b)** The crystal structures of the WT-NEC_CD,_ NEC-SUP_CD_, NEC-SUP_EF_, NEC-SUP_GH_, NEC-SUP_IJ_, and NEC-SUP_KL_ heterodimers. WT-NEC_AB_ and NEC-SUP_AB_ are shown in **Fig 7**. The position of either R229 in the WT NEC or L229 in the NEC-SUP is shown in magenta. Residues at the UL31/UL34 globular interface are shown in blue. Insets show the resolved portions of the dynamic loops 129_UL31_-134_UL31_ and 261_UL31_-268_UL31_ in peach and dark purple, respectively. The HSV-1 NEC crystal structure (PDB: 4ZXS) was used to generate the figure in **a)** and **b)**.
(PDF)

S5 Fig Directional Fourier shell correlation (FCS) curves for the subtomogram averages of NEC lattices. **a)** WT NEC, **b)** NEC-SUP_UL31_, and **c)** NEC-DN_UL34_/SUP_UL31_.
(PDF)

S1 Table Conditions used for cryoET data collection and processing of WT and mutant NEC coats in this study. (PDF)

S2 Table Data collection and refinement statistics for NEC185Δ50-SUP_UL31_ and NEC185Δ50-DN_UL34_/SUP_UL31_ crystal structures. (PDF)

S3 Table Residues involved in hexameric interactions in the WT NEC_A/B_, WT NEC_C/D_, and NEC-SUP_UL31_ lattices. Interfaces between UL31 and UL34 (boxes shaded in teal) and between UL34 and UL34 (boxes shaded in dark green) were analyzed using PDBePISA analysis [38]. Residues unresolved in the structures are indicated as NR.
(PDF)

S4 Table Conservation of residues at the hexameric interfaces within the NEC-SUP_UL31_ lattice relative to the WT NEC_A_/_B_ and WT NEC_C_/_D_ lattices. Interfaces were analyzed using PDBePISA analysis [38].
(PDF)

S5 Table Conservation of residues at the interhexameric interfaces within the NEC-SUP_UL31_ lattice relative to the WT NEC_A_/_B_ and WT NEC_C_/_D_ lattices. Interface residues were determined using PDBePISA analysis [38].
(PDF)

S6 Table Residues involved in interhexameric (trimeric) interactions in the WT NEC_A_/_B_, WT NEC_C_/_D_, and the NEC-SUP_UL31_ lattices. Interface residues (boxes shaded in light orange) were analyzed using PDBePISA analysis [38]. Residues unresolved in the structures are indicated as NR.
(PDF)

S7 Table Residues involved in interhexameric (dimeric) interactions in the WT NEC_A/B_, WT NEC_C_/_D_, and the NEC-SUP_UL31_ lattices. Interface residues are shaded in light orange (Dimer 1: UL31/UL31 and UL34/UL34) and dark orange (Dimer 2: UL31/UL31). Interfaces were analyzed using PDBePISA analysis [38].
(PDF)

S8 Table Resolved residues for each chain of UL34 (top) and UL31 (bottom) from the NEC185Δ50-SUP_UL31_ crystal structure. Resolved residues, along with % resolved, are listed for each of the chains.
(PDF)

S9 Table Structural alignments of the NEC-SUPUL31 heterodimers in the asymmetric unit. The UL31/UL34 heterodimers and individual UL34 and UL31 chains were aligned. RMSD values (Å) were calculated using “SSM Superpose” in WinCoot [59].
(PDF)

S10 Table Residues involved in heterodimeric interactions in the WT NEC_A/B_, WT NEC_C/D_, and the NEC-SUP_UL31_ heterodimers. Heterodimeric UL31/UL34 interfaces, the hook interface (boxes shaded light green) and the globular interface (boxes shaded in blue) were analyzed using PDBePISA analysis [59]. Residues unresolved in the structures are indicated as NR.
(PDF)

S11 Table Structural alignments of the NEC-SUP_UL31_ to the WT NEC_A_/_B_ and WT NEC_C_/_D_ heterodimers. NEC- SUP_UL31_ heterodimers and individual UL34 and UL31 chains were aligned to the two WT NEC heterodimers and the corresponding WT individual chains. RMSD (Å) values are reported and were calculated using “SSM Superpose” in WinCoot [59].
(PDF)

S12 Table Buried surface areas at the hexameric interfaces. PDBePISA analysis [38] was used to calculate the buried surface areas at the hexameric interfaces (either between UL31/UL34 or UL34/UL34) within the WT NEC_AB_, WT NEC_CD_, and NEC-SUP_UL31_ lattices. The total buried surface area at the hexameric interface was calculated by adding the UL31/UL34 and UL34/UL34 surface areas. For the WT NEC, the RSCB PDB 4ZXS structure was used.
(PDF)

S13 Table Comparison of contacts made at the WT NEC and NEC-SUP_UL31_ lattice hexameric interfaces. Atomic contacts (hydrogen bonds or salt bridges) between heterodimers at the hexameric interfaces (shaded in blue) were analyzed using PDBePISA [38].
(PDF)

S14 Table Buried surface areas at the interhexameric interfaces. PDBePISA analysis [38] was used to calculate the buried surface areas at the interhexameric interfaces (either between UL31 trimers or UL31 dimers) within the WT NEC_AB_, WT NEC_CD_, and NEC-SUP_UL31_ mutant lattices. The total buried surface area at the trimeric interface was calculated by adding the UL31/UL31 surface areas within a trimer. For the WT NEC, the RCSB PDB 4ZXS structure was used.
(PDF)

S15 Table Comparison of contacts made at the interhexameric interfaces in the WT NEC and NEC-SUP_UL31_ lattices. Atomic contacts (hydrogen bonds or salt bridges) between heterodimers at the interhexameric interfaces (shaded in blue) were analyzed using PDBePISA [38].
(PDF)

S16 Table Buried interface surface areas for the globular, hook, and the total heterodimeric interface between UL31 and UL34. PDBePISA analysis [38] was used to calculate the buried surface areas between the globular, hook, and total heterodimer interfaces within the WT NEC_AB_, WT NEC_CD_, and the six NEC-SUP_UL31_ heterodimers. The globular interface area was determined by deleting the UL31 hook region (residues 54–88) prior to the PDBePISA analysis. The hook interface was determined by subtracting the globular area from the total heterodimeric interface area. The total heterodimeric interface area was calculated from the entire crystal structure. For WT NEC, the RSCB PDB 4ZXS structure was used.
(PDF)

S17 Table List of primers used for cloning procedures described in Materials and Methods. All primers are listed in the 5’-3’ direction. Restriction sites are underlined, and mutations are bolded.
(PDF)

S18 Table List of plasmids used to create the NEC constructs used in this study not previously described. (PDF)

S1 Data Source file for the data used to generate Figs 1–4. (XLSX)
