# Supplementary material for: The universal suppressor mutation restores membrane budding defects in the HSV-1 nuclear egress complex by stabilizing the oligomeric lattice
Source: PLoS Pathog. 2024 Jan 16;20(1):e1011936. doi: 10.1371/journal.ppat.1011936 (PMC10817169; doi:10.1371/journal.ppat.1011936)
Supplement: S1 Fig — Hep-2 cells were transfected with either wild-type or mutant UL34 (a) or UL31-FLAG (b) plasmids following by infection with the corresponding null virus. After incubation, cells were harvested, lysed, and analyzed via western blotting with the corresponding antibodies. VP5 and calnexin proteins were used as positive controls. (PDF) [file ppat.1011936.s001.pdf]

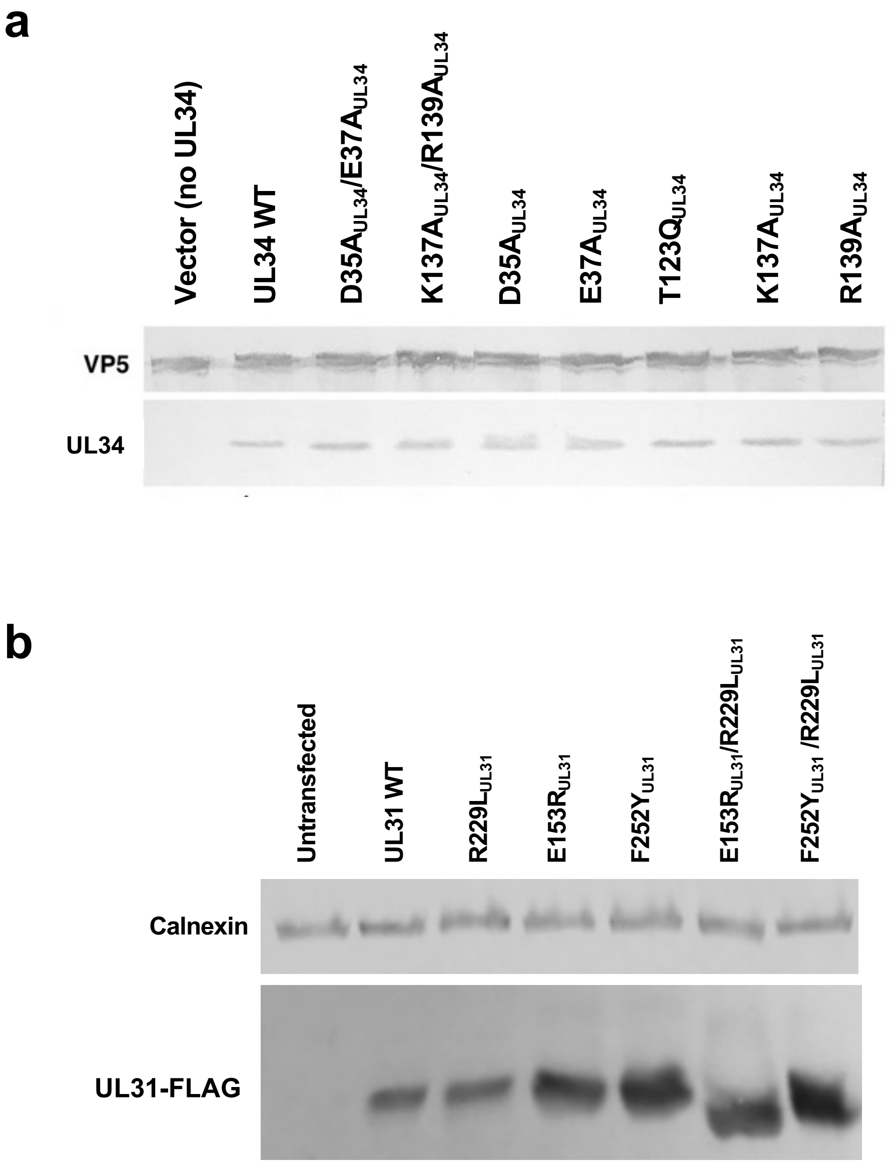

Supplementary Figure S1. Expression levels of WT UL31, WT UL34 and corresponding mutant proteins in Hep-2 cells used for *trans* complementation assays presented in this study. Hep-2 cells were transfected with either wild-type or mutant UL34 **(a)** or UL31-FLAG **(b)** plasmids following by infection with the corresponding null virus. After incubation, cells were harvested, lysed, and analyzed via western blotting with the corresponding antibodies. VP5 and calnexin proteins were used as positive controls.
