# Supplementary material for: The universal suppressor mutation restores membrane budding defects in the HSV-1 nuclear egress complex by stabilizing the oligomeric lattice
Source: PLoS Pathog. 2024 Jan 16;20(1):e1011936. doi: 10.1371/journal.ppat.1011936 (PMC10817169; doi:10.1371/journal.ppat.1011936)
Supplement: S3 Fig — a) NEC-K137AUL34/SUPUL31 and b) NEC-K137AUL34/R139AUL34/SUPUL31. Fractions containing equimolar amounts of UL31 and UL34 (blue), unequal amounts of UL31 and UL34 (green), and free UL34 (magenta) are boxed. Only fractions containing equimolar amounts of UL31 and UL34 (blue) were pooled for use in downstream studies. UL31 is ~34 kDa and UL34 is ~25 kDa. (PDF) [file ppat.1011936.s003.pdf]

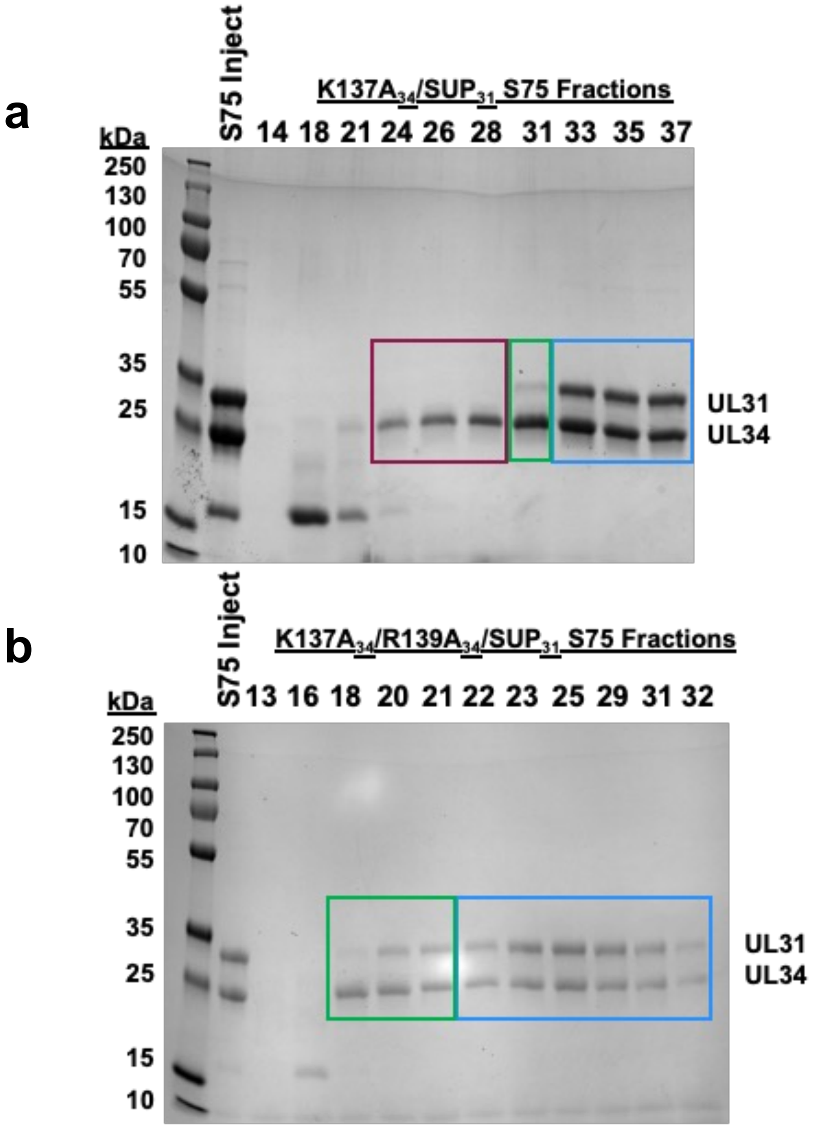

Supplementary Figure S3. SDS-PAGE analysis of fractions from size-exclusion chromatography (S75). **a)** NEC-K137A$_{UL34}$/SUP$_{UL31}$ and **b)** NEC-K137A$_{UL34}$/R139A$_{UL34}$/SUP$_{UL31}$. Fractions containing equimolar amounts of UL31 and UL34 (blue), unequal amounts of UL31 and UL34 (green), and free UL34 (magenta) are boxed. Only fractions containing equimolar amounts of UL31 and UL34 (blue) were pooled for use in downstream studies. UL31 is ~34 kDa and UL34 is ~25 kDa.
