# Supplementary material for: The universal suppressor mutation restores membrane budding defects in the HSV-1 nuclear egress complex by stabilizing the oligomeric lattice
Source: PLoS Pathog. 2024 Jan 16;20(1):e1011936. doi: 10.1371/journal.ppat.1011936 (PMC10817169; doi:10.1371/journal.ppat.1011936)
Supplement: S4 Fig — a) Secondary structure overlay of WT NECAB and WT NECCD to the six NEC-SUPUL31 heterodimers. Colored circles indicate variable regions in UL31: aa 194–198 (green), 129–134 (peach), and 261–268 (dark purple). The light purple circle indicates additional UL34 C-terminal residues (aa 175–178) resolved in the NEC-SUPUL31 heterodimers that were unresolved in the WT NEC structures. b) The crystal structures of the WT-NECCD, NEC-SUPCD, NEC-SUPEF, NEC-SUPGH, NEC-SUPIJ, and NEC-SUPKL heterodimers. WT-NECAB and NEC-SUPAB are shown in Fig 7. The position of either R229 in the WT NEC or L229 in the NEC-SUP is shown in magenta. Residues at the UL31/UL34 globular interface are shown in blue. Insets show the resolved portions of the dynamic loops 129UL31-134UL31 and 261UL31-268UL31 in peach and dark purple, respectively. The HSV-1 NEC crystal structure (PDB: 4ZXS) was used to generate the figure in a) and b). (PDF) [file ppat.1011936.s004.pdf]

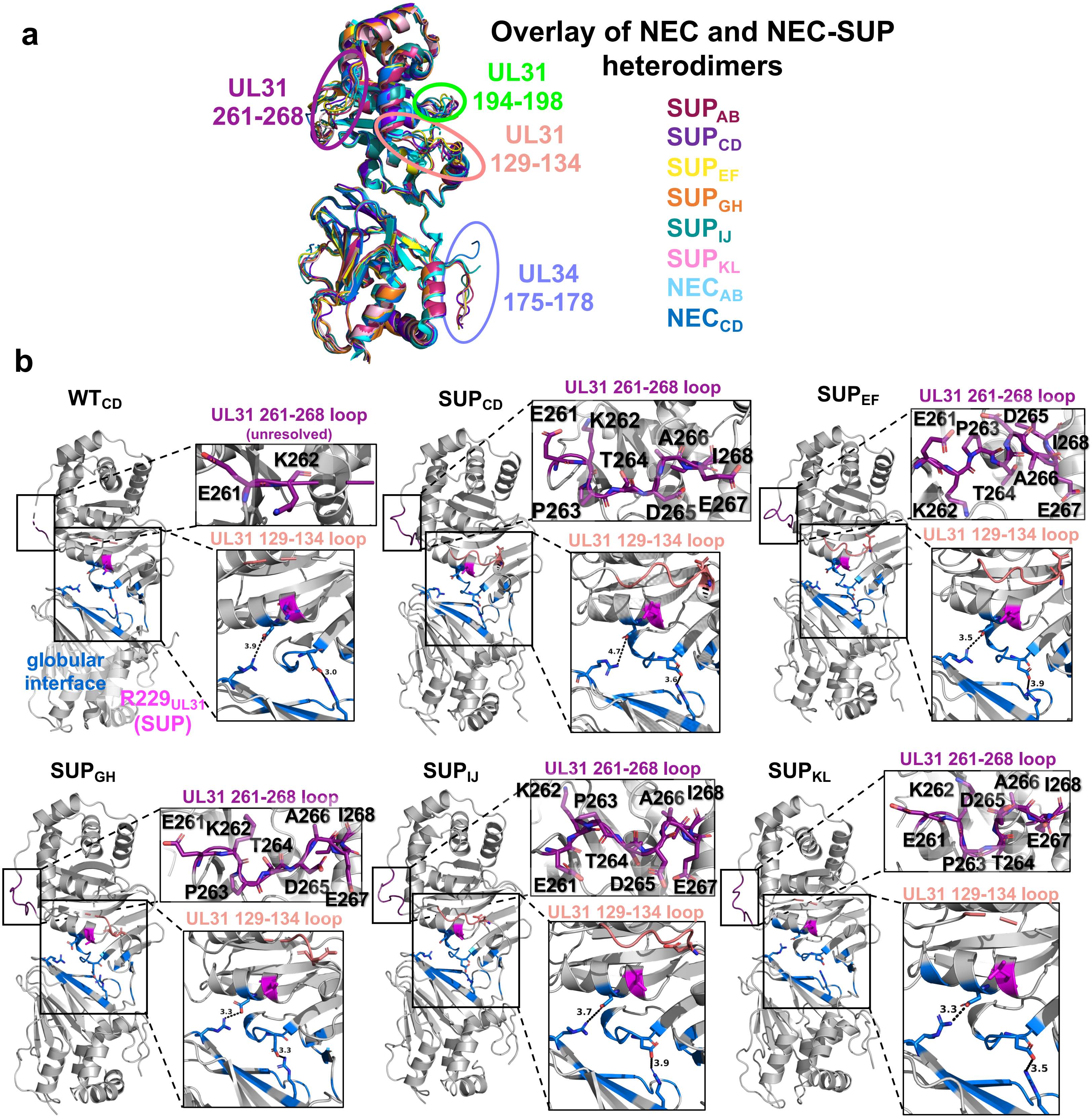

Supplementary Figure S4. The six mutant NEC-SUP$_{UL31}$ heterodimers are similar to each other and to the WT NEC heterodimers. a) Secondary structure overlay of WT NEC$_{AB}$ and WT NEC$_{CD}$ to the six NEC-SUP$_{UL31}$ heterodimers. Colored circles indicate variable regions in UL31: aa 194-198 (green), 129-134 (peach), and 261-268 (dark purple). The light purple circle indicates additional UL34 C-terminal residues (aa 175-178) resolved in the NEC-SUP$_{UL31}$ heterodimers that were unresolved in the WT NEC structures. **b)** The crystal structures of the WT-NEC$_{CD,}$ NEC-SUP$_{CD}$, NEC-SUP$_{EF}$, NEC-SUP$_{GH}$, NEC-SUP$_{IJ}$, and NEC-SUP$_{KL}$ heterodimers. WT-NEC$_{AB}$ and NEC-SUP$_{AB}$ are shown in **Fig. 7**. The position of either R229 in the WT NEC or L229 in the NEC-SUP$_{UL31}$ structures is shown in magenta. Residues at the UL31/UL34 globular interface are shown in blue. Insets show the resolved portions of the dynamic loops 129$_{UL31}$-134$_{UL31}$ and 261$_{UL31}$-268$_{UL31}$ in peach and dark purple, respectively. The HSV-1 NEC crystal structure (PDB: 4ZXS) was used to generate the figure in **a)** and **b)**.
