# Supplementary material for: The universal suppressor mutation restores membrane budding defects in the HSV-1 nuclear egress complex by stabilizing the oligomeric lattice
Source: PLoS Pathog. 2024 Jan 16;20(1):e1011936. doi: 10.1371/journal.ppat.1011936 (PMC10817169; doi:10.1371/journal.ppat.1011936)
Supplement: S5 Fig — a) WT NEC, b) NEC-SUPUL31, and c) NEC-DNUL34/SUPUL31. (PDF) [file ppat.1011936.s005.pdf]

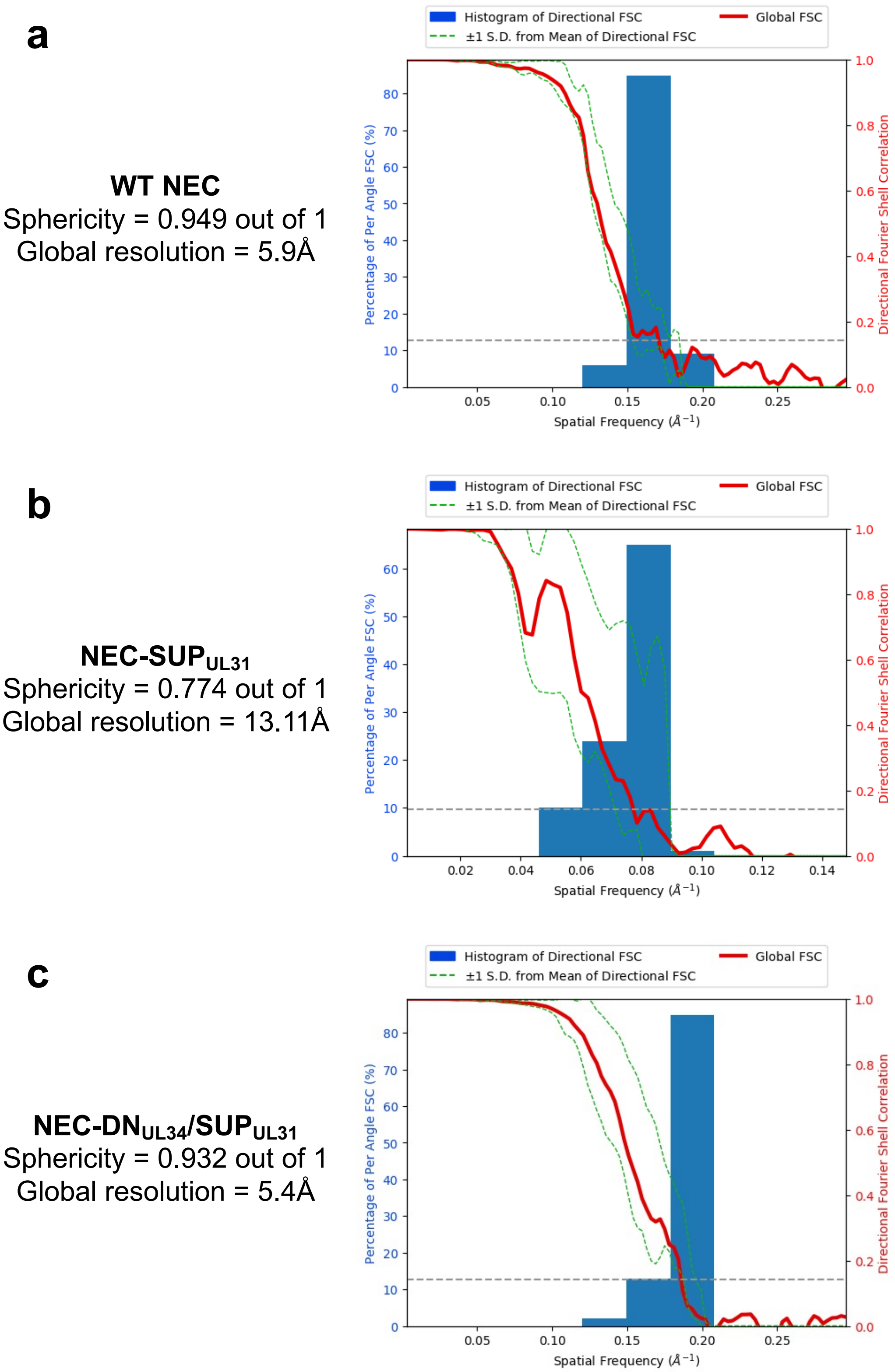

Supplementary Figure S5. Directional Fourier shell correlation (FCS) curves for the subtomogram averages of NEC lattices. a) WT NEC, **b)** NEC-SUP$_{UL31}$, and **c)** NEC-DN$_{UL34}$/SUP$_{UL31}$.
