# Supplementary material for: The universal suppressor mutation restores membrane budding defects in the HSV-1 nuclear egress complex by stabilizing the oligomeric lattice
Source: PLoS Pathog. 2024 Jan 16;20(1):e1011936. doi: 10.1371/journal.ppat.1011936 (PMC10817169; doi:10.1371/journal.ppat.1011936)
Supplement: S1 Table — (PDF) [file ppat.1011936.s006.pdf]

S1 Table. Conditions used for cryoET data collection and processing of WT and mutant NEC coats in this study.

<table>
<tr><th colspan="4">CryoET Data Collection Statistics</th></tr>
<tr><th></th><th>WT-NEC</th><th>NEC-DN$_{UL34}$/SUP$_{UL31}$</th><th>NEC-SUP$_{UL31}$</th></tr>
<tr><th colspan="4">Data collection and processing</th></tr>
<tr><td>Microscope</td><td colspan="3">Titan Krios</td></tr>
<tr><td>Voltage (kV)</td><td colspan="3">300</td></tr>
<tr><td>Total Electron exposure (e–/Å2)</td><td colspan="3">110</td></tr>
<tr><td>Slit width (eV)</td><td colspan="3">20</td></tr>
<tr><td>Detector</td><td colspan="3">K3</td></tr>
<tr><td>Defocus range (µm)</td><td colspan="3">-2.5 to -5.5</td></tr>
<tr><td>Pixel size (Å)</td><td colspan="3">1.69</td></tr>
<tr><td>Software</td><td colspan="3">SerialEM (1)</td></tr>
<tr><td>Tilt-series range</td><td colspan="3">±46°</td></tr>
<tr><td>Tilt-series increment</td><td colspan="3">±2°</td></tr>
<tr><td>Tilt-series scheme</td><td colspan="3">Dose symmetry</td></tr>
<tr><td>EM grid type</td><td colspan="3">Lacey carbon</td></tr>
<tr><td>Tilt-series used</td><td>43</td><td>35</td><td>2</td></tr>
<tr><th colspan="4">Data processing</th></tr>
<tr><td>Software: tilt-series alignment</td><td colspan="3">IMOD (2)</td></tr>
<tr><td>Software: final reconstruction</td><td colspan="3">Relion 4.0 (3)</td></tr>
<tr><td>Initial particle images (no.)</td><td>83385</td><td>48481</td><td>1564</td></tr>
<tr><td>Final particle images (no.)</td><td>34223</td><td>35039</td><td>1390</td></tr>
<tr><td>Final Box-size (pixel)</td><td>196^3</td><td>196^3</td><td>128^3</td></tr>
<tr><td>Pixel size final reconstruction (Å)</td><td>1.69</td><td>1.69</td><td>3.38</td></tr>
<tr><td>Symmetry imposed</td><td colspan="3">C6</td></tr>
<tr><td>Masked map resolution (Å)</td><td>5.9</td><td>5.4</td><td>13.1</td></tr>
<tr><td>FSC threshold</td><td colspan="3">0.143</td></tr>
</table>

References

1. Mastronarde DN. Automated electron microscope tomography using robust prediction of specimen movements. J Struct Biol. 2005;152(1):36-51.

2. Kremer JR, Mastronarde DN, McIntosh JR. Computer visualization of three-dimensional image data using IMOD. J Struct Biol. 1996;116(1):71-6.

3. Scheres SH. RELION: implementation of a Bayesian approach to cryo-EM structure determination. J Struct Biol. 2012;180(3):519-30.
