# Supplementary material for: The universal suppressor mutation restores membrane budding defects in the HSV-1 nuclear egress complex by stabilizing the oligomeric lattice
Source: PLoS Pathog. 2024 Jan 16;20(1):e1011936. doi: 10.1371/journal.ppat.1011936 (PMC10817169; doi:10.1371/journal.ppat.1011936)
Supplement: S2 Table — (PDF) [file ppat.1011936.s007.pdf]

S2 Table. Data collection and refinement statistics for NEC185Δ50-SUP$_{UL31}$ and NEC185Δ50-DN$_{UL34}$/SUP$_{UL31}$ crystal structures.

Parameter	NEC185Δ50-SUP$_{UL31}$[a]	NEC185Δ50-DN$_{UL34}$/SUP$_{UL31}$[a]
Data collection statistics		
Wavelength (Å)	0.9786	0.9786
Space group	$C12_1$	$C12_1$
Unit cell	*a* = 109.555 Å, *b* = 189.103 Å, *c* = 157.096 Å, α = γ = 90°, β = 100.52°	*a* = 110.584 Å, *b* = 190.588 Å, *c* = 157.116 Å, α = γ = 90°, β = 100.42°
Resolution range (Å)	94.55 – 3.92 (4.06 – 3.92)	54.86 – 6.7 (6.94 – 6.70)
No. of reflections		
Total	189091 (18304)	22147 (2243)
Unique	28047 (2801)	5026 (511)
Multiplicity	6.7 (6.5)	4.4 (4.4)
Completeness (%)	99.07 (98.83)	85.87.07 (86.52)
Mean $I/\sigma(I)$	2.83 (1.51)	3.38 (1.67)
Wilson B-factor ($Å^2$)	59.92	153.83
R_{merge}	1.84 (6.109)	0.346 (0.942)
R_{meas}	1.996 (6.637)	0.388 (1.06)
R_{pim}	0.7632 (2.564)	0.1702 (0.475)
$CC_{1/2}$	0.591 (0.312)	0.932 (0.574)
CC*	0.862 (0.689)	0.982 (0.854)
Refinement Statistics		
No. of reflections used		
In refinement	27993 (2795)	
For R_{free}	1996 (199)	
R_{work}[b]	0.2546 (0.3051)	
R_{free}[b]	0.3011 (0.3443)	
CC_{work}	0.873 (0.776)	
CC_{free}	0.865 (0.750)	
No of:		
Nonhydrogen atoms	19341	
Macromolecules	19333	
Ligands	6	
Solvent	2	
Protein Residues	2492	
RMSD		
Bond length (Å)	0.0003	
Bond angle (°)	0.70	
Ramachandran plot[c] *(%)*		
Favored regions	93.67	
Allowed regions	5.72	
Outliers	0.61	

Rotamer outliers (%)	0.00
Clash score	8.33
B-factor	
Average	70.14
Macromolecules	70.13
Ligands	119.26
Solvent	39.49

[a] Highest resolution shell statistics are shown in parentheses.
[b] R_{work} and R_{free} are defined as $\sum||F_{obs}| - |F_{calc}||/\sum|F_{obs}|$ for the reflections in the working or the test set, respectively.
[c] Determined using MolProbity (1).

Reference

1. Williams CJ, Headd JJ, Moriarty NW, Prisant MG, Videau LL, Deis LN, et al. MolProbity: More and better reference data for improved all-atom structure validation. Protein Sci. 2018;27(1):293-315.
