# Supplementary material for: The universal suppressor mutation restores membrane budding defects in the HSV-1 nuclear egress complex by stabilizing the oligomeric lattice
Source: PLoS Pathog. 2024 Jan 16;20(1):e1011936. doi: 10.1371/journal.ppat.1011936 (PMC10817169; doi:10.1371/journal.ppat.1011936)
Supplement: S3 Table — Interfaces between UL31 and UL34 (boxes shaded in teal) and between UL34 and UL34 (boxes shaded in dark green) were analyzed using PDBePISA analysis [38]. Residues unresolved in the structures are indicated as NR. (PDF) [file ppat.1011936.s008.pdf]

S3 Table. Residues involved in hexameric interactions in the WT NEC$_{A/B}$, WT NEC$_{C/D}$, and NEC-SUP$_{UL31}$ lattices. Interfaces between UL31 and UL34 (boxes shaded in teal) and between UL34 and UL34 (boxes shaded in dark green) were analyzed using PDBePISA analysis (1). Residues unresolved in the structures are indicated as NR.

		WT UL34$_A$/UL31$_B$ or UL34$_A$/UL34$_A$	WT UL34$_C$/UL31$_D$ or UL34$_C$/UL34$_C$	SUP UL34$_A$/UL31$_J$ or UL34$_A$/UL34$_I$	SUP UL34$_G$/UL31$_L$ or UL34$_G$/UL34$_K$	SUP UL34$_I$/UL31$_F$ or UL34$_I$/UL34$_E$	SUP UL34$_E$/UL31$_D$ or UL34$_E$/UL34$_C$	SUP UL34$_C$/UL31$_H$ or UL34$_C$/UL34$_G$	SUP UL34$_K$/UL31$_B$ or UL34$_K$/UL34$_A$
**UL31 Residues**	**V87**								
	**T89**								
	**P91**								
	**L94**								
	**S110**								
	**G111**								
	**M112**								
	**G113**								
	**Y114**								
	**Y115**								
	**T222**								
	**H246**								
	**V247**								
	**Q249**								
	**S250**								
	**F252**								
**UL34 Residues**	**G33**								
	**G34**								
	**D35**								
	**E37**								
	**Y41**								
	**S45**								

	**L46**								
	**P47**								
	**S48**								
	**R49**								
	**Q53**								
	**F54**								
	**H55**								
	**Q88**								
	**T90**								
	**G91**								
	**V92**								
	**S93**								
	**L95**								
	**H101**								
	**P103**								
	**H104**								
	**N105**								
	**T112**								
	**P113**								
	**E114**								
	**S122**								
	**T123**								
	**R139**								
	**L140**								
	**G141**								
	**L142**								
	**M159**								
	**P160**								

Reference

1. Krissinel E, Henrick K. Inference of macromolecular assemblies from crystalline state. J Mol Biol. 2007;372(3):774-97.
