# Supplementary material for: The universal suppressor mutation restores membrane budding defects in the HSV-1 nuclear egress complex by stabilizing the oligomeric lattice
Source: PLoS Pathog. 2024 Jan 16;20(1):e1011936. doi: 10.1371/journal.ppat.1011936 (PMC10817169; doi:10.1371/journal.ppat.1011936)
Supplement: S4 Table — Interfaces were analyzed using PDBePISA analysis [38]. (PDF) [file ppat.1011936.s009.pdf]

S4 Table. Conservation of residues at the hexameric interfaces within the NEC-SUP$_{UL31}$ lattice relative to the WT NEC$_{A}$/$_{B}$ and WT NEC$_{C}$/$_{D}$ lattices. Interfaces were analyzed using PDBePISA analysis (1).

	**WT NEC$_{A/B}$: UL34$_A$/UL31$_B$ and UL34$_A$/UL34$_A$ (42 resolved hexameric interface residues)**		
**NEC-SUP Hexameric Interface**	Identical Interface Residues	Total interface residues	% Identical interface residues (compared to WT)
**UL34$_A$/UL31$_J$ and UL34$_A$/UL34$_I$**	35	40	88
**UL34$_G$/UL31$_L$ and UL34$_G$/UL34$_K$**	34	37	92
**UL34$_I$/UL31$_F$ and UL34$_I$/UL34$_E$**	35	38	92
**UL34$_E$/UL31$_D$ and UL34$_E$/UL34$_C$**	33	35	94
**UL34$_C$/UL31$_H$ and UL34$_C$/UL34$_G$**	29	32	91
**UL34$_K$/UL31$_B$ and UL34$_K$/UL34$_A$**	40	42	95
	**WT NEC$_{C/D}$: UL34$_C$/UL31$_D$ and UL34$_C$/UL34$_C$ (39 resolved hexameric interface residues)**		
**NEC-SUP Hexameric Interface**	Identical Interface Residues	Total interface residues	% Identical interface residues (compared to WT)
**UL34$_A$/UL31$_J$ and UL34$_A$/UL34$_I$**	33	40	85
**UL34$_G$/UL31$_L$ and UL34$_G$/UL34$_K$**	36	37	97
**UL34$_I$/UL31$_F$ and UL34$_I$/UL34$_E$**	35	38	92
**UL34$_E$/UL31$_D$ and UL34$_E$/UL34$_C$**	33	35	94
**UL34$_C$/UL31$_H$ and UL34$_C$/UL34$_G$**	28	32	88
**UL34$_K$/UL31$_B$ and UL34$_K$/UL34$_A$**	37	42	95

Reference

1. Krissinel E, Henrick K. Inference of macromolecular assemblies from crystalline state. J Mol Biol. 2007;372(3):774-97.
