# Supplementary material for: The universal suppressor mutation restores membrane budding defects in the HSV-1 nuclear egress complex by stabilizing the oligomeric lattice
Source: PLoS Pathog. 2024 Jan 16;20(1):e1011936. doi: 10.1371/journal.ppat.1011936 (PMC10817169; doi:10.1371/journal.ppat.1011936)
Supplement: S5 Table — Interface residues were determined using PDBePISA analysis [38]. (PDF) [file ppat.1011936.s010.pdf]

**S5 Table. Conservation of residues at the interhexameric interfaces within the NEC-SUP$_{UL31}$ lattice relative to the WT NEC$_{A/B}$ and WT NEC$_{C/D}$ lattices.** Interface residues were determined using PDBePISA analysis (1).

| **Interhexameric Interface** | | **WT UL31$_B$/UL31$_B$/UL31$_B$ (13 total resolved)** | | |
|---|---|---|---|---|
| | | Identical interface residues | Total resolved SUP interface residues | % Identical interface residues (compared to WT) |
| **Trimer 1** (UL31 only) | **SUP UL31$_B$/UL31$_H$/UL31$_F$** | 13 | 26 | 100 |
| **Trimer 2** (UL31 only) | **SUP UL31$_D$/UL31$_J$/UL31$_L$** | 12 | 20 | 92 |
| | | **WT UL31$_D$/UL31$_D$/UL31$_D$ (7 total resolved)** | | |
| **Trimer 1** (UL31 only) | **SUP UL31$_B$/UL31$_H$/UL31$_F$** | 4 | 26 | 57 |
| **Trimer 2** (UL31 only) | **SUP UL31$_D$/UL31$_J$/UL31$_L$** | 3 | 20 | 43 |
| | | **WT UL34$_A$/UL31$_B$ and UL34$_A$/UL31$_B$ (7 total resolved)** | | |
| **Dimer 1** (UL31 and UL34) | **SUP UL34$_A$/UL31$_B$ and UL34$_C$/UL31$_D$** | 6 | 12 | 86 |
| | **SUP UL34$_E$/UL31$_F$ and UL34$_K$/UL31$_L$** | 6 | 7 | 86 |
| | **SUP UL34$_G$/UL31$_H$ and UL34$_I$/UL31$_J$** | 6 | 11 | 86 |
| **Dimer 2** (UL31 only) | **SUP UL31$_B$/UL31$_D$** | 4 | 4 | 100 |
| | **SUP UL31$_F$/UL31$_L$** | 4 | 4 | 100 |
| | **SUP UL31$_H$/UL31$_J$** | 3 | 3 | 100 |
| | | **WT UL34$_C$/UL31$_D$ and UL34$_C$/UL31$_D$ (20 total resolved)** | | |
| **Dimer 1** (UL31 and UL34) | **SUP UL34$_A$/UL31$_B$ and UL34$_C$/UL31$_D$** | 4 | 12 | 20 |
| | **SUP UL34$_E$/UL31$_F$ and UL34$_K$/UL31$_L$** | 4 | 7 | 20 |
| | **SUP UL34$_G$/UL31$_H$ and UL34$_I$/UL31$_J$** | 5 | 11 | 25 |
| **Dimer 2** (UL31 only) | **SUP UL31$_B$/UL31$_D$** | 0 | 4 | 0 |
| | **SUP UL31$_F$/UL31$_L$** | 0 | 4 | 0 |
| | **SUP UL31$_H$/UL31$_J$** | 0 | 3 | 0 |

**Reference**


1. Krissinel E, Henrick K. Inference of macromolecular assemblies from crystalline state. J Mol Biol. 2007;372(3):774-97.
