# Supplementary material for: The universal suppressor mutation restores membrane budding defects in the HSV-1 nuclear egress complex by stabilizing the oligomeric lattice
Source: PLoS Pathog. 2024 Jan 16;20(1):e1011936. doi: 10.1371/journal.ppat.1011936 (PMC10817169; doi:10.1371/journal.ppat.1011936)
Supplement: S6 Table — Interface residues (boxes shaded in light orange) were analyzed using PDBePISA analysis [38]. Residues unresolved in the structures are indicated as NR. (PDF) [file ppat.1011936.s011.pdf]

S6 Table. Residues involved in interhexameric (trimeric) interactions in the WT $NEC_{A}/_{B}$, WT $NEC_{C}/_{D}$, and the NEC-SUP_{UL31} lattices. Interface residues (boxes shaded in light orange) were analyzed using PDBePISA analysis (1). Residues unresolved in the structures are indicated as NR.

		WT $UL31_B$/$UL31_B$/ $UL31_B$	WT $UL31_D$/$UL31_D$/ $UL31_D$	SUP $UL31_B$/$UL31_H$/$UL31_F$ (Trimer 1)			SUP $UL31_D$/$UL31_J$/$UL31_L$ (Trimer 2)		
				B	H	F	D	J	L
	D129		NR	near			near	near	NR
	G130		NR						NR
	R131	NR	NR						NR
	F132	NR	NR		NR				NR
	A133		NR		NR				NR
	A134				NR				
	S136								
	E138								
	A139								
	I141								
	L142								
UL31 Residues	V145								
	Q146								
	N149								
	T150								
	F152								
	E153								
	R155								
	R193								
	G194								
	G195								
	G196								

	**A197**								
	**D199**								
	**E267**	**NR**	**NR**						
	**P269**								
	**D286**								
	**G287**								
	**G288**								

Reference

1. Krissinel E, Henrick K. Inference of macromolecular assemblies from crystalline state. J Mol Biol. 2007;372(3):774-97.
