# Supplementary material for: The universal suppressor mutation restores membrane budding defects in the HSV-1 nuclear egress complex by stabilizing the oligomeric lattice
Source: PLoS Pathog. 2024 Jan 16;20(1):e1011936. doi: 10.1371/journal.ppat.1011936 (PMC10817169; doi:10.1371/journal.ppat.1011936)
Supplement: S7 Table — Interface residues are shaded in light orange (Dimer 1: UL31/UL31 and UL34/UL34) and dark orange (Dimer 2: UL31/UL31). Interfaces were analyzed using PDBePISA analysis [38]. (PDF) [file ppat.1011936.s012.pdf]

S7 Table. Residues involved in interhexameric (dimeric) interactions in the WT NEC$_{A/B}$, WT NEC$_{C/D}$, and the NEC-SUP$_{UL31}$ lattices. Interface residues are shaded in light orange (Dimer 1: UL31/UL31 and UL34/UL34) and dark orange (Dimer 2: UL31/UL31). Interfaces were analyzed using PDBePISA analysis (1).

<table>
<tr><th colspan="2" rowspan="2"></th><th>WT
UL31$_B$/UL31$_B$</th><th>WT
UL31$_D$/UL31$_D$</th><th colspan="2">SUP
UL31$_B$/UL31$_D$</th><th colspan="2">SUP
UL31$_F$/UL31$_L$</th><th colspan="2">SUP
UL31$_H$/UL31$_J$</th></tr>
<tr><th>B</th><th>D</th><th>B</th><th>D</th><th>F</th><th>L</th><th>H</th><th>J</th></tr>
<tr><td rowspan="18">UL31 Residues</td><td>P72</td><td bgcolor="#F8BFAA"></td><td bgcolor="#F8BFAA"></td><td bgcolor="#F8BFAA"></td><td bgcolor="#F8BFAA"></td><td bgcolor="#F8BFAA"></td><td bgcolor="#F8BFAA"></td><td bgcolor="#F8BFAA"></td><td bgcolor="#F8BFAA"></td></tr>
<tr><td>S73</td><td bgcolor="#F8BFAA"></td><td bgcolor="#F8BFAA"></td><td></td><td bgcolor="#F8BFAA"></td><td></td><td bgcolor="#F8BFAA"></td><td bgcolor="#F8BFAA"></td><td bgcolor="#F8BFAA"></td></tr>
<tr><td>E74</td><td></td><td bgcolor="#F8BFAA"></td><td></td><td></td><td></td><td></td><td></td><td></td></tr>
<tr><td>I76</td><td></td><td bgcolor="#F8BFAA"></td><td></td><td></td><td></td><td></td><td bgcolor="#F8BFAA"></td><td></td></tr>
<tr><td>A77</td><td></td><td bgcolor="#F8BFAA"></td><td></td><td></td><td></td><td></td><td></td><td></td></tr>
<tr><td>S81</td><td></td><td bgcolor="#F8BFAA"></td><td></td><td></td><td></td><td></td><td></td><td></td></tr>
<tr><td>N126</td><td></td><td bgcolor="#F8BFAA"></td><td></td><td></td><td></td><td></td><td></td><td></td></tr>
<tr><td>S136</td><td></td><td bgcolor="#F8BFAA"></td><td></td><td></td><td></td><td></td><td></td><td></td></tr>
<tr><td>E138</td><td></td><td bgcolor="#F8BFAA"></td><td></td><td></td><td></td><td></td><td></td><td></td></tr>
<tr><td>A139</td><td></td><td bgcolor="#F8BFAA"></td><td></td><td></td><td></td><td></td><td></td><td></td></tr>
<tr><td>I141</td><td></td><td bgcolor="#F8BFAA"></td><td></td><td></td><td></td><td></td><td></td><td></td></tr>
<tr><td>L142</td><td></td><td bgcolor="#F8BFAA"></td><td></td><td></td><td></td><td></td><td></td><td></td></tr>
<tr><td>V145</td><td></td><td bgcolor="#F8BFAA"></td><td></td><td></td><td></td><td></td><td></td><td></td></tr>
<tr><td>Q146</td><td></td><td bgcolor="#F8BFAA"></td><td></td><td></td><td></td><td></td><td></td><td></td></tr>
<tr><td>P269</td><td></td><td bgcolor="#F8BFAA"></td><td></td><td></td><td></td><td></td><td></td><td></td></tr>
<tr><td>G287</td><td bgcolor="#F59F7A"></td><td></td><td bgcolor="#F59F7A"></td><td></td><td></td><td bgcolor="#F59F7A"></td><td bgcolor="#F59F7A"></td><td bgcolor="#F59F7A"></td></tr>
<tr><td>G288</td><td bgcolor="#F59F7A"></td><td></td><td bgcolor="#F59F7A"></td><td bgcolor="#F59F7A"></td><td bgcolor="#F59F7A"></td><td bgcolor="#F59F7A"></td><td bgcolor="#F59F7A"></td><td bgcolor="#F59F7A"></td></tr>
<tr><td>L291</td><td bgcolor="#F59F7A"></td><td></td><td bgcolor="#F59F7A"></td><td bgcolor="#F59F7A"></td><td bgcolor="#F59F7A"></td><td bgcolor="#F59F7A"></td><td bgcolor="#F59F7A"></td><td bgcolor="#F59F7A"></td></tr>
</table>

	**R295**								
		**WT UL34$_{A}$/UL34$_{A}$**	**WT UL34$_{C}$/UL34$_{C}$**	**SUP UL34$_{A}$/UL34$_{C}$**		**SUP UL34$_{E}$/UL34$_{K}$**		**SUP UL34$_{G}$/UL34$_{I}$**	
				**A**	**C**	**E**	**K**	**G**	**I**
**UL34 Residues**	**P14**								
	**A15**								
	**F16**								
	**E17**								
	**Q21**								
	**R24**								
	**L25**								
	**R32**								
	**G33**								
	**D35**								
	**H55**								
	**H57**								
	**E172**								
	**D173**								
	**A174**								

Reference

1. Krissinel E, Henrick K. Inference of macromolecular assemblies from crystalline state. J Mol Biol. 2007;372(3):774-97.
