# Supplementary material for: The universal suppressor mutation restores membrane budding defects in the HSV-1 nuclear egress complex by stabilizing the oligomeric lattice
Source: PLoS Pathog. 2024 Jan 16;20(1):e1011936. doi: 10.1371/journal.ppat.1011936 (PMC10817169; doi:10.1371/journal.ppat.1011936)
Supplement: S8 Table — Resolved residues, along with % resolved, are listed for each of the chains. (PDF) [file ppat.1011936.s013.pdf]

S8 Table. Resolved residues for each chain of UL34 (top) and UL31 (bottom) from the NEC185Δ50-SUP$_{UL31}$ crystal structure. Resolved residues, along with % resolved, are listed for each of the chains.

Chain ID	Resolved Residues	% Resolved Residues
**UL34**		**Total Residues: 171**
**A**	15-178	96
**C**	15-178	96
**E**	15-178	96
**G**	15-176	94
**I**	15-176	94
**K**	15-177	95
**UL31**		**Total Residues: 255**
**B**	54-306	99
**D**	54-306	99
**F**	54-306	99
**H**	54-131, 135-306	96
**J**	57-306	99
**L**	54-128, 134-306	96
