# Supplementary material for: The universal suppressor mutation restores membrane budding defects in the HSV-1 nuclear egress complex by stabilizing the oligomeric lattice
Source: PLoS Pathog. 2024 Jan 16;20(1):e1011936. doi: 10.1371/journal.ppat.1011936 (PMC10817169; doi:10.1371/journal.ppat.1011936)
Supplement: S9 Table — The UL31/UL34 heterodimers and individual UL34 and UL31 chains were aligned. RMSD values (Å) were calculated using “SSM Superpose” in WinCoot [59]. (PDF) [file ppat.1011936.s014.pdf]

S9 Table. Structural alignments of the NEC-SUP$_{UL31}$ heterodimers in the asymmetric unit. The UL31/UL34 heterodimers and individual UL34 and UL31 chains were aligned. RMSD values (Å) were calculated using "SSM Superpose" in WinCoot (1).

	**$UL34_A$/ $UL31_B$**	**$UL34_C$/ $UL31_D$**	**$UL34_E$/ $UL31_F$**	**$UL34_G$/ $UL31_H$**	**$UL34_I$/ $UL31_J$**	**$UL34_K$/ $UL31_L$**
**$UL34_A$/ $UL31_B$**	--	0.91	0.85	0.86	0.99	0.69
**$UL34_C$/ $UL31_D$**	0.91	--	0.85	0.85	0.96	0.85
**$UL34_E$/ $UL31_F$**	0.85	0.85	--	0.73	0.94	0.72
**$UL34_G$/ $UL31_H$**	0.86	0.85	0.73	--	0.97	0.67
**$UL34_I$/ $UL31_J$**	0.99	0.96	0.94	0.97	--	1.00
**$UL34_K$/ $UL31_L$**	0.69	0.85	0.72	0.67	1.00	--
**UL34**	**Chain A**	**Chain C**	**Chain E**	**Chain G**	**Chain I**	**Chain K**
**Chain A**	--	0.72	0.64	0.75	0.69	0.68
**Chain C**	0.72	--	0.73	0.55	0.75	0.73
**Chain E**	0.64	0.73	--	0.56	0.63	0.59
**Chain G**	0.75	0.55	0.56	--	0.58	0.54
**Chain I**	0.69	0.75	0.63	0.58	--	0.63
**Chain K**	0.68	0.73	0.59	0.54	0.63	--
**UL31**	**Chain B**	**Chain D**	**Chain F**	**Chain H**	**Chain J**	**Chain L**
**Chain B**	--	0.90	0.82	0.84	1.04	0.64
**Chain D**	0.90	--	0.89	0.93	1.02	0.85
**Chain F**	0.82	0.93	--	0.80	1.00	0.69
**Chain H**	0.84	0.93	0.80	--	1.01	0.70
**Chain J**	1.04	1.02	1.00	1.01	--	1.03
**Chain L**	0.64	0.85	0.69	0.70	1.03	--

Reference

1. Emsley P, Lohkamp B, Scott WG, Cowtan K. Features and development of Coot. Acta Crystallogr D Biol Crystallogr. 2010;66(Pt 4):486-501.
