# Supplementary material for: The universal suppressor mutation restores membrane budding defects in the HSV-1 nuclear egress complex by stabilizing the oligomeric lattice
Source: PLoS Pathog. 2024 Jan 16;20(1):e1011936. doi: 10.1371/journal.ppat.1011936 (PMC10817169; doi:10.1371/journal.ppat.1011936)
Supplement: S10 Table — Heterodimeric UL31/UL34 interfaces, the hook interface (boxes shaded light green) and the globular interface (boxes shaded in blue) were analyzed using PDBePISA analysis [59]. Residues unresolved in the structures are indicated as NR. (PDF) [file ppat.1011936.s015.pdf]

S10 Table. Residues involved in heterodimeric interactions in the WT NEC$_{A}$/$_{B}$, WT NEC$_{C}$/$_{D}$, and the NEC-SUP$_{UL31}$ heterodimers. Heterodimeric UL31/UL34 interfaces, the hook interface (boxes shaded light green) and the globular interface (boxes shaded in blue) were analyzed using PDBePISA analysis (1). Residues unresolved in the structures are indicated as NR.

	Residue	WT UL34$_{A}$/UL31$_{B}$	WT UL34$_{C}$/UL31$_{D}$	SUP UL34$_{A}$/UL31$_{B}$	SUP UL34$_{C}$/UL31$_{D}$	SUP UL34$_{E}$/UL31$_{F}$	SUP UL34$_{G}$/UL31$_{H}$	SUP UL34$_{I}$/UL31$_{J}$	SUP UL34$_{K}$/UL31$_{L}$
UL31 Residues	C54	NR	NR					NR	
	L55							NR	
	H56							NR	
	E57								
	R58								
	Q59								
	R60								
	Y61								
	R62								
	L64								
	F65								
	L68								
	A69								
	P72								
	D74								
	E75								
	I76								
	I78								
	V79								
	R80								
	S81								
	L82								
	S83								
	V84								
	P85								
	L86								
	V87								
	T90								
	P91								
	D99								
	Q100								
	T101								
	V102								
	A103								
	D104								
	N105								

	L116								
	I118								
	D232								
	T236								
	K262								
	R22								
	L25								
	I26								
	V27								
	P28								
	P65								
	D67								
	Y68								
	R71								
	L72								
	N74								
	D75								
	A77								
	E78								
	P80								
	C81								
	N82								
	P83								
UL34 Residues	E114								
	R115								
	T116								
	N117								
	V118								
	I119								
	L130								
	G131								
	D134								
	K137								
	L140								
	L142								
	A144								
	P146								
	M147								
	A149								
	S150								
	W152								
	F156								
	R158								

	**R161**								
	**Q163**								
	**L164**								
	**A165**								
	**R167**								
	**F168**								
	**M169**								
	**G170**								
	**P171**								
	**D172**								
	**G175**								

Reference

1. Krissinel E, Henrick K. Inference of macromolecular assemblies from crystalline state. J Mol Biol. 2007;372(3):774-97.
