# Supplementary material for: The universal suppressor mutation restores membrane budding defects in the HSV-1 nuclear egress complex by stabilizing the oligomeric lattice
Source: PLoS Pathog. 2024 Jan 16;20(1):e1011936. doi: 10.1371/journal.ppat.1011936 (PMC10817169; doi:10.1371/journal.ppat.1011936)
Supplement: S11 Table — NEC- SUPUL31 heterodimers and individual UL34 and UL31 chains were aligned to the two WT NEC heterodimers and the corresponding WT individual chains. RMSD (Å) values are reported and were calculated using “SSM Superpose” in WinCoot [59]. (PDF) [file ppat.1011936.s016.pdf]

S11 Table. Structural alignments of the NEC-SUP$_{UL31}$ to the WT NEC$_{A/B}$ and WT NEC$_{C/D}$ heterodimers. NEC- SUP$_{UL31}$ heterodimers and individual UL34 and UL31 chains were aligned to the two WT NEC heterodimers and the corresponding WT individual chains. RMSD (Å) values are reported and were calculated using "SSM Superpose" in WinCoot (1).

**SUP**	**WT UL34$_A$/UL31$_B$**	**WT UL34$_C$/UL31$_D$**
**UL34$_A$/UL31$_B$**	0.94	0.91
**UL34$_C$/UL31$_D$**	0.99	1.02
**UL34$_E$/UL31$_F$**	0.84	0.82
**UL34$_G$/UL31$_H$**	0.93	0.90
**UL34$_I$/UL31$_J$**	0.85	0.83
**UL34$_K$/UL31$_L$**	0.91	0.95
**SUP**	**WT UL34$_A$**	**WT UL34$_C$**
**UL34$_A$**	0.72	0.68
**UL34$_C$**	0.82	0.87
**UL34$_E$**	0.71	0.74
**UL34$_G$**	0.72	0.76
**UL34$_I$**	0.68	0.75
**UL34$_K$**	0.68	0.80
**SUP**	**WT UL31$_B$**	**WT UL31$_D$**
**UL31$_B$**	0.91	0.86
**UL31$_D$**	0.97	0.98
**UL31$_F$**	0.82	0.73
**UL31$_H$**	0.81	0.87
**UL31$_J$**	0.82	0.84
**UL31$_L$**	0.87	0.91

Reference

1. Emsley P, Lohkamp B, Scott WG, Cowtan K. Features and development of Coot. Acta Crystallogr D Biol Crystallogr. 2010;66(Pt 4):486-501.
