# Supplementary material for: The universal suppressor mutation restores membrane budding defects in the HSV-1 nuclear egress complex by stabilizing the oligomeric lattice
Source: PLoS Pathog. 2024 Jan 16;20(1):e1011936. doi: 10.1371/journal.ppat.1011936 (PMC10817169; doi:10.1371/journal.ppat.1011936)
Supplement: S12 Table — PDBePISA analysis [38] was used to calculate the buried surface areas at the hexameric interfaces (either between UL31/UL34 or UL34/UL34) within the WT NECAB, WT NECCD, and NEC-SUPUL31 lattices. The total buried surface area at the hexameric interface was calculated by adding the UL31/UL34 and UL34/UL34 surface areas. For the WT NEC, the RSCB PDB 4ZXS structure was used. (PDF) [file ppat.1011936.s017.pdf]

S12 Table. Buried surface areas at the hexameric interfaces. PDBePISA analysis (1) was used to calculate the buried surface areas at the hexameric interfaces (either between UL31/UL34 or UL34/UL34) within the WT NEC_{AB}, WT NEC_{CD}, and NEC-SUP_{UL31} lattices. The total buried surface area at the hexameric interface was calculated by adding the UL31/UL34 and UL34/UL34 surface areas. For the WT NEC, the RSCB PDB 4ZXS structure was used.

Protein	Chains at the hexameric interface	UL31/UL34 interface area (Å)	UL34/UL34 interface area (Å)	Total hexameric interface area (Å)
WT	$UL34_A/UL31_B$ and $UL34_A/UL34_A$	613	228	842
	$UL34_C/UL31_D$ and $UL34_C/UL34_C$	572	258	830
SUP	$UL34_A/UL31_J$ and $UL34_A/UL34_I$	614	179	793
	$UL34_G/UL31_L$ and $UL34_G/UL34_K$	619	186	805
	$UL34_I/UL31_F$ and $UL34_I/UL34_E$	531	225	756
	$UL34_E/UL31_D$ and $UL34_E/UL34_C$	550	185	735
	$UL34_C/UL31_H$ and $UL34_C/UL34_G$	526	189	715
	$UL34_K/UL31_B$ and $UL34_K/UL34_A$	560	282	842

Reference

1. Krissinel E, Henrick K. Inference of macromolecular assemblies from crystalline state. J Mol Biol. 2007;372(3):774-97.
