# Supplementary material for: The universal suppressor mutation restores membrane budding defects in the HSV-1 nuclear egress complex by stabilizing the oligomeric lattice
Source: PLoS Pathog. 2024 Jan 16;20(1):e1011936. doi: 10.1371/journal.ppat.1011936 (PMC10817169; doi:10.1371/journal.ppat.1011936)
Supplement: S13 Table — Atomic contacts (hydrogen bonds or salt bridges) between heterodimers at the hexameric interfaces (shaded in blue) were analyzed using PDBePISA [38]. (PDF) [file ppat.1011936.s018.pdf]

S13 Table. Comparison of contacts made at the WT NEC and NEC-SUP$_{UL31}$ lattice hexameric interfaces. Atomic contacts (hydrogen bonds or salt bridges) between heterodimers at the hexameric interfaces (shaded in blue) were analyzed using PDBePISA (1).

	UL31 Residue	UL34 Residue	WT A/B	WT C/D	SUP A/J	SUP L/G	SUP F/I	SUP D/E	SUP H/C	SUP B/K
H-bonds	Tyr 114 OH	Thr 90 O	■	■	■		■		■	■
	Tyr 114 OH	Thr 123 OG1			■					
	Leu 86 O	Arg 49 NH1	■			■	■			
	Leu 86 O	Arg 49 NH2	■	■				■	■	
	Lys 88 N	Glu 37 OE2								■
	Thr 89 OG1	Glu 37 OE2		■		■		■		
	Thr 89 OG1	Glu 37 OE1					■		■	
	Thr 89 OG1	Arg 49 NH2				■				
	Thr 89 O	Arg 49 NH1			■					
	Thr 89 N	Glu 37 OE2								■
	Gly 111 O	Arg 49 NH2	■							
	Gly 111 O	Thr 90 OG1			■			■	■	
	Gly 111 O	Ser 93 OG		■			■	■		
	Met 112 O	Gln 53 NE2	■	■		■			■	
	Met 112 SD	His 55 ND1			■					
	Tyr 114 OH	Gly 91 N			■	■		■		
	Ser 250 OG	Gly 91 O				■				■
	Ser 250 OG	Met 159 O							■	
	**UL34 Residue**	**UL34 Residue**	**WT A/A**	**WT C/C**	**SUP A/K**	**SUP E/I**	**SUP G/C**	**SUP G/K**	**SUP C/E**	**SUP A/I**
H- bond	Glu 114 OE2	His 104 N	■							
	Arg 139 O	Ser 48 N	■	■						
	Leu 140 O	Ser 48 N		■						■
	Leu 140 O	Ser 48 OG								■
	Leu 140 O	Tyr 41 OH			■					
	Arg 139 O	Ser 48 OG	■	■						
	Glu 114 OE2	Arg 49 NH1			■					
	Gly 141 O	Tyr 41 OH			■					
	Glu 114 OE1	Asn 105 ND2						■		
Salt bridges	Glu 114 OE2	Arg 49 NH1			■					
	Glu 114 OE2	Arg 49 NH2			■					

Reference

1. Krissinel E, Henrick K. Inference of macromolecular assemblies from crystalline state. J Mol Biol. 2007;372(3):774-97.
