# Supplementary material for: The universal suppressor mutation restores membrane budding defects in the HSV-1 nuclear egress complex by stabilizing the oligomeric lattice
Source: PLoS Pathog. 2024 Jan 16;20(1):e1011936. doi: 10.1371/journal.ppat.1011936 (PMC10817169; doi:10.1371/journal.ppat.1011936)
Supplement: S14 Table — PDBePISA analysis [38] was used to calculate the buried surface areas at the interhexameric interfaces (either between UL31 trimers or UL31 dimers) within the WT NECAB, WT NECCD, and NEC-SUPUL31 mutant lattices. The total buried surface area at the trimeric interface was calculated by adding the UL31/UL31 surface areas within a trimer. For the WT NEC, the RCSB PDB 4ZXS structure was used. (PDF) [file ppat.1011936.s019.pdf]

S14 Table. Buried surface areas at the interhexameric interfaces. PDBePISA analysis (1) was used to calculate the buried surface areas at the interhexameric interfaces (either between UL31 trimers or UL31 dimers) within the WT NEC$_{AB}$, WT NEC$_{CD}$, and NEC-SUP$_{UL31}$ mutant lattices. The total buried surface area at the trimeric interface was calculated by adding the UL31/UL31 surface areas within a trimer. For the WT NEC, the RCSB PDB 4ZXS structure was used.

**Trimeric interface**				
**Construct/interface**	**Chains**		**Surface Area (Å)**	**Total surface area (Å)**
**WT Trimer (AB)**	**UL31$_{B}$/UL31$_{B}$/UL31$_{B}$**	**UL31$_{B}$/UL31$_{B}$**	252	756
		**UL31$_{B}$/UL31$_{B}$**	252	
		**UL31$_{B}$/UL31$_{B}$**	252	
**WT Trimer (CD)**	**UL31$_{D}$/UL31$_{D}$/UL31$_{D}$**	**UL31$_{D}$/UL31$_{D}$**	180	540
		**UL31$_{D}$/UL31$_{D}$**	180	
		**UL31$_{D}$/UL31$_{D}$**	180	
**SUP Trimer 1**	**UL31$_{B}$/UL31$_{H}$/UL31$_{F}$**	**UL31$_{B}$/UL31$_{H}$**	350	1,117
		**UL31$_{F}$/UL31$_{H}$**	358	
		**UL31$_{B}$/UL31$_{F}$**	409	
**SUP Trimer 2**	**UL31$_{D}$/UL31$_{J}$/UL31$_{L}$**	**UL31$_{D}$/UL31$_{L}$**	357	804
		**UL31$_{L}$/UL31$_{J}$**	198	
		**UL31$_{J}$/UL31$_{D}$**	249	
**Dimeric Interface**				
**Interface**	**Construct/chains**	**Total surface area (Å)**		
**Dimer 2** (UL31 only)	**WT UL31$_{B}$/UL31$_{B}$**	107		
	**SUP UL31$_{B}$/UL31$_{D}$**	119		
	**SUP UL31$_{F}$/UL31$_{L}$**	127		
	**SUP UL31$_{H}$/UL31$_{J}$**	96		

Reference

1. Krissinel E, Henrick K. Inference of macromolecular assemblies from crystalline state. J Mol Biol. 2007;372(3):774-97.
