# Supplementary material for: The universal suppressor mutation restores membrane budding defects in the HSV-1 nuclear egress complex by stabilizing the oligomeric lattice
Source: PLoS Pathog. 2024 Jan 16;20(1):e1011936. doi: 10.1371/journal.ppat.1011936 (PMC10817169; doi:10.1371/journal.ppat.1011936)
Supplement: S15 Table — Atomic contacts (hydrogen bonds or salt bridges) between heterodimers at the interhexameric interfaces (shaded in blue) were analyzed using PDBePISA [38]. (PDF) [file ppat.1011936.s020.pdf]

S15 Table. Comparison of contacts made at the interhexameric interfaces in the WT NEC and NEC-SUP$_{UL31}$ lattices. Atomic contacts (hydrogen bonds or salt bridges) between heterodimers at the interhexameric interfaces (shaded in blue) were analyzed using PDBePISA (1).

Interhexameric Contacts							
	Trimer						
	**UL31 Residue**	**UL31 Residue**	**WT A/A/A**	**WT D/D/D**	**SUP B/H/F**	**SUP D/J/L**	
**H-bonds**	Gln 146 NE2	Asn 149 O				DL	
	Glu 138 OE1	Ala 197 N					
	Glu 138 OE2	Ala 197 N			FH		
	Arg 295 NH1	Glu 153 OE2					
	Asp 286 O	Arg 155 NH2					
	Asp 286 OD1	Arg 155 NH2					
	Ser 136 OG	Gly 196 N			BH		
	Ser 136 OG	Gly 195 O				JL	
	Arg 193 NH2	Glu 138 OE1			BF	DL	
	Glu 267 OE2	Arg 155 NH1			BF		
	Gly 130 O	Arg 131 NE			BF		
**Salt bridge**	Glu 138 OE2	Arg 155 NH2					
	Arg 295 NE	Glu 153 OE2					
	Arg 295 NH1	Glu 153 OE1					
	Arg 295 NH1	Glu 153 OE2					
	Arg 295 NH2	Glu 153 OE1					
	Arg 295 NH2	Glu 153 OE2					
	Asp 286 OD1	Arg 155 NH2					
	Asp 286 OD2	Arg 155 NH2					
	Arg 193 NH2	Glu 138 OE1			BF		
	Arg 193 NH2	Glu 138 OE2				DL	
	Glu 267 OE2	Arg 155 NH1			BF		
	Dimer 1						
	**UL31 Residue**	**UL34 Residue**	**WT A/B (none)**	**WT C/D**	**SUP AB/CD (none)**	**SUP EF/KL**	**SUP GH/IJ (none)**
H-bond	Ser 73 N	Glu 17				EL	
	Asp 126 O	Arg 132 NH2					
	Dimer 2						
	**UL31 Residue**	**UL31 Residue**	**WT B/B (none)**	**WT D/D (none)**	**SUP B/D**	**SUP L/F**	**SUP J/H (none)**
H-bond	Asp 286 OD2	Arg 295 NH2					
	Asp 286 OD1	Arg 295 NH2					
Salt bridge	Asp 286 OD1	Arg 295 NH2					
	Asp 286 OD2	Arg 295 NH2					

Reference

1. Krissinel E, Henrick K. Inference of macromolecular assemblies from crystalline state. J Mol Biol. 2007;372(3):774-97.
