# Supplementary material for: The universal suppressor mutation restores membrane budding defects in the HSV-1 nuclear egress complex by stabilizing the oligomeric lattice
Source: PLoS Pathog. 2024 Jan 16;20(1):e1011936. doi: 10.1371/journal.ppat.1011936 (PMC10817169; doi:10.1371/journal.ppat.1011936)
Supplement: S16 Table — PDBePISA analysis [38] was used to calculate the buried surface areas between the globular, hook, and total heterodimer interfaces within the WT NECAB, WT NECCD, and the six NEC-SUPUL31 heterodimers. The globular interface area was determined by deleting the UL31 hook region (residues 54–88) prior to the PDBePISA analysis. The hook interface was determined by subtracting the globular area from the total heterodimeric interface area. The total heterodimeric interface area was calculated from the entire crystal structure. For WT NEC, the RSCB PDB 4ZXS structure was used. (PDF) [file ppat.1011936.s021.pdf]

S16 Table. Buried interface surface areas for the globular, hook, and the total heterodimeric interface between UL31 and UL34. PDBePISA analysis (1) was used to calculate the buried surface areas between the globular, hook, and total heterodimer interfaces within the WT NEC_{AB}, WT NEC_{CD}, and the six NEC-SUP_{UL31} heterodimers. The globular interface area was determined by deleting the UL31 hook region (residues 54-88) prior to the PDBePISA analysis. The hook interface was determined by subtracting the globular area from the total heterodimeric interface area. The total heterodimeric interface area was calculated from the entire crystal structure. For WT NEC, the RSCB PDB 4ZXS structure was used.

NEC Heterodimer	Heterodimeric interface area (Å)	Globular core (Å)	Hook (Å)
WT_{AB}	1764	440	1324
WT_{CD}	1745	430	1315
SUP_{AB}	1773	529	1244
SUP_{CD}	1780	467	1313
SUP_{EF}	1782	492	1290
SUP_{GH}	1767	481	1286
SUP_{IJ}	1742	498	1244
SUP_{KL}	1830	514	1316

Reference

1. Krissinel E, Henrick K. Inference of macromolecular assemblies from crystalline state. J Mol Biol. 2007;372(3):774-97.
