# Supplementary material for: The universal suppressor mutation restores membrane budding defects in the HSV-1 nuclear egress complex by stabilizing the oligomeric lattice
Source: PLoS Pathog. 2024 Jan 16;20(1):e1011936. doi: 10.1371/journal.ppat.1011936 (PMC10817169; doi:10.1371/journal.ppat.1011936)
Supplement: S17 Table — All primers are listed in the 5’-3’ direction. Restriction sites are underlined, and mutations are bolded. (PDF) [file ppat.1011936.s022.pdf]

S17 Table. List of primers used for cloning procedures described in Materials and Methods. All primers are listed in the 5'-3' direction. Restriction sites are underlined, and mutations are bolded.

Primer Name	Primer Sequence (5'-3')	Restriction Site
**Oligomeric Interface Mutants**		
F252Y$_{31}$/R229L$_{31}$ and E153R$_{31}$/R229L$_{31}$ fwd A (KH199)	agcaggatcctatgacaccgaccccccat	SOE/BamHI
F252Y$_{31}$/R229L$_{31}$ rev A (JB178)	ccggaccacgagcac**ata**cgtgctctgccacac	SOE
E153R$_{31}$/R229L$_{31}$ rev A (JB184)	caggaaggcgcgatgcct**gaa**tatcgtgttgatc	SOE
F252Y$_{31}$/R229L$_{31}$ and E153R$_{31}$/R229L$_{31}$ rev B (KH179)	aaatgcggccgcttacggcggaggaaactc	SOE/NotI
F252Y$_{31}$/R229L$_{31}$ fwd B (JB177)	gtgtggcagagcacg**tat**gtgctcgtggtccgg	SOE
E153R$_{31}$/R229L$_{31}$ fwd B (JB183)	gatcaacacgatattc**agg**catcgcgccttcctg	SOE
**Hexameric Interface Mutants**		
K137A$_{34}$, R139A$_{34}$, and K137A$_{34}$/R139A$_{34}$ fwd A (ED058)	aaaaaagtcgacctatggcgggactgggcaagccc	SOE/SalI
K137A$_{34}$ rev A (ED055)	caggccgagccgcccc**cgc**gatggtgtccaggtc	SOE
R139A$_{34}$ rev A (ED057)	ggcatccaggccgag**cgc**cccccttgatggtgtc	SOE
K137A$_{34}$/R139A$_{34}$ rev A (ED061)	ccgggcatccaggccgag**cgc**cccc**cgc**gatggtgtccaggtcgcc	SOE
K137A$_{34}$ fwd B (ED054)	gacctggacaccatc**gcg**gggcggctcggcctg	SOE
R139A$_{34}$ fwd B (ED056)	gacaccatcaagggggg**gcg**ctcggcctggatgcc	SOE
K137A$_{34}$/R139A$_{34}$ fwd B (ED60)	ggcgacctggacaccatc**gcg**gggg**gcg**ctcggcctggatgcccgg	SOE
K137A$_{34}$, R139A$_{34}$, and K137A$_{34}$/R139A$_{34}$ rev B (ED059)	aaaaaagcggccgccttcagtcccccct	SOE/NotI
**Membrane Interface Mutants**		
SE$_{6}$/SUP fwd (ED094)	**tg**ctcatagaccggatgctcaccg	Inverse PCR
SE$_{6}$/SUP fwd (ED054)	**g**gtagtgcaggtgggcggacg	Inverse PCR
**Crystallization Constructs**		
R229L$_{31}$Δ50-306 fwd (KH178)	agcaggatcccaggagctgtgtttacac	BamHI
R229L$_{31}$Δ50-306	aaatgcggccgcttacggcggaggaaactc	NotI

rev (KH178)		
D35A$_{34}$/E37A$_{34}$ 15-185 fwd (JB36)	aaaaaagtcgacctgccttcgagggtctcgttca	SalI
D35A$_{34}$/E37A$_{34}$ 15-185 rev (JB162)	aaaaaagcggccgcctggcggcgcggcaca	NotI
