# Supplementary material for: The universal suppressor mutation restores membrane budding defects in the HSV-1 nuclear egress complex by stabilizing the oligomeric lattice
Source: PLoS Pathog. 2024 Jan 16;20(1):e1011936. doi: 10.1371/journal.ppat.1011936 (PMC10817169; doi:10.1371/journal.ppat.1011936)
Supplement: S18 Table — (PDF) [file ppat.1011936.s023.pdf]

S18 Table. List of plasmids used to create the NEC constructs used in this study not previously described.

Construct	UL31 Plasmid	UL34 Plasmid
Oligomeric Interface Mutants		
NEC-SUP$_{UL31}$/F252Y$_{UL31}$	pED20	pJB02
NEC-SUP$_{UL31}$/E153R$_{UL31}$	pED21	pJB02
NEC-SUP$_{UL31}$/T123Q$_{UL34}$	pJB14	pJB89
Heterodimeric Interface Mutants		
NEC-K137A$_{UL34}$	pKH90	pED25
NEC-R139A$_{UL34}$	pKH90	pED26
NEC-K137A$_{UL34}$/R139A$_{UL34}$	pKH90	pED27
NEC-K137A$_{UL34}$/SUP$_{UL31}$	pJB14	pED25
NEC-K137A$_{UL34}$/R139A$_{UL34}$/SUP$_{UL31}$	pJB14	pED27
Membrane Interface Mutants		
NEC-SE6$_{UL31}$-His	pJB60	pJB57
NEC-SUP$_{UL31}$-His	pJB14	pJB57
NEC-SUP$_{UL31}$/SE6$_{UL31}$-His	pED45	pJB57
