# Supplementary material for: The universal suppressor mutation restores membrane budding defects in the HSV-1 nuclear egress complex by stabilizing the oligomeric lattice
Source: PLoS Pathog. 2024 Jan 16;20(1):e1011936. doi: 10.1371/journal.ppat.1011936 (PMC10817169; doi:10.1371/journal.ppat.1011936)
Supplement: Attachment — Submitted filename: Draganova-SUP-PLOSPath-response-to-criticisms-v9.docx [file ppat.1011936.s025.docx]

**The universal suppressor mutation in the HSV-1 nuclear egress complex restores membrane budding defects by stabilizing the oligomeric lattice**

**Reviewer Comments**

*Three experts in the field have reviewed your manuscript and were in strong agreement that this work is interesting and significant advance in the field. Please note that each reviewer also had requested changes to the text which are considered minor modifications, but important for the final consideration of the manuscript. Please address these minor modifications with detailed response letter in your revised manuscript.*

**Reviewer #1:**

**Summary**

*This paper by Draganova et al. explores mechanisms by which suppressor mutations rescue the function of the HSV-1 NEC complex at the atomic level. The work is interesting as it sheds new light on structural considerations underlying NEC lattice formation and function. The work is thorough and generally well presented. That the suppressor mutation acts to stabilize the lattice by creation of new inter-hexameric binding fits well with its capability to suppress a variety of otherwise destabilizing mutations.*

**Response: We thank the reviewer for their positive assessment of our work and for the opportunity to improve the manuscript.**

**Major Issues: Key Experiments Required for Acceptance**

No Response

**Minor Issues: Editorial and Data Presentation Modifications**

*1. The font used in figure 2 below each panel should be changed to improve readability.*

**Response: The odd look of the font in Fig. 2 appears to be the result of the PDF conversion during manuscript submission. We will make sure that the font looks fine in the printed version.**

*2. Line 203.-204. If UL31 F252Y is expressed to similar levels as other UL31’s, does it fail to complement? Suggesting that its ability to complement is attributable to overexpression is not well supported. They complement well even when expressed at physiological levels (i.e. when placed in recombinant virus).*

**Response: Efficient complementation by UL31 F252Y in trans was surprising considering the reduced budding efficiency of the corresponding recombinant NEC mutant in vitro. Since this mutant is overexpressed in the complementation experiment, we hypothesize that it would fail to complement if expressed at WT UL31 levels. But we do not have experimental evidence for this. Neither have we incorporated this mutation into the virus. Therefore, we have modified the text on lines 212-215 to clarify this point. We have also included the reference to the Western Blot in Fig. S1B showing UL31 expression.**

**Reviewer #2:**

**Summary**

*Draganova et al. present studies arising from a collaboration among the Heldwein, Roller, and Zhou laboratories that studies a mutation in the HSV-1 UL31 gene that was originally found to suppress a mutation in the UL34 gene. These two genes encode the subunits of the viral nuclear egress complex (NEC), whose structure was solved by the Heldwein lab. That structure did not readily explain the suppressor phenotype as the residues altered by the mutations were relatively far from each other. Here, the authors show that the suppressor mutation at least partially restores budding in an in vitro biochemical assay and, in some cases, viral replication in a transfected-infected cells to several different NEC mutants. These mutants either appear to have weaker NEC-NEC interactions for forming a hexagonal lattice or, in one case, are defective for interactions with membranes. Using crystallographic and cryo-EM tomography, the authors show that the suppressor effects are not due to major changes in a single NEC or formation of hexamers, but rather more subtle changes that include formation of new contacts between NEC hexamers within the lattice. Thus, the UL31 mutation has “universal suppressor” activity.*

*The various experiments in this study are well-designed and performed, the data are convincing, and the interpretations, for the most part, are sound. Moreover, the mechanism of suppression is cool and the authors make an interesting case for its relevance to potential anti-NEC therapies. The following comments are meant to improve the manuscript presentation and, in some cases, nudge the authors towards a more cautious take on their results.*

**Response: We thank the reviewer for their positive assessment of our work and for the opportunity to improve the manuscript.**

**Major Issues: Key Experiments Required for Acceptance**

No key new experiments or modifications of existing experiments required.

**Minor Issues: Editorial and Data Presentation Modifications**

*1. Title and elsewhere. Mutations are in genes, not in proteins. Although it can be a bit clumsy, the authors should reword.*

**Response:** **The reviewer is technically correct. We have attempted to modify the text to address this point but found it to be cumbersome. Therefore, we have changed the text in a few places but left it as is in others. We have also provided an explanation for our choice in terminology at the beginning of the Introduction in lines 112-116.**

*2. Abstract. It would be helpful for the reader if the authors more completely summarized the experiments performed and their results rather than just mainly state the conclusions.*

**Response:** **We have included a couple more sentences on lines 40-45 to incorporate this.**

*3. Introduction, first paragraph. Do we know that, for example, molluscan herpesviruses use the same kind of nuclear egress process as herpesviridae? If not, then perhaps just refer to herpesviridae here.*

**Response: To the best of our knowledge, nuclear egress has not yet been investigated in any *Malacoherpesviridae*. However, nuclear egress has been observed in cells infected with Cyprinid Herpesvirus 3, a member of *Alloherpesviridae*, by transmission electron microscopy (doi: 10.1111/j.1365-2761.2007.00850.x). Although the mechanism of this process has not yet been studied, morphological similarities suggest that nuclear egress is not confined to *Herpesviridae*. Therefore, we have left the text as is.**

*4. Also, lines 63-71 of this paragraph lack any citations to the literature or even textbooks.*

**Response: We agree and have now included relevant citations on lines 66-79.**

*5. Fig. 1 legend, line 164. The number of asterisks here don’t conform with what panel 1E seems to show as the in the panel, the mutants showing what looks like the most significant effects have three asterisks, not two.*

**Response: We thank the reviewer for catching this typo and have now corrected it in the legend.**

*6. Line 194. Is this result really surprising? The mutant had only a ~3-fold effect in the biochemical assay and it appears to be overexpressed relative to WT in S1 Fig.*

**Response:** **This finding was surprising to us at the time because we did not yet have the overexpression data. Therefore, we have left the text as is.**

*7. Fig. 2’s statistics require a correction for multiple comparisons. As the authors appropriately used corrections in other figures, perhaps this was just an oversight in drafting the legend, but if not, it may require modifying the figure and possibly some of the conclusions.*

**Response: We thank the reviewer for catching this typo and have now corrected it.**

*8. The complementation assay is very nice, but do the authors know whether they’re only looking at complementation rather than recombination to generate WT virus? For example, have they plaqued their virus on non-permissive cells, too?*

**Response:** **Null virus stocks are routinely titered on both complementing and non-complementing cells and only stocks with a ratio non-complementing titer/complementing titer < 1000 are used for our experiments. When counting plaques in the complementation assays, we use only dilutions that have 20-200 plaques on a well. The contribution of homologous repair viruses to these counts is therefore absent or insignificant.**

*9. Lines 268-270 and S2bc Fig. Why does free UL34 elute earlier from the size-exclusion column than the NEC when it would be expected to be smaller than the NEC. And, if it hadn’t already been stated, do these mutations alter residues near where UL31 and UL34 interact with each other?*

**Response: HSV-1 UL31 and UL34 aggregate when expressed and purified individually. Therefore, the free UL34 eluting before the NEC heterodimer is likely aggregated. There are also higher MW impurities that typically elute with early NEC fractions in the WT purification. Here, those impurities may be co-eluting with the free UL34, but they are typically too diluted to by detected by SDS-PAGE and Coomassie staining.**

**The mutations in question target residues at the UL31/UL34 interface in the heterodimer as described on lines 261-269.**

*10. Line 280, Fig. 3. Is there any hint of quantitative differences in heterodimer stability? If the assay can’t show such differences, the possibility that there could be such differences should be stated.*

**Response: The heterodimer stability was qualitatively assessed by the co-elution of UL31 and UL34 during NEC purification as described on lines 289-298 and 303-307 and shown in Figs. S2 and S3. We are not sure if this could be quantified accurately. Ideally, the affinity of UL31 and UL34 interaction should be quantified using surface plasmon resonance or isothermal titration calorimetry. However, for the reasons described in #9 above, we cannot do so. We considered measuring the intensity of the bands in our SDS-PAGE gel fractions for each purification, but since we did not run all purified fractions on a gel, we would not get a complete representation for how much NEC was purified as a complex. Furthermore, there are some fractions containing impurities that would influence band intensity results. Overall, we think that attempting to quantify the NEC stability by measuring the band intensity would not yield accurate results and may even be misleading. Therefore, our assessment of the NEC stability remains qualitative, for now.**

**To help the reader, we now describe the stability of SUP mutants shown in Fig. S3 on lines 303-307.**

*11. Line 368 and S4 Fig. This is a key point and might be emphasized more by putting it in a text figure and foreshadowing the subsequent discussion.*

**Response: We thank the reviewer for bringing up this excellent point. To emphasize the conformational changes observed within the SUP heterodimer that are relevant for the mechanism of SUP budding restoration and to improve the flow, we have rearranged the sections describing the structures (lines 372-520). We now first describe the cryo-ET structures of the NEC coats to show that globally, the SUP mutation does not change the hexagonal lattice geometry. We then compare the crystal lattices, again to show that globally, the hexagonal lattice is unaffected by the SUP mutation. Finally, we compare the crystal structures up-close, detailing all conformational changes and highlighting mechanistically relevant ones. We have also moved the relevant portion of Fig. S4 to Fig. 7.** **We hope that the edited section is easier to read and that the key points are emphasized.**

*12. Lines 458-472 and elsewhere. The authors could be more cautious about interpreting differences in crystal structures and how they apply to what happens in solution and/or cells. There are many examples of crystal contacts being either irrelevant for those situations or overinterpreted as being relevant. It’s good that the authors use the word “hypothesize” on line 496. Similarly, the authors could be more cautious in the paragraph of lines 559-567.*

**Response: While we agree that crystal contacts generally may not represent biologically relevant interactions, the hexagonal crystal lattice formed the WT HSV-1 NEC certainly recapitulates the NEC lattice formed on budded membranes. This is supported by our mutational and cryo-EM data (Bigalke, et al., Nat. Commun., 2014; Bigalke and Heldwein, EMBO J., 2015). Nonetheless, we agree that the new interactions observed in the NEC-SUP crystal lattice should be interpreted cautiously until confirmed experimentally. Therefore, we revised the text on lines 567-572, 590-593, and 606-609 to clarify which statements are hypotheses.**

*13. Lines 583-593. Along the same lines, aren’t there NEC crystal structures from various herpesviruses that don’t crystallize into hexagonal lattices? The authors might incorporate those findings into their discussion here. See also the paragraph of lines 645-656. 56*

**Response:** **We have included this point on lines 616-618.**

*14. Line 605. Insert “as” between acts and a.*

**Response: We thank the reviewer for catching this typo and have corrected it.**

*16. Lines 629-636. How does the paper from the Heldwein lab suggesting that capsid protein induces pentamer formation fit into this? It seems relevant to the discussion.*

**Response:** **The reviewer brings up a good point. We have now included NEC pentamers into the discussion in more detail on lines 674-678.**

**Reviewer #3:**

**Summary**

*The manuscript by Draganova et al provides an in-depth investigation of an interesting suppressor mutation in the HSV-1 nuclear egress complex (NEC). Some of the strengths of this study include the combination of robust biochemical budding assays, functional complementation assays and both crystallographic and cryo-EM tomography structural studies. Altogether, these approaches provide substantial data to gain a fuller picture of the impact of the SUP-UL31 mutation. These studies lead the authors to propose that the suppressor mutation (SUP-UL31) indirectly stabilizes the formation of hexagonal NEC assemblies at the membrane surface to overcome defects caused by deleterious mutations located at distant sites in the UL31/UL34 NEC. Overall, some of the strongest data for this comes from the in vitro budding assay where robust complementation of multiple defective mutants is observed with the SUP-UL31 mutant and where other potentially confounding factors in the viral complementation assays are absent. Generally, the manuscript is well written although some sections were a bit difficult to follow and I felt that some of the text discussing the structural data could be shortened.*

**Response: We thank the reviewer for their positive assessment of our work and for the opportunity to improve the manuscript.**

**Major Issues: Key Experiments Required for Acceptance**

*1. The SUP-UL31 mutation restores/complements other viral budding mutants but this complementation in the virus titer assays does not reach wt levels - the SUP-UL31 mutant shows reduced titers relative to wt in its own right. This duality makes things a bit more complicated and raises the possibility that other factors are contributing. That said, the in vitro budding assay results are highly consistent with the interpretation that the authors settle on – as the complementation is much more direct and comparable to wt in this assay. I think the authors could discuss this more thoroughly in the final section of the manuscript and provide some explanation and/or evidence for alternative functions of UL31 that might impact virus production but not the in vitro budding assay.*

**Response: The reviewer brings up a valid point. Although the recombinant NEC-SUP has a budding ability on par (if not slightly better than that of the WT NEC) *in vitro*, the UL31 SUP mutant does not trans-complement the replication of the UL31-null HSV-1 as well as the WT UL31. This has led us to propose that UL31 SUP could be defective in some function other that membrane budding.** **We do not yet know what that function might be but have added an additional explanation on lines 658-665.**

*2. Have the authors considered any approaches to more directly measuring the strength of the interhexameric interactions? Is there any evidence for a concentration-dependent assembly in solution that could be followed by light scattering? What about following the concentration dependence of 2D lattice formation on supported lipid bilayers?*

**Response: We have investigated ways to monitor the lattice assembly and to measure its kinetics and thermodynamics but have run into many problems. In solution, the NEC heterodimer is monodisperse and does not form higher-order oligomers such as hexamers, as far as we can tell. Studies of NEC assembly on lipid bilayers have been stymied by non-specific binding, limited protein solubility, and the difficulty in introducing site-specific fluorescent labels. While we are still pursuing this line of inquiry, currently, we cannot monitor the NEC lattice assembly or measure the strength of lattice interactions.**

*3. In general, I felt that the section describing the virus complementation assays (lines 181-234) was difficult to follow and could be rewritten. If the mutant nomenclature could be simplified that might help but I think focusing the text on the direct comparisons that the authors would like to make in the data would also be helpful. The key comparisons that are drawn are comparing the relative effects of transfected mutants (e.g. UL34 mutants) relative to wt across two different virus constructs, which can be confusing to follow (see the next comment for more specific detail).*

*Line 226: “the SUP-UL31 mutation suppressed the complementation defects of both the D35A-UL34/E37A-UL34 227 and E37A-UL34 mutants similarly to the WT UL34 (Fig. 2c). “ I found this phrasing confusing since it relies on a comparison with the relative effects of the mutants in the wt and SUP-UL31 virus backgrounds, across different panels in Fig. 2. I think it may help to first focus on more explicitly explaining how each mutant performs relative to wt in each of the panels and then draw the relative comparisons across the wt and SUP-UL31 viruses. For example, for this line I would say that what is shown is that the D35AUL34/E37AUL34 227 and E37AUL34 mutants behave similarly to wt UL34 in the SUP-UL31 background, which is not the case in the WT-UL31 background. But – and I think this is important to state clearly - in the SUP-UL31 background, using wt UL34 does not yield the same virus titers obtained with wt UL31 virus. The reduced titers that are obtained with the SUP-UL31 virus also reduce the ability to discriminate effects of the mutants. For example, in Fig. 2c, wt UL34 only increases titers by ~10-fold, providing a significantly reduced window to observe the effects of other UL34 mutations. I think the authors need to point out these issues clearly in the accompanying text in this section.*

**Response: We thank the reviewer for pointing out this confusion. We have added a line, as suggested, making the comparison between the lack of complementation of D35A/E37A and E37A by WT UL31 vs. the complementation observed in these same mutants with SUP-UL31 on lines 235-237. The other mutants in this section are also directly compared to the WT for each of conclusions. To improve readability, we edited this section, separating the description of complementation of UL34 vs. UL31 mutants by SUP-UL31.**

**We agree with the reviewer that in Fig. 2c, the WT UL34 titers are lower in the SUP-UL31 background, limiting the dynamic range of our data. We have included in the text when we have drawn conclusions that have an observed reduction of complementation from data that were not calculated as statistically significant (lines 200-203 and 240-241).**

*Line 327: “Previously, we showed that such artificial tethering does not override the requirement for the MPR/membrane interactions and does not restore budding to MPR mutants with budding defects.” Do the authors have an explanation for the lack of an effect of the his-tag since this could be expected to enhance membrane recruitment and assembly? Why would it not complement the SE6 mutations but SUP-UL31 does?*

**Response: The reviewer brings up an excellent point that we have addressed in a previous study (Thorsen *et al., mBio*, 2021). The NEC budding activity requires its membrane proximal regions (MPRs) that facilitate membrane deformation by inserting peripherally into membrane and increasing lipid order. Consequently, the NEC constructs that lack the MPRs do not bud membranes even when tethered to Ni-containing liposomes by means of a polyhistidine tag because such a tag cannot restore the ability to order lipids. The SE6 mutation disrupts proper MPR/membrane interactions cannot be rescued by the polyhistidine tag for the same reason. However, it can be rescued by the SUP mutation. We hypothesize that the disruption of NEC/membrane interactions by the NEC-SE6 mutant likely destabilize the lattice within the membrane-bound NEC coat indirectly. This destabilization can also occur via the other mutants described in this study (e.g. lattice defects or heterodimeric interface disruption). By reinforcing the lattice, the SUP_UL31_ mutation could overcome these lattice destabilizing defects regardless of their nature. We have added an additional explanation on lines 322-324 and 345-355.**

*4. The combination of X-ray and EM tomography is a strength of the analysis of the effect of the SUP-UL31 mutation on the formation of NEC hexamers. But highlights differences between the formation of the different HexAB vs HexCD lattices in truncated proteins in crystals vs full length on membranes. Any speculation as to why the Hex-CD form might be preferred on the membranes?*

**Response: We cannot yet tell what dictates the choice of a particular lattice for a given NEC homolog/variant and condition (crystal vs. membrane) if anything. We envision one potential reason for the apparent preference for the HexCD lattice on membranes by the HSV-1 NEC. The cryoET NEC lattice averages are generated from NEC budded vesicles that represent the “end-product” of budding. One possibility is that the HexAB lattice could represent a lattice formed at an intermediate stage of budding. If so, it would not be captured in our cryoET experiments. However, PRV NEC formed a HexAB lattice in budded vesicles formed in uninfected cells overexpressing the NEC (Hagan et al, Cell, 2016) and in infected cells (Prazak et al, BioRxiv, 2023). Because of this, it cannot be concluded that the HexCD lattice is preferred on budded membranes or that it is more relevant biologically. In the crystals, the WT HSV-1 NEC formed both the HexAB and HexCD lattices (Bigalke and Heldwein, EMBO J, 2015), the SUP and DN/SUP HSV-1 NEC mutants formed only a HexAB lattice (this manuscript), and the HCMV NEC formed the HexCD lattice (Walzer et al, JCB, 2015). Given this variability, at this point, all we can say is that both lattices are possible and that the ability to form two distinct lattices could be due to the inherent plasticity of the NEC. We do not yet know if the ability to form two distinct lattices has any biological significance. Unfortunately, both the HexAB and HexCD lattice interfaces are formed by the same residues (despite having slightly different lattice locations) and cannot be distinguished mutationally. Being able to monitor lattice assembly and measure it kinetics and thermodynamics could help answer this question.**

**We have added these details into the Discussion section on lines 611-631.**

*5. Lines 449-498: Section analyzing the inter-hexameric interfaces. This is a bit confusing since it seems more relevant to understand the Hex-CD interfaces given that these form preferentially on the membranes. How does this detailed analysis of the Hex-AB interfaces relate to what happens on membranes? Is there evidence that both hexamer assemblies form in vivo?*

**Response: We do not have enough evidence to conclude that the HexCD lattice is the biologically more relevant form. See our response to #4. PRV NEC formed a HexAB lattice in budded vesicles formed in uninfected cells overexpressing the NEC (Hagan et al, Cell, 2016) and in infected cells (Prazak et al, BioRxiv, 2023). What lattice is formed by HSV-1 NEC in infected cells is currently unknown.**

*6. I think these sections and the analysis of the hex-AB form could be shortened given that the possibility that the more relevant form could be the Hex-CD assembly. Is there any way to model the role of residue 229 in the Hex-CD assembly, even at low resolution? I think it could be possible to combine last two sections of the paper into one (more concise) section. Some of the associated figures could be moved to supplemental data to contract Fig 7 and 8 into 1 figure that highlights the main points. I think it would be important include the caveat that conclusions on the role of the SUP-UL31 mutation for the Hex-CD form might be different.*

**Response: We do not have enough evidence to conclude that the HexCD lattice is the biologically more relevant form, which we now clarify on lines 611-631. Therefore, focusing our analysis on the HexCD lattice alone is not justified.**

**While we could model residue 229 into the cryoET model, the resolution precludes us from making detailed conclusions regarding the side-chain positioning in a similar manner to the analysis we performed in Fig. 8.**

**The suggestion to condense Fig. 7 and 8 and move some panels into the supplement runs contrary to the suggestion of the reviewer 2 to expand Fig. 7. We hope that by rearranging the description of the cryoET and the crystal structures, we have improved the readability.**

*7. Lines 559-593: “The SUP-UL31 mutation makes the NEC more conformationally dynamic.” I am not convinced that the mutant crystal structures are good evidence for increased flexibility in the NEC. The structural differences could easily be due to the lattice constraints in the two different crystal forms rather than the mutational difference. The fact that the NEC assemblies on the lipids also form a different hexameric form further suggest that one should be cautious in interpreting the crystal structures and their relationship to NEC dynamics.*

**Response:** **We agree with the reviewer in exercising caution when assessing conformational dynamics from a crystal structure. Aside from dynamics, it is unclear how the NEC heterodimers would become flexible enough to adopt an altered lattice to restore budding. We have reworded this section heading to be more speculative on line 583.**

**Minor Issues: Editorial and Data Presentation Modifications**

*8. Figure 1a – resolution of the image is very low?*

**Response:** **The low resolution of Fig. 1 appears to be the result of the PDF conversion during manuscript submission. We will make sure that the image looks fine in the printed version.**

*9. Fig. 2 Bar label formatting needs to be fixed.*

**Response:** **The odd look of the font in Fig. 2 appears to be the result of the PDF conversion during manuscript submission. We will make sure that the image looks fine in the printed version.**

*10. Fig 2b – shouldn't this be UL31-null/WTUL34 complementation?*

**Response: That is correct. Thank you for pointing it out. We have now fixed this in the text.**

*11. Fig. 2d – Is the labeling and the legend correct for this panel? Is this virus UL-34+, UL31WT-neg and SUP-UL31+?*

**Response:** **We thank the reviewer for catching this error and have now corrected it in the figure and within the text. The panel heading should read “WT_UL34_/UL31-null HSV-1 with SUP_UL31_ complementation”. The complementation is performed in trans with a double UL31 mutant plasmid harboring the SUP_UL31_ mutation.**

*12. Fig. 2d - Why does the labeling indicate “Vector (no UL31)”? If this virus expresses UL34-WT and SUP-UL31, why is the vector control titer significantly lower than Panel 2C with the wt-UL34 complementing the UKL34-Null/SUP-UL31 virus?*

**Response:** **As the reviewer pointed out in the above comment, there was an error in the labeling of panel 2d. This label has been fixed and control “Vector (no UL31)” should now be appropriate for this experiment in a UL31-null background.**

*13. The NEC-SUPUL31 crystal structure is at relatively low resolution (3.9Å). Could the authors speak to the accuracy of the coordinates at this resolution relative to the RMSD values that they calculate?*

**Response: The resolution of a crystal structure is not a coordinate error. Coordinate errors are much lower than the resolution and depend on the individual atomic thermal displacement factors. Calculating coordinate errors is very complicated and beyond the scope of this study. An RMSD <1Å^2^ is generally considered to indicate similar structures.**
